# Risk factors for acute kidney injury following transcatheter aortic valve replacement: a systematic review and meta-analysis

**DOI:** 10.3389/fcvm.2026.1684953

**Published:** 2026-03-02

**Authors:** Nan Jiang, Xue Liu, Jiwei Huang, Yi Jiang, Dan Li

**Affiliations:** 1 Department of Cardiology, The First People’s Hospital of Zigong, Zigong, Sichuan, China; 2 Department of Oncology, The Second People’s Hospital of Yibin, Yibin, Sichuan, China

**Keywords:** AKI, meta-analysis, risk factors, systematic review, TAVR

## Abstract

**Objective:**

To delineate risk factors for acute kidney injury (AKI) after transcatheter aortic valve replacement (TAVR) via a systematic review and meta-analysis.

**Methods:**

PubMed, Embase, the Cochrane Library, and Web of Science were searched through February 2025 for case-control studies reporting post-TAVR AKI. Two reviewers independently performed study selection, data extraction, and bias assessment. Pooled analyses were conducted with Stata 15.0.

**Results:**

Thirty-four studies (10,353 patients) met the inclusion criteria; 2,250 patients (21.7%) developed AKI. Univariable meta-analysis implicated multiple comorbid, hemodynamic, and procedural factors [e.g., hypertension, diabetes, coronary and peripheral vascular disease, porcelain aorta, prior PCI, atrial fibrillation, chronic kidney disease (CKD), advanced NYHA class, left ventricular ejection fraction (LVEF) <40%, anemia, diuretic use, transapical/transaortic access, general anesthesia, rapid pacing, bleeding or vascular complications, transfusion, and peri-procedural myocardial infarction or stroke; all *p* < 0.05). Multivariable pooling isolated eight independent predictors: hypertension (OR 2.87), coronary artery disease (1.46), peripheral vascular disease (1.71), prior stroke (1.61), CKD (3.27), elevated serum creatinine (2.80), higher STS score (1.06 per point), and transapical access (3.45). Publication bias was not detected.

**Conclusions:**

Post-TAVR AKI is chiefly driven by cardiovascular comorbidity and renal impairment, with hypertension, coronary and peripheral vascular disease, prior stroke, CKD, elevated creatinine, high STS score, and transapical access displaying the strongest, independent associations. Awareness of these factors may facilitate peri-procedural risk stratification and targeted renal-protective strategies.

## 1 Introduction

According to a statistical report from the American Heart Association (AHA) on heart disease and stroke, the number of elderly patients with calcific aortic stenosis in the United States and Europe is projected to more than double by 2050. Currently, over 3% of adults aged 65 and older are affected by varying degrees of aortic valve stenosis (1, 2). Currently, there is no evidence that medical therapy can slow the progression or treat aortic stenosis (AS). Aortic valve replacement (AVR) remains the only effective treatment for patients with severe AS who are symptomatic or have left ventricular dysfunction. Initially developed as an alternative to surgical aortic valve replacement (SAVR) for high-risk surgical candidates, transcatheter aortic valve replacement (TAVR) has, over the past two decades, evolved into one of the most commonly performed structural heart interventions. Numerous high-quality randomized clinical trials (RCTs) have confirmed the safety and efficacy of TAVR. Its indications have since expanded beyond patients at high or intermediate surgical risk (3–7). Studies have shown that in low-risk patients, TAVR is associated with lower short-term procedural risk and reduced long-term follow-up costs, with no significant differences compared to SAVR in terms of long-term mortality or the combined risk of death and stroke (8, 9). Evidence suggests that early TAVR may be superior to clinical surveillance even in asymptomatic patients with severe AS (2). Globally, the number of TAVR procedures has surpassed that of surgical valve replacements.

With significant technological advancements and increased operator experience, TAVR-now a complex yet standardized minimally invasive endovascular procedure-has shown a steady decline in perioperative complication rates. Common complications and clinical endpoints include mortality, stroke, bleeding and transfusion, acute kidney injury, vascular and access-related complications, structural cardiac complications, new-onset conduction disturbances, and arrhythmias. Acute kidney injury (AKI) following either TAVR or SAVR is a serious complication associated with poor prognosis. It correlates with increased mortality, higher risk of infection, and more complex hospitalization (10). Due to variations in AKI definitions and reference standards across studies, reported incidence rates of AKI after TAVR have varied widely. Based on the Valve Academic Research Consortium (VARC) criteria, the incidence ranges from 4.6% to 35.1% (11). In comparable studies, data from the U.S. National Inpatient Sample indicated an AKI rate of 11.5% (20,045 out of 173,760 patients), while the Asia Pacific Interventional Cardiology Registry reported an incidence of 6.8% [77 out of 1,125 Asian patients undergoing Transcatheter Aortic Valve Implantation(TAVI)] (12, 13). A prospective multicenter study from Italy reported that one in six patients undergoing TAVR developed AKI, which was associated with a twofold increase in all-cause mortality at one-year follow-up (14). Multiple studies have identified post-TAVR AKI as an independent risk factor for both short- and long-term mortality (15, 16), making it a focal point of ongoing research in this field. Particularly noteworthy is the situation regarding non-elective TAVR—including urgent, emergent, and salvage cases—which accounts for approximately 10% to 24% of procedures (17). This proportion is increasing and tends to be clinically underestimated in practice. Compared with elective TAVR, it is associated with significantly higher mortality rates. Furthermore, the incidence of postoperative AKI in such cases is more than twice that observed in the control group (18, 19). The new-generation transcatheter heart valves (THVs) are specifically designed with innovative features aimed at reducing procedural complications such as paravalvular leakage, vascular injury, and conduction disturbances, thereby improving clinical outcomes. These advancements are closely associated with a reduced risk of postoperative AKI.

TAVR patients often present with multiple baseline comorbidities, such as diabetes, chronic kidney disease (CKD), and hypertension. Additionally, contrast agents are routinely used during the preoperative and intraoperative phases. The proposed mechanisms of TAVR-related AKI include renal hypoperfusion and acute tubular necrosis, mediated by azotemia and the direct nephrotoxic effects of contrast exposure (20). Reported risk factors for AKI following TAVR vary considerably across studies, with no clear consensus. To address this inconsistency, the present study conducted a systematic review and meta-analysis to identify risk factors associated with post-TAVR AKI. A better understanding and recognition of these factors, along with the implementation of targeted preventive strategies, may help improve survival and clinical outcomes in patients undergoing TAVR.

## 2 Methods and materials

### 2.1 Literature search

Two investigators independently searched PubMed, Embase, the Cochrane Library, and Web of Science for relevant English-language studies published up to February 2025. The search strategy combined Medical Subject Headings (MeSH) and free-text terms, including “Transcatheter Aortic Valve Replacement,” “Transcatheter Aortic Valve Implantation,” “TAVR,” “TAVI,” “Acute Kidney Injury,” “Acute Kidney Injuries,” and “Risk Factors.” Detailed search strategies are provided in Supplementary Table S1. This systematic review protocol was registered in PROSPERO (CRD: 420251047715).

### 2.2 Inclusion and exclusion criteria

*Inclusion* *criteria*

Study design: case–control or cohort.
Topic: AKI following TAVR.
Population: patients with clinically confirmed AS treated by TAVR; those who developed postoperative AKI constituted the exposure group, and those without AKI served as controls.
Outcomes: uni- or multivariable analyses of risk factors. When both analyses were reported in the same article, the multivariable findings were extracted; if only univariable data were available, those results were used.

*Exclusion criteria*

Duplicate publications.
Conference abstracts, reviews, meta-analyses, animal studies, letters, or reports without original data.
Studies with unclear or non-extractable outcome data.
Articles whose full text could not be obtained.

### 2.3 Data extraction

All retrieved articles were imported into EndNote for management. Two reviewers independently screened titles, abstracts, and full texts. Studies with clear eligibility were directly included or excluded; discrepancies were resolved by a third reviewer. Full texts were reviewed for eligibility based on the predefined inclusion and exclusion criteria. Relevant data were extracted and cross-checked to ensure accuracy and consistency. First author, publication year, country, study design, sample size, patient sex and age and results from univariable or multivariable risk factor analyses were extracted.

### 2.4 Quality assessment

The quality of case–control studies was evaluated using the Newcastle–Ottawa Scale (NOS) (21), which assesses three domains: selection of study groups (4 points), comparability between groups (2 points), and exposure or outcome assessment (3 points). The maximum score is 9, with scores ≤4 indicating low quality, 5–6 moderate quality, and ≥7 high quality. Disagreements between the two reviewers were resolved through discussion or consultation with a third reviewer.

### 2.5 Statistical analysis

Stata version 15.0 was performed for statistical analyses. For dichotomous variables, odds ratios (ORs) with 95% confidence intervals (CIs) were calculated. Heterogeneity was assessed using the Q test and the I^2^ statistic. A random-effects model was applied if *I*^2^ >50%; otherwise, a fixed-effects model was used. For analyses with *I*^2^ >50%, sensitivity analysis was conducted by sequentially excluding individual studies to identify potential sources of heterogeneity and assess the robustness of the results. Funnel plots and Egger's test were used to evaluate the publication bias, with a significance threshold of *α* = 0.05. A *p*-value < 0.05 was considered statistically significant.

## 3 Results

### 3.1 Literature Search Process and Results

**Figure 1:**
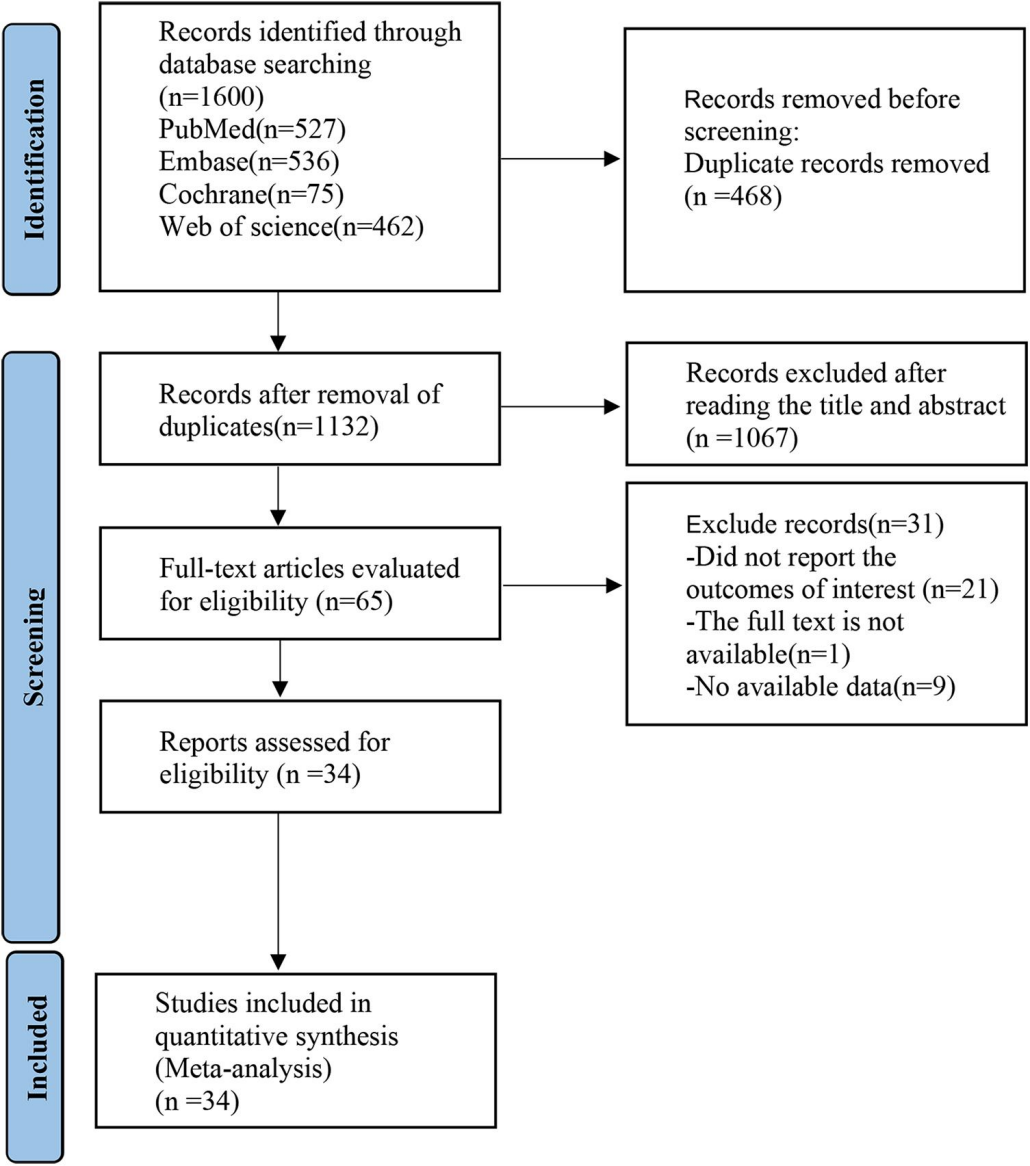
PRISMA flow diagram of the study process. PRISMA, preferred reporting items for systematic review and meta-analysis.

A total of 1,600 records were initially identified from CNKI, PubMed, Embase, the Cochrane Library, and Web of Science. After removing duplicates, 1,132 articles remained. Title and abstract screening yielded 65 potentially eligible studies, and full-text review resulted in the inclusion of 34 studies. Literature selection process is shown in Figure 1.

### 3.2 Basic characteristics of included studies

All 34 included (22–55) studies were case–control in design, comprising a total of 10,353 participants, of whom 2,250 developed AKI after TAVR. The mean age of study populations ranged from 70 to 83 years. Detailed study characteristics are presented in Table 1. For literature quality assessment, one study scored 6 points (31), indicating moderate quality, while the remaining studies scored 7–8 points, reflecting generally high methodological quality (Table 2).

**Table 1:** Study characteristics.

Study	Year	Country	Study design	Sample size	No of AKI	Gender(M/F)	Mean age	Definition of AKI	Regression model
Aregger et al. (22)	2009	Switzerland	cohort study	54	15	20/34	83	RIFLE	logistic regression
Bagur et a. (23)	2010	Canada	cohort study	213	25	99/114	82	RIFLE	logistic regression
Sinning et al. (24)	2010	Germany	cohort study	77	20	37/40	81	AKIN	
Elhmidi et al. (25)	2011	Germany	cohort study	238	46	94/144	81	RIFLE	logistic regression
Nuis et al. (26)	2011	Netherlands	cohort study	126	22	53/73	82	VARC	logistic regression
Khawaj et al. (27)	2012	London	cohort study	248	89	142/106	82	VARC-modified RIFLE	logistic regression
Kong et al. (28)	2012	Australia	cohort study	52	15	33/19	84	RIFLE	logistic regression
Nuis et al. (29)	2012	Netherlands	cohort study	995	206	497/498	82	VARC	logistic regression
Wessely et al. (30)	2012	Germany	cohort study	183	49	82/101	81	RIFLE	logistic regression
Goebel et al. (31)	2013	Germany	cohort study	270	41	120/150	81	VARC	logistic regression
Konigstein et al. (32)	2013	Israel	cohort study	251	42	94/157	83	VARC-2	logistic regression
Saia et al. (33)	2013	Italy	cohort study	102	42	40/62	83	VARC	logistic regression
Yamamoto et al. (34)	2013	France	cohort study	415	63	185/230	83	VARC	logistic regression
Chatani et al. (35)	2015	Germany	cohort study	203	39	88/115	80	VARC-modified RIFLE	logistic regression
Aalaei-Andabili (36)	2016	USA	cohort study	264	65	143/121	80	VARC-I	logistic regression
Crowhurst et al. (38)	2016	Australia	cohort study	209	82	101/108	83	VARC-2modified RIFLE	logistic regression
Arsalan et al. (37)	2016	USA	cohort study	384	144	182/202	82	VARC-2	logistic regression
Thongprayoon et al. (39)	2016	USA	cohort study	386	106	271/115	81	KDIGO	logistic regression
Meneguz-Moreno et al. (40)	2017	Brazil	cohort study	221	52	103/118	82	VARC-2	logistic regression
Türen et al. (41)	2017	Turkey	cohort study	42	14	15/27	77	VARC-2	logistic regression
Attard et al. (42)	2018	Malta	cohort study	103	37	68/35	76	AKIN	logistic regression
Gül et al. (43)	2018	Cyprus	cohort study	133	30	61/72	78	VARC-2	logistic regression
Kandathil et al. (44)	2018	USA	cohort study	106	20	57/49		KDIGO	logistic regression
Shishikura et al. (45)	2018	Australia	cohort study	278	92	161/117		VARC-2	logistic regression
Merchant et al. (46)	2019	USA	cohort study	116	20	64/52		KDIGO	logistic regression
Miura et al. (47)	2019	Japan	cohort study	81	7	22/59		VARC-2	logistic regression
Nunes Filho et al. (48)	2019	Brazil	cohort study	794	143	392/402	81	VARC-2	logistic regression
Chandrasekhar et al. (49)	2021	USA	cohort study	802	139	411/391	82	VARC-1	logistic regression
De Marzo et al. (50)	2023	Italy	cohort study	222	67	95/127	83	VARC-3	logistic regression
Jäckel et al. (51)	2023	Germany	cohort study	366	53	166/200	81	AKIN	logistic regression
Obata et al. (52)	2023	Japan	cohort study	173	22	66/107		VARC-2	logistic regression
Eckrich et al. (53)	2024	Germany	cohort study	1973	347	865/1108	81	VARC-3	logistic regression
Kutsal and Terzi (54)	2024	Turkiye	cohort study	198	83	87/111	78	AKIN	
Xu et al. (55)	2024	China	cohort study	75	13	45/30	70	KDIGO	logistic regression

**Table 2:** Newcastle–Ottawa scale.

Study	Representativeness of the exposed group	Selection of non-exposed groups	Determination of exposure factors	Identification of outcome indicators not yet to be observed at study entry	Comparability of exposed and unexposed groups considered in design and statistical analysis	design and statistical analysis	Adequacy of the study's evaluation of the outcome	Adequacy of follow-up in exposed and unexposed groups	Total scores
Aregger et al. (22) (2009)	*	*	*	*	*	*	*	*	8
Bagur et a. (2010) (23)	*	*	*	*	*	*	*	*	8
Sinning et al. (2010) (24)	*	*	*	*	-	*	*	*	7
Elhmidi et al. (2011) (25)	*	*	*	*	*	*	*	*	8
Nuis et al. (26) (2011)	*	*	*	–	*	*	*	*	7
Khawaj et al. (2012) (27)	*	*	*	*	*	*	*	*	8
Kong et al. (2012) (28)	*	*	*	*	*	*	*	*	8
Nuis et al. (2012) (29)	*	*	*	–	*	*	*	*	7
Wessely et al. (2012) (30)	*	*	*	–	*	*	*	*	7
Goebel et al. (2013) (31)	*	*	*	–	-	*	*	*	6
Konigsteinet al. (2013) (32)	*	*	*	*	*	*	*	*	8
Saia et al. (2013) (33)	*	*	*	*	*	*	*	*	8
Yamamoto et al. (2013) (34)	*	*	*	–	*	*	*	*	7
Chatani et al. (2015) (35)	*	*	*	–	*	*	*	*	7
Aalaei-Andabili et al. (2016) (36)	*	*	*	*	*	*	*	*	8
Crowhurst et al. (2016) (38)	*	*	*	*	*	*	*	*	8
Arsalan et al. (2016) (37)	*	*	*	*	*	*	*	*	8
Thongprayoon et al. (2016) (39)	*	*	*	*	*	*	*	*	8
Meneguz-Moreno et al. (2017) (40)	*	*	*	*	*	*	*	*	8
Türen et al. (2017) (41)	*	*	*	*	*	*	*	*	8
Attard et al. (2018) (42)	*	*	*	*	*	*	*	*	8
Gül et al. (2018) (43)	*	*	*	–	*	*	*	*	7
Kandathil et al. (2018) (44)	*	*	*	–	*	*	*	*	7
Shishikur et al. (2018) (45)	*	*	–	–	*	*	*	*	7
Merchant et al. (2019) (46)	*	*	*	-	*	*	*	*	7
Miura et al. (2019) (47)	*	*	*	–	*	*	*	*	7
Nunes Filho et al. (2019) (48)	*	*	*	*	*	*	*	*	8
Chandrasekhar et al. (2021) (49)	*	*	*	*	*	*	*	*	8
De Marzo et al. (2023) (50)	*	*	*	–	*	*	*	*	7
Jäckel et al. (2023) (51)	*	*	*	*	*	*	*	*	8
Obata et al. (2023) (52)	*	*	*	–	*	*	*	*	7
Eckrich et al. (2024) (53)	*	*	*	–	*	*	*	*	7
Kutsal and Terzi (2024) (54)	*	*	*	–	*	*	*	*	7
Xu et al. (2024) (55)	*	*	*	*	*	*	*	*	8

### 3.3 Univariable meta-analysis

#### 3.3.1 Hypertension

**Figure 2:**
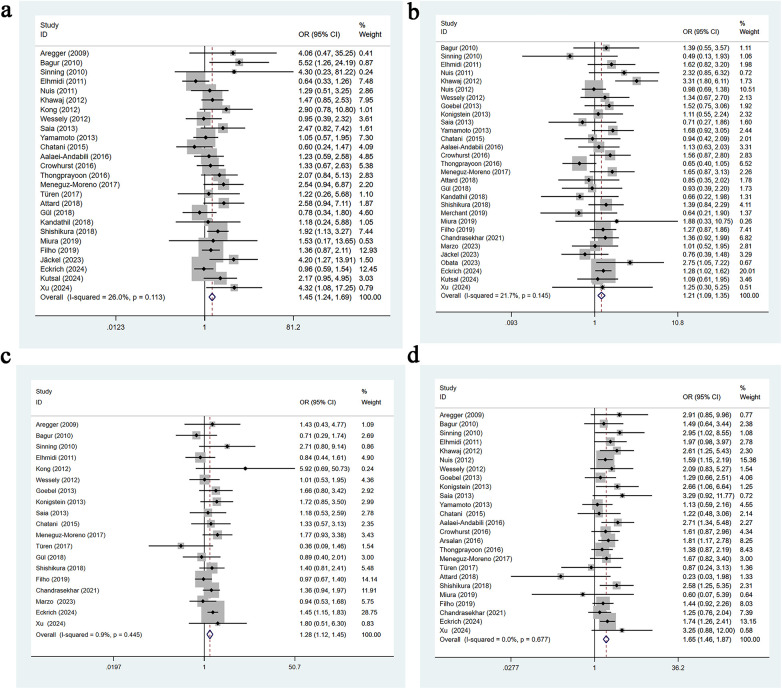
**(a)** Hypertension univariate forest plot; **(b)** diabetes univariate forest plot; **(c)** coronary artery disease univariate forest plot; **(d)** peripheral vascular disease univariate forest plot.

Twenty-six studies reported a history of hypertension. Heterogeneity was low (*I*^2^ = 26%, *p* = 0.113), so a fixed-effects model was used. Univariable analysis indicated that hypertension is a significant risk factor for AKI following TAVR [OR = 1.45, 95% CI: 1.24–1.69], as shown in Figure 2a.

#### 3.3.2 Diabetes

Thirty studies reported a history of diabetes. Heterogeneity was low (*I*^2^ = 21.7%, *p* = 0.145), and a fixed-effects model was applied. Univariable analysis showed that diabetes is a significant risk factor for AKI after TAVR [OR = 1.21, 95% CI: 1.09–1.35], as presented in Figure 2b.

#### 3.3.3 Coronary artery disease

Nineteen studies reported a history of coronary artery disease. Heterogeneity was minimal (*I*^2^ = 0.9%, *p* = 0.445), so a fixed-effects model was used. Univariable analysis demonstrated that coronary artery disease is significantly associated with an increased risk of AKI following TAVR [OR = 1.28, 95% CI: 1.12–1.45], as shown in Figure 2c.

#### 3.3.4 Peripheral vascular disease

Twenty-five studies reported a history of peripheral vascular disease. Heterogeneity was not observed (*I*^2^ = 0.0%, *p* = 0.677), and a fixed-effects model was used. Univariable analysis indicated that peripheral vascular disease is a significant risk factor for AKI following TAVR [OR = 1.65, 95% CI: 1.46–1.87], as shown in Figure 2d.

#### 3.3.5 Porcelain aorta

**Figure 3:**
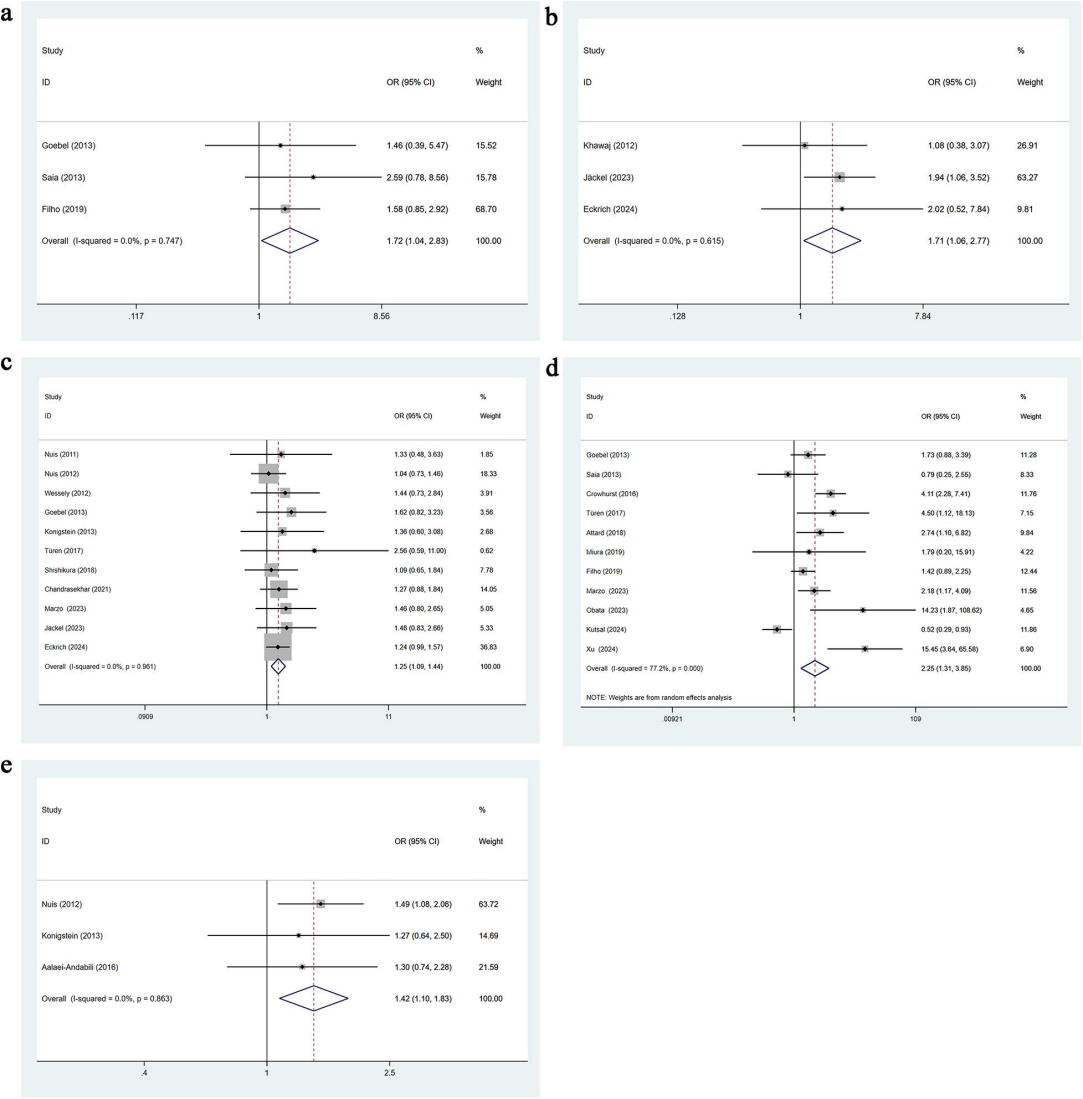
**(a)** Porcelain aorta univariate forest plot; **(b)** PCI for TAVR univariate forest plot; **(c)** atrial fibrillation univariate forest plot; **(d)** CKD univariate forest plot; **(e)** congestive heart failure univariate forest plot.

Three studies reported the presence of a porcelain aorta. No heterogeneity was detected (*I*^2^ = 0.0%, *p* = 0.747), so a fixed-effects model was applied. Univariable analysis showed that porcelain aorta significantly increases the risk of AKI after TAVR [OR = 1.72, 95% CI: 1.04–2.83], as presented in Figure 3a.

#### 3.3.6 Periprocedural percutaneous coronary intervention (PCI for TAVR)

Three studies reported periprocedural PCI during TAVR. No heterogeneity was observed (*I*^2^ = 0.0%, *p* = 0.615), and a fixed-effects model was used. Univariable analysis indicated that PCI for TAVR is a significant risk factor for post-TAVR AKI [OR = 1.71, 95% CI: 1.06–2.77], as shown in Figure 3b.

#### 3.3.7 Atrial fibrillation

Eleven studies reported a history of atrial fibrillation. Heterogeneity was negligible (*I*^2^ = 0.0%, *p* = 0.961), and a fixed-effects model was applied. Univariable analysis showed that atrial fibrillation significantly increases the risk of AKI after TAVR [OR = 1.25, 95% CI: 1.09–1.44], as presented in Figure 3c.

#### 3.3.8 Chronic kidney disease

Eleven studies reported a history of chronic kidney disease. Heterogeneity was substantial (*I*^2^ = 77.2%, *p* = 0.000), so a random-effects model was applied. Univariable analysis indicated that chronic kidney disease is a significant risk factor for AKI following TAVR [OR = 2.25, 95% CI: 1.31–3.85], as shown in Figure 3d.

#### 3.3.9 Congestive heart failure

Three studies reported a history of congestive heart failure. No heterogeneity was observed (*I*^2^ = 0.0%, *p* = 0.863), and a fixed-effects model was used. Univariable analysis showed that congestive heart failure significantly increases the risk of AKI after TAVR [OR = 1.42, 95% CI: 1.10–1.83], as presented in Figure 3e.

#### 3.3.10 NYHA class III–IV

**Figure 4:**
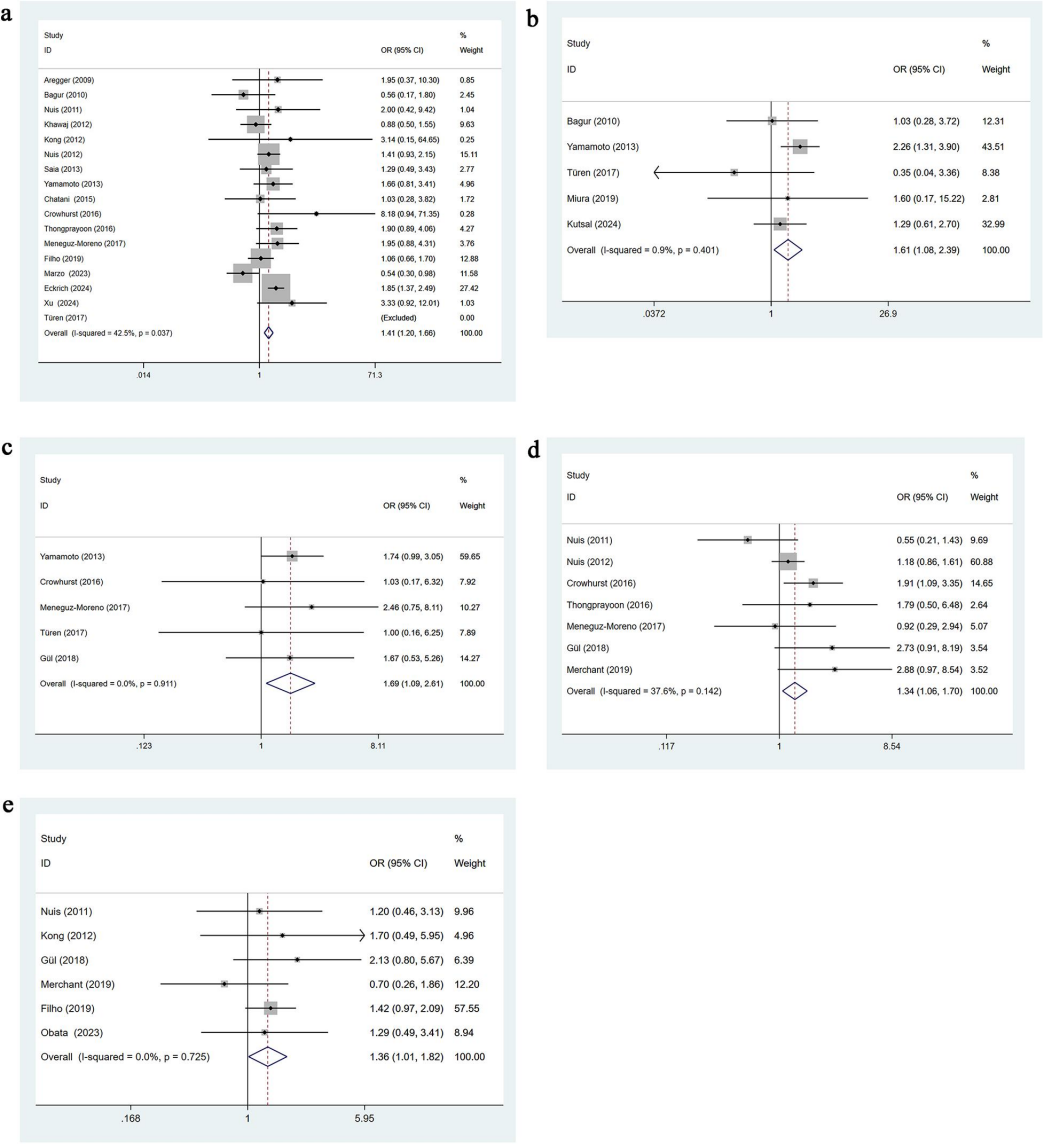
**(a)** NYHA class III–IV univariate forest plot; **(b)** LVEF less than 40% univariate forest plot; **(c)** postoperative aortic regurgitation more than grade 2 univariate forest plot; **(d)** preoperative Anemia univariate forest plot; **(e)** diuretic Use univariate forest plot.

Seventeen studies reported preoperative New York Heart Association (NYHA) functional classification as class III–IV. Moderate heterogeneity was observed (*I*^2^ = 42.5%, *p* = 0.037), and a fixed-effects model was used. Univariable analysis indicated that NYHA class III–IV is a significant risk factor for AKI following TAVR [OR = 1.41, 95% CI: 1.20–1.66], as shown in Figure 4a.

#### 3.3.11 Left ventricular ejection fraction <40%

Five studies reported a left ventricular ejection fraction (LVEF) <40%. Heterogeneity was minimal (*I*^2^ = 0.9%, *p* = 0.401), and a fixed-effects model was applied. Univariable analysis showed that LVEF <40% significantly increases the risk of post-TAVR AKI [OR = 1.61, 95% CI: 1.08–2.39], as presented in Figure 4b.

#### 3.3.12 Postoperative aortic regurgitation >grade 2

Five studies reported postoperative aortic regurgitation greater than grade 2. No heterogeneity was observed (*I*^2^ = 0.0%, *p* = 0.911), and a fixed-effects model was used. Univariable analysis indicated that aortic regurgitation >grade 2 is a significant risk factor for AKI after TAVR [OR = 1.69, 95% CI: 1.19–2.61], as shown in Figure 4c.

#### 3.3.13 Preoperative anemia

Seven studies reported preoperative anemia. Heterogeneity was low (*I*^2^ = 37.6%, *p* = 0.142), and a fixed-effects model was applied. Univariable analysis showed that preoperative anemia significantly increases the risk of post-TAVR AKI [OR = 1.34, 95% CI: 1.06–1.70], as presented in Figure 4d.

#### 3.3.14 Diuretic use

Six studies reported the use of diuretics. No heterogeneity was detected (*I*^2^ = 0.0%, *p* = 0.725), and a fixed-effects model was applied. Univariable analysis indicated that diuretic use is a significant risk factor for AKI after TAVR [OR = 1.36, 95% CI: 1.01–1.82], as shown in Figure 4e.

#### 3.3.15 Transapical access

**Figure 5:**
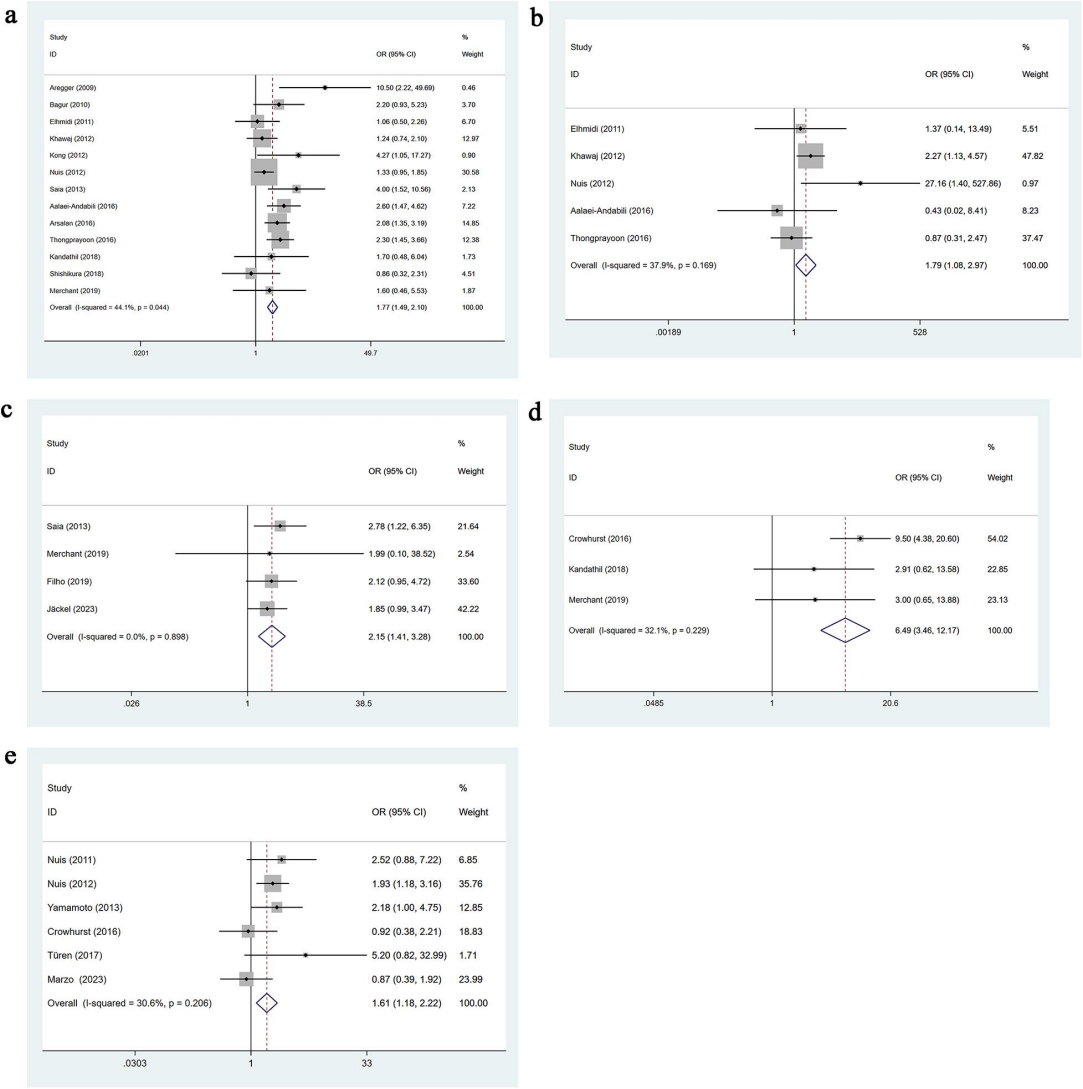
**(a)** Transapical access univariate forest plot; **(b)** transaortic access univariate forest plot; **(c)** general anesthesia univariate forest plot; **(d)** intraoperative rapid pacing univariate forest plot; **(e)** vascular complications univariate forest plot.

Thirteen studies reported the use of transapical access. Moderate heterogeneity was present (*I*^2^ = 44.1%, *p* = 0.044), and a fixed-effects model was used. Univariable analysis showed that transapical access significantly increases the risk of AKI following TAVR [OR = 1.77, 95% CI: 1.49–2.10], as presented in Figure 5a.

#### 3.3.16 Transaortic access

Five studies reported the use of transaortic access. Heterogeneity was low (*I*^2^ = 37.9%, *p* = 0.169), and a fixed-effects model was applied. Univariable analysis indicated that transaortic access is a significant risk factor for AKI after TAVR [OR = 1.79, 95% CI: 1.08–2.97], as shown in Figure 5b.

#### 3.3.17 General anesthesia

Four studies reported the use of general anesthesia. No heterogeneity was observed (*I*^2^ = 0.0%, *p* = 0.898), and a fixed-effects model was used. Univariable analysis demonstrated that general anesthesia significantly increases the risk of AKI following TAVR [OR = 2.15, 95% CI: 1.41–3.28], as presented in Figure 5c.

#### 3.3.18 Intraoperative rapid pacing

Three studies reported intraoperative rapid pacing. Heterogeneity was low (*I*^2^ = 32.1%, *p* = 0.229), and a fixed-effects model was applied. Univariable analysis indicated that rapid pacing is a significant risk factor for AKI following TAVR [OR = 6.49, 95% CI: 3.46–12.17], as shown in Figure 5d.

#### 3.3.19 Vascular complications

Six studies reported vascular complications. Heterogeneity was minimal (*I*^2^ = 30.6%, *p* = 0.206), and a fixed-effects model was used. Univariable analysis demonstrated that vascular complications significantly increase the risk of AKI after TAVR [OR = 1.61, 95% CI: 1.18–2.22], as presented in Figure 5e.

#### 3.3.20 Bleeding complications

**Figure 6:**
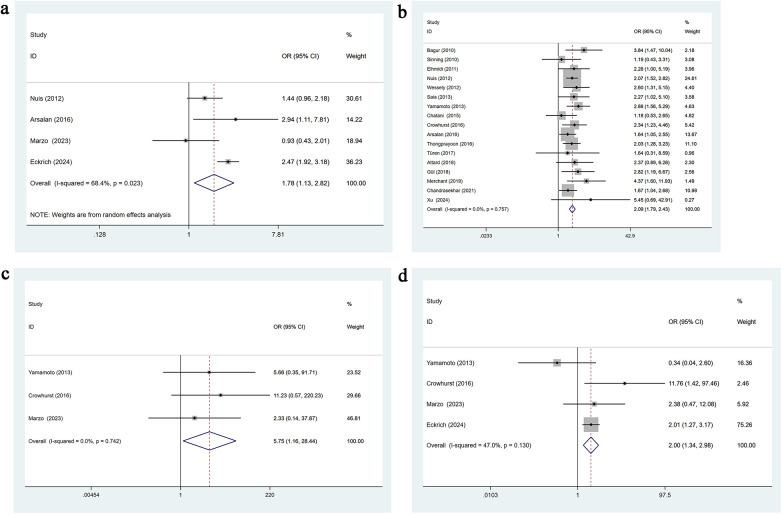
**(a)** Bleeding complications univariate forest plot; **(b)** blood transfusion univariate forest plot; **(c)** postoperative MI univariate forest plot; **(d)** postoperative stroke univariate forest plot.

Four studies reported bleeding complications. Moderate heterogeneity was present (*I*^2^ = 68.4%, *p* = 0.023), and a random-effects model was applied. Univariable analysis indicated that bleeding complications are a significant risk factor for AKI following TAVR [OR = 1.78, 95% CI: 1.13–2.82], as shown in Figure 6a.

#### 3.3.21 Blood transfusion

Seventeen studies reported perioperative blood transfusion. No heterogeneity was observed (*I*^2^ = 0.0%, *p* = 0.757), and a fixed-effects model was used. Univariable analysis showed that transfusion is a significant risk factor for post-TAVR AKI [OR = 2.09, 95% CI: 1.79–2.43], as presented in Figure 6b.

#### 3.3.22 Postoperative myocardial infarction

Three studies reported postoperative myocardial infarction. No heterogeneity was observed (*I*^2^ = 0.0%, *p* = 0.742), and a fixed-effects model was applied. Univariable analysis indicated that postoperative myocardial infarction is a significant risk factor for AKI following TAVR [OR = 5.57, 95% CI: 1.16–28.44], as shown in Figure 6c.

#### 3.3.23 Postoperative stroke

Four studies reported postoperative stroke. Heterogeneity was moderate (*I*^2^ = 47.0%, *p* = 0.130), and a fixed-effects model was used. Univariable analysis showed that postoperative stroke significantly increases the risk of AKI after TAVR [OR = 2.00, 95% CI: 1.34–2.98], as presented in Figure 6d.

### 3.4 Multivariable meta-analysis results

Pooled multivariable analysis showed no significant associations between AKI after TAVR and age, sex, diabetes, logistic EuroSCORE, eGFR, contrast volume, or blood transfusion. In contrast, the following were identified as independent risk factors for post-TAVR AKI: hypertension [OR = 2.87, 95% CI: 1.52–5.42], coronary artery disease [OR = 1.46, 95% CI: 1.16–1.82], peripheral vascular disease [OR = 1.71, 95% CI: 1.38–2.12], history of stroke [OR = 1.61, 95% CI: 1.10–2.35], chronic kidney disease [OR = 3.27, 95% CI: 1.98–5.40], serum creatinine level [OR = 2.80, 95% CI: 2.03–3.86], STS score [OR = 1.06, 95% CI: 1.01–1.11], and transapical access [OR = 3.45, 95% CI: 2.06–5.78]. Detailed results are presented in enclosed material Supplementary Figures S1–S8.

### 3.5 Publication bias

Egger's test and funnel plots were used to assess publication bias for each risk factor. For both univariable and multivariable analyses, all *p*-values were greater than 0.05, indicating no significant publication bias. Detailed results are shown in enclosed material Supplementary Figures S1–S43.

## 3 Discussion

Previous studies have demonstrated that certain demographic variables and comorbidities are associated with an increased risk of AKI following AVR. Both univariable and multivariable meta-analyses in the present study identified hypertension as a significant risk factor for AKI after TAVR. Elevated arterial pressure, if not adequately buffered by afferent arteriolar vasoconstriction, can increase capillary hydrostatic pressure within the glomeruli. This may result in hyaline degeneration and luminal narrowing of arterioles, contributing to glomerular ischemia and sclerosis. Persistently uncontrolled blood pressure can impair the autoregulatory capacity of residual nephrons, allowing systemic pressure to be transmitted directly to the glomeruli. This mechanism underlies hypertensive nephropathy and may lead to progressive chronic renal insufficiency (56).

Current evidence on the association between diabetes and AKI following TAVR remains conflicting. In this study, univariable meta-analysis identified diabetes as a significant risk factor for post-TAVR AKI, whereas multivariable meta-analysis showed no statistically significant association. Thongprayoon et al. (57) identified diabetes as an independent risk factor for persistent AKI (pAKI) following TAVR [OR = 2.43, 95% CI: 1.29–4.66]; Giannini et al. (58) reported that postoperative hyperglycemia is an independent predictor of AKI. Patients with hyperglycemia after TAVR had a twofold higher incidence of AKI compared to those without hyperglycemia (48% vs. 25%, *p* < 0.001). In contrast, Gebauer et al. (59) reported no significant difference in the prevalence of diabetes between patients with and without AKI. A possible mechanism is that diabetic microvascular disease alters renal hemodynamics. Early stages are characterized by glomerular hyperfiltration with elevated pressure and perfusion, while later stages involve progressive decline in glomerular filtration rate and persistent proteinuria. During the perioperative period of TAVR, varying degrees of renal hypoperfusion may occur, contributing to the development of AKI.

Coronary artery disease (CAD) shares common risk factors and pathophysiological mechanisms with degenerative AS and is a frequent comorbidity in patients undergoing TAVR (60, 61). Meta-analysis indicates that approximately half of patients undergoing TAVR have coexisting CAD, often involving multivessel disease (62), current guidelines recommend routine assessment of CAD prior to aortic valve intervention (6), primarily through coronary angiography or coronary CTA.Both univariable and multivariable meta-analyses in this study identified coronary artery disease as a risk factor for AKI following TAVR. Possible mechanisms include shared risk factors with AKI, use of nephrotoxic contrast agents, increased bleeding risk from antiplatelet therapy, and coronary interventions. Similarly, univariable meta-analysis in this study identified periprocedural coronary intervention as a risk factor for AKI following TAVR, though this remains a subject of debate across studies. Revascularization of significant proximal coronary lesions before TAVI is recommended, but the optimal timing of percutaneous coronary intervention (PCI) remains unclear (6, 7). A subgroup analysis of the SURTAVI trial showed that staged PCI, compared to combined TAVR + PCI, was associated with higher contrast volume and a greater risk of AKI (11.8% vs. 2.0%, *p* = 0.04). This may be attributed to the selection of patients with more complex coronary artery disease for staged PCI (63). A meta-analysis of nine studies involving 3,858 patients found no significant difference in the risk of AKI between TAVR with and without PCI [OR = 0.89, 95% CI: 0.42–1.88] (62). Only three studies in this analysis addressed periprocedural PCI, highlighting a limitation in sample size. Additional studies are needed to further investigate whether PCI increases the incidence of AKI after TAVR.

Univariable meta-analysis in this study identified peripheral arterial disease (PAD) and porcelain aorta as significant risk factors for AKI following TAVR. Clinical trials report a PAD prevalence of 27.8%–41.3% among TAVR patients. In the Society of Thoracic Surgeons/American College of Cardiology (STS/ACC) database, up to 50% of patients undergoing non-transfemoral TAVR had coexisting PAD, which was associated with increased risks of vascular complications, major bleeding, stroke, and AKI (64, 65). Reduced renal perfusion caused by systemic atherosclerosis is a key contributor to renal impairment and may trigger AKI. Our multivariable meta-analysis likewise confirmed PAD as an independent risk factor for AKI after TAVR. Earlier studies in cardiac surgery patients have shown that atherosclerosis of the ascending aorta predicts postoperative renal deterioration; invasive manipulation can dislodge aortic plaques, which then embolize to downstream beds, including the renal arteries (66, 67). During valvuloplasty, catheter manipulation across the aorta, and prosthetic valve deployment, dislodged cholesterol emboli may lead to atheroembolic renal artery obstruction, triggering AKI. Increased urinary eosinophils support this pathophysiological mechanism (68, 69).

Atrial fibrillation, one of the most common clinical arrhythmias, frequently occurs in patients with structural heart disease. Both pre-existing and new-onset atrial fibrillation are common in TAVR patients and are associated with increased risks of major bleeding, stroke, and mortality. Several previous meta-analyses have identified atrial fibrillation as a risk factor for AKI following TAVR, consistent with the findings of this study (70, 71). In a retrospective cohort study, Arrotti et al. found that TAVR patients with pre-existing atrial fibrillation had a higher incidence of AKI [OR = 1.65, 95% CI: 1.15–2.38]. Potential mechanisms include reduced cardiac output due to rhythm disturbance, exacerbation of heart failure, bleeding events related to anticoagulation, and thromboembolism (72, 73).

Both univariable and multivariable meta-analyses in this study identified CKD as a significant risk factor for AKI following TAVR. Preoperative renal dysfunction is common among patients requiring AVR, primarily due to chronic hemodynamic alterations from severe AS and low cardiac output, as well as cardiorenal syndrome caused by congestive heart failure. Additional contributing factors include comorbid hypertension and diabetes, which further increase susceptibility to AKI.A study from the French registry reported that in a subgroup analysis of TAVR outcomes across CKD stages, more than half of patients (52.7%) had moderate to severe renal impairment (stage 3a or higher). The incidence of AKI increased progressively with advancing CKD stage and severity (74). A retrospective study by Rahman et al. reported AKI incidence rates of 23.7% in patients with CKD and 14.5% in those without CKD after TAVR (*p* = 0.455) (75), consistent with the findings of our study.

Severe, symptomatic AS with left-ventricular dysfunction is the principal indication for TAVR. Our univariable meta-analysis shows that congestive heart failure, NYHA class III–IV, and a left-ventricular ejection fraction <40% are significant predictors of post-TAVR AKI. AS raises afterload, reduces stroke volume, and elevates left-ventricular pressure, provoking hypertrophy, loss of compliance, and increased myocardial oxygen demand. Concomitant structural changes-left-atrial enlargement, mitral annular calcification or regurgitation, diminished coronary flow reserve, and subendocardial ischemia-further impair ventricular function, progressively lower cardiac output, and manifest clinically as heart failure (76). Heart failure (HF) is a well-established risk factor for AKI. The kidneys play a critical role in the pathophysiology of HF, with multiple organ systems and mechanisms contributing to the development of cardiorenal syndrome. These include hemodynamic alterations, neurohormonal activation, inflammation, oxidative stress, and other incompletely understood pathways (77, 78). Patients with AS are inherently susceptible to AKI, and perioperative stressors associated with TAVR may further exacerbate this risk. Data from the Australian Valve Registry showed that among 2,564 TAVR patients, 163 (6.4%) developed AKI. In multivariable analysis, congestive heart failure was an independent predictor of AKI (aOR = 1.52, *p* = 0.048) (79). Zahid et al. analyzed data from the U.S. National Inpatient Sample (NIS) between 2011 and 2018, including 216,023 TAVR hospitalizations. Among these, 27,871 patients (12.9%) developed AKI. Patients with baseline congestive heart failure had a significantly higher adjusted risk of AKI (OR = 2.03, 95% CI: 1.96–2.10) (80). These findings are consistent with the results of our study.

In this study, univariable meta-analysis identified postoperative aortic regurgitation greater than grade 2 as a risk factor for AKI following TAVR. Paravalvular leak (PVL) is a common complication after TAVR, with an overall incidence of 26%–67%. Most cases are mild and asymptomatic, but a subset of patients may experience severe adverse outcomes (81). Seyedvahid et al. reported that PVL adversely affects coronary perfusion, reducing peak flow velocities in the left anterior descending, circumflex, and right coronary arteries by 21.73%, 21.43%, and 21.43%, respectively. PVL may also increase left ventricular workload and promote atherosclerosis. Patients with moderate to severe PVL are at risk of hemodynamic deterioration, left ventricular remodeling, heart failure, and mechanical hemolytic anemia (82), all of which may contribute to the development of AKI after TAVR. Severe aortic root calcification, inappropriate prosthesis sizing, and incorrect implantation depth are major contributors to PVL. Accurate preoperative assessment of aortic root anatomy, proper valve selection, and intraoperative identification and grading of PVL by experienced operators are essential to minimize its incidence and severity. To assess surgical risk and in-hospital mortality, the European System for Cardiac Operative Risk Evaluation (Euro SCORE) and the Society of Thoracic Surgeons (STS) risk score are the most widely used preoperative risk prediction models in cardiac surgery. Risk scoring systems for adverse outcomes after AVR have limitations; however, STS scoring has been shown to outperform Euro SCORE (83). In this study, multivariable meta-analysis identified the STS score as a significant risk factor for AKI following TAVR. Current guidelines for valvular heart disease management recommend an integrated approach combining risk scores with clinical judgment by a multidisciplinary heart team. The STS score is endorsed as a key component of pre-TAVR assessment, incorporating demographics, laboratory data (including serum creatinine and hematocrit), preoperative medications, comorbidities, cardiopulmonary function, coronary artery disease, valvular pathology, and arrhythmias (84).

Across studies, 45%–64% of patients undergoing TAVR for AS present with preoperative anemia, with nearly one-fifth classified as having severe anemia. The prevalence of anemia increases with age. Baseline anemia and lower hemoglobin levels may be associated with higher early and midterm mortality following TAVR (85, 86). The primary causes of anemia include iron deficiency (most common), chronic kidney disease, inflammatory disorders, vitamin B12 or folate deficiency, and myelodysplastic syndromes. Nearly 90% of these causes are potentially reversible before surgery, yet most patients remain undertreated (87). Anemia can contribute to kidney injury by reducing renal oxygen delivery (hypoxic damage), increasing oxidative stress, and impairing hemostasis. Anemia-related platelet dysfunction may elevate bleeding risk in patients undergoing AVR, often necessitating transfusion, which is an additional risk factor for AKI (88). In this study, univariable meta-analysis identified preoperative anemia as a risk factor for AKI following TAVR. In a retrospective single-center observational study, Kutsal et al. found that patients who developed AKI after TAVR had significantly lower preoperative hemoglobin levels (89). Previous meta-analyses have indicated that patients with preoperative anemia have a higher incidence of AKI following TAVR [OR = 1.74, 95% CI: 1.45–2.10]. Baseline anemia also increases the likelihood of transfusion. These findings are consistent with our results (90). In this study, univariable meta-analysis identified blood transfusion as a risk factor for AKI following TAVR. A nationwide Finnish valve registry study including 2,130 patients who underwent TAVR between 2008 and 2017 reported a preoperative anemia rate of 46%, with 293 patients (16.1%) receiving transfusions post-TAVR. In a propensity score–matched analysis of 281 patient pairs, those who received transfusions had a significantly higher risk of AKI compared to those who did not (17.0% vs. 4.4%, *p* < 0.0001). Although transfusion theoretically improves tissue oxygenation by increasing haemoglobin concentration and oxygen-carrying capacity, multiple studies link it to higher mortality in cardiac patients. Proposed mechanisms include depletion of 2,3-diphosphoglycerate in stored blood, which raises haemoglobin's oxygen affinity and limits tissue release; reduced deformability of stored erythrocytes and diminished nitric-oxide delivery; and pro-coagulant factors that promote microvascular obstruction. These changes further compromise tissue oxygenation and may precipitate AKI. Restrictive transfusion strategies are essential. Several measures can reduce the need for postoperative transfusion, including prevention of vascular complications, minimizing the intensity of antithrombotic therapy, and correcting anemia preoperatively with iron supplementation or erythropoiesis-stimulating agents (91). Multivariable meta-analysis in this study showed no statistically significant association between transfusion and AKI after TAVR. Further basic research is needed to clarify whether a relationship exists and to explore the underlying mechanisms.

Few studies have specifically examined the association between diuretic use and AKI following TAVR. In this study, univariable meta-analysis identified diuretic use as a risk factor for post-TAVR AKI. Diuretics are a cornerstone of heart failure management, and their use-often in combination with RAS inhibitors-is common in AS patients with hypertension. Drug-induced nephrotoxicity, volume depletion, and hemodynamic instability are well-recognized contributors to AKI, particularly in patients with underlying CKD (92).

Transfemoral (TF) access is the preferred route for TAVR. In patients with severe peripheral vascular disease, alternative approaches include transaortic (TAo), transapical (TA), subclavian (TSub), transcarotid (TCa), and caval-aortic (TC-Ao) access. Both univariable and multivariable meta-analyses in this study identified TA access as a risk factor for AKI following TAVR. In a study by Thongprayoon et al., 171 of 366 patients with severe AS (47%) underwent TAVR via TA access, with a significantly higher AKI incidence compared to the TF group (38% vs. 18%, *p* < 0.01). TA access remained independently associated with increased AKI risk after logistic regression analysis [OR = 3.20, 95% CI: 1.68–4.36] and propensity score matching (93), similar findings were reported by Biancari et al. following 1:1 propensity score–matched analysis of observational data (94). Possible mechanisms include the routine use of general anesthesia for TA access, which increases the risk of hypotensive episodes and may contribute to AKI. Surgical trauma may trigger inflammatory responses that impair renal function. Patients undergoing TA-TAVR often have more advanced peripheral vascular disease, further increasing their vulnerability to AKI (95). Univariable meta-analysis in this study also identified TAo access as a risk factor for AKI following TAVR. The ROUTE registry, which included 301 high-risk patients undergoing TAo-TAVR, reported an AKI incidence of 9.5% (96). Transapical and transaortic TAVR were once common but are now rarely used. Current expert consensus recommends prioritizing extrathoracic over intrathoracic access when transfemoral access is not feasible, with preference given to transcarotid and transcaval approaches (97).

The first TAVR procedure, performed in 2002, was conducted under mild sedation. General anesthesia (GA) later became the standard approach, particularly in high-risk patients (98). GA provides hemodynamic stability and allows precise valve deployment and positioning under transesophageal echocardiography (TEE) guidance, reducing the risk of paravalvular leak and valve malposition. It also facilitates rapid conversion to open surgery in emergencies. However, it is associated with increased reliance on mechanical ventilation and greater need for catecholamines and vasopressor. TAVR performed under local anesthesia (LA) and/or conscious sedation is associated with shorter procedural and hospital stays, and reduced postoperative use of inotropic agents. A meta-analysis of 40 studies involving 14,388 patients found no significant difference in AKI incidence between the LA-TAVR and GA-TAVR groups (99). Liang et al. reported in a retrospective study that the incidence of AKI was higher with GA) than with local anesthesia/conscious sedation (28.8% vs. 14.0%, *p* = 0.018). Multivariable analysis confirmed GA as an independent predictor of AKI (OR = 2.517, 95% CI 1.013–6.250, *p* = 0.047). Our own univariable meta-analysis similarly identified GA as a risk factor for post-TAVR AKI, although the number of eligible studies was limited. General anesthetics depress cardiovascular and respiratory function and often exert vasodilatory effects, which can provoke intraoperative hypotension and bleeding; subsequent vasopressor use may cause renal and cerebral ischemia (100, 101), all of which may contribute to AKI. Current evidence therefore supports the use of local anesthesia and/or conscious sedation for TAVR where feasible, with careful consideration of patient-specific risk factors and multidisciplinary team evaluation.

Previous studies have reported conflicting findings regarding the association between contrast volume and TAVR-related AKI. CM may induce renal injury, and patients undergoing TAVR are often exposed to large volumes of CM within a short perioperative timeframe. Sudarsky et al. reported that neither the volume of CM used during the TAVI procedure nor the total CM exposure within 7 or 30 days prior to TAVI was associated with AKI (102). Some studies suggest that baseline renal function must be considered when evaluating the nephrotoxic impact of CM volume on remaining functional nephrons. In this study, multivariable meta-analysis found no association between contrast volume and AKI after TAVR. According to Giannini et al., patients with impaired renal function should receive lower CM volumes. While their analysis showed no direct association between absolute CM volume and post-TAVR AKI, standardizing CM volume to baseline eGFR (CM volume/eGFR) revealed significant associations with both increased AKI incidence and mortality (103, 104). In a single-center retrospective study, Reccia et al. found that contrast volume adjusted for body weight [OR = 1.34, 95% CI: 1.07–1.67] was independently associated with AKI. These findings support minimizing contrast use whenever possible, particularly in patients with elevated baseline creatinine levels (105).

Intraoperative cardiac pacing during TAVR is essential, both for rapid pacing during transcatheter valve deployment, balloon valvuloplasty, or pre-/post-dilation to prevent cardiac arrest, and as backup pacing in cases of high-grade conduction block. Traditionally, right ventricular pacing (RVP) is achieved by introducing a temporary pacing lead via the jugular or femoral vein. This approach carries access-related risks, including bleeding, vascular complications, thrombosis, and infection, as well as the potential for right ventricular perforation and cardiac tamponade. It may also reduce cardiac output and contribute to significant intraoperative hypotension (106). In this study, univariable meta-analysis identified intraoperative rapid pacing as a risk factor for AKI following TAVR. Fefer et al. (107) reported that one or two episodes of RVP during the procedure appear relatively safe, whereas three or more episodes were associated with an increased risk of perioperative AKI, with incidence rising from 18% to 28%. The increased risk of AKI may be attributed to a higher incidence of intraoperative hypotension, with additional mechanisms potentially related to myocardial injury. With the advancement of minimalist TAVR techniques, left ventricular wire pacing has emerged as a viable alternative for rapid pacing, offering advantages in safety, efficacy, and potentially reducing procedural time and cost (108). Given the association between RVP, AKI, and stroke, its use should be minimized whenever possible.

In this study, univariable meta-analysis identified vascular and bleeding complications as risk factors for AKI following TAVR. Postprocedural bleeding is often related to vascular complications (109). Vascular complications include access site bleeding and hematoma, vascular perforation with or without retroperitoneal hemorrhage, iliac artery dissection, aortic dissection or rupture, pseudoaneurysm, arterial stenosis or thrombosis, and arterial occlusion. These events are associated with poor outcomes, renal impairment, and access site infections. Early TAVR procedures were associated with vascular complication rates as high as 30%. Recent studies have reported a significant decline in incidence (110, 111). A multicenter retrospective study involving 1,573 patients reported an overall vascular complication rate of 11%, with 5% classified as minor and 6% as major. The incidence of severe or life-threatening bleeding was 5.9% (112). Given the potential for serious, life-threatening outcomes, operators must be well-versed in recognizing and managing these complications.

AMI after TAVR is relatively rare but carries a high risk, particularly in the case of ST-elevation myocardial infarction (STEMI). In a multicenter international study involving 42,252 TAVR patients from 38 centers, 118 developed STEMI post-procedure, with a cumulative incidence of 0.3%. Other reports have documented post-TAVR AMI incidence rates ranging from 0.2% to 1.25%.

The most common causes are mechanical obstruction of the coronary ostia and procedure-related coronary embolism. Other reported embolic causes of AMI include prosthetic valve endocarditis and bioprosthetic valve thrombosis (113–116). In this study, univariable meta-analysis identified postoperative myocardial infarction as a risk factor for AKI following TAVR. However, related mechanisms and supporting evidence are limited. A large national retrospective study in the United States found no significant difference in coronary revascularization rates-including coronary angiography, PCI, or CABG-between TAVR and non-TAVR patients (117). In patients with STEMI following TAVR, emergency PCI is associated with longer procedure and fluoroscopy times, higher contrast volume (all *p* < 0.01), and a higher rate of PCI failure (16.5% vs. 3.9%; *p* < 0.001). Additionally, cardiac MRI studies have shown that 18% of post-TAVR patients exhibit ischemic injury patterns, likely related to coronary embolism and significantly reduced left ventricular function (115, 116). These factors may contribute to the increased risk of AKI following myocardial infarction after TAVR.

Few studies in this review addressed the impact of postprocedural stroke or prior stroke history on AKI after TAVR. Univariate meta-analysis identified postprocedural stroke as a risk factor for AKI, while multivariate analysis indicated that a history of stroke was also associated with increased AKI risk. However, relevant clinical data are limited, and the underlying mechanisms remain unclear. Studies have shown that 3% to 7% of patients experience major stroke within 30 days after TAVR, most of which are ischemic. Over half of these events are procedure-related, resulting from embolic debris entering cerebral circulation. Embolic material may include thrombi, arterial wall fragments, valve tissue, calcifications, and foreign bodies from catheters or delivery systems (118–120). This aligns with findings that anticoagulation therapy does not significantly reduce post-TAVR stroke incidence (121), suggesting a potential mechanism contributing to postprocedural AKI.

Data from the U.S. Nationwide Inpatient Sample (NIS) show that between 2011 and 2014, 10,114 patients (24%) underwent non-elective TAVR. In the propensity score analysis, elective TAVR was associated with a lower incidence of complications, including AKI, hemodialysis and major bleeding. The above conclusions are further corroborated by a related meta-analysis (17). Ktenopoulos et al. conducted a meta-analysis of 7 studies involving 71,909 patients, among whom 5,108 underwent urgent TAVR. It was associated with an increased risk of AKI (RR: 2.20; 95% CI: 1.53–3.16), which may correlate with higher mortality rates. Patients undergoing urgent TAVR present with a greater burden of baseline comorbidities, such as cardiogenic shock and decompensated heart failure, leading to inherently insufficient renal perfusion. Concurrently, the increased incidence of procedure-related vascular complications further contributes to the elevated risk of AKI. In urgent settings, employing a simplified strategy—using local anesthesia or conscious sedation and streamlining the transfemoral approach—avoids unnecessary invasive procedures. This approach can help mitigate perioperative risks, including AKI, in patients with hemodynamic instability (122).

Since 2020, several trials comparing contemporary TAVR devices have been conducted. A recent meta-analysis encompassing 16 studies and 10,174 patients demonstrated that balloon-expandable valves (BEVs), compared to self-expanding valves (SEVs), reduce the risks of stroke, permanent pacemaker implantation (PPI), and moderate-or-greater paravalvular leak (PVL), but increase the risk of patient-prosthesis mismatch (123).The Myval THV, a novel balloon-expandable THV, features a unique design with incremental diameter differences of only 1.5 mm between consecutive standard sizes, allowing for a more precise and tailored fit to the aortic annulus. Its safety and efficacy have been demonstrated. Relevant meta-analyses suggest that Myval may offer advantages in specific endpoints, such as procedural success rate, PPI rate, incidence of minor vascular complications, and incidence of moderate-or-greater aortic regurgitation (AR)—all of which have the potential to influence renal outcomes (124).

This study has several limitations. First, patients undergoing TAVR are exposed to large doses of contrast agents within a relatively short period during perioperative evaluation and treatment. In the literature included in this study, there is limited mention of the types of contrast agents used during the perioperative period. Second, although a large number of studies were identified, most were retrospective case-control studies with variable follow-up durations, which may introduce bias. No low-quality studies were included, and sensitivity analyses showed consistent results, suggesting acceptable representativeness. But some variables were defined differently across studies, making standardization difficult and potentially affecting the reliability of the pooled analysis. It must be acknowledged that the definitions of AKI varied across the studies included in this analysis. When different definitions are applied, approximately 10% of TAVR patients may be reclassified into different stages of AKI severity. Nevertheless, existing studies have confirmed that regardless of whether the KDIGO/VARC or RIFLE criteria are used, AKI is relatively common among TAVR patients and is associated with poorer long-term outcomes. Finally, the large number of univariate analyses in the article would increase the risk of overinterpretation and introduce multiplicity bias (125). AND a secondary analysis, the validity of the meta-analysis depends on the quality of the original studies.

## 5 Conclusion

Current evidence indicates that the following factors increase the risk of acute kidney injury after AVR: hypertension, diabetes, coronary artery disease, peripheral vascular disease, porcelain aorta, periprocedural coronary intervention, atrial fibrillation, chronic kidney disease, congestive heart failure, NYHA class III–IV, left-ventricular ejection fraction <40%, postoperative aortic regurgitation >grade 2, elevated STS score, preoperative anemia, diuretic use, transapical access, transaortic access, general anesthesia, intraoperative rapid pacing, vascular complications, bleeding complications, blood transfusion, postoperative myocardial infarction, postoperative stroke, and prior stroke. GRADE evaluation are shown in enclosed material Supplementary Table S2, central illustration shown in enclosed material Supplementary Figure S44. Early recognition, diagnosis, and intervention based on these predictors may improve patient outcomes. Given the limited number and variable quality of available studies, further high-quality research is required to confirm these findings.

## Data Availability

The original contributions presented in the study are included in the article/Supplementary Material, further inquiries can be directed to the corresponding author.

## References

[1] Tsao CW Aday AW Almarzooq ZI Anderson CAM Arora P Avery CL Heart disease and stroke statistics-2023 update: a report from the American Heart Association. Circulation. (2023) 147(8):e93–e621. 10.1161/cir.0000000000001123 36695182 PMC12135016

[2] Généreux P Schwartz A Oldemeyer JB Pibarot P Cohen DJ Blanke P Transcatheter aortic-valve replacement for asymptomatic severe aortic stenosis. N Engl J Med. (2025) 392(3):217–27. 10.1056/NEJMoa2405880 39466903

[3] Leon MB Smith CR Mack M Miller DC Moses JW Svensson LG Transcatheter aortic-valve implantation for aortic stenosis in patients who cannot undergo surgery. N Engl J Med. (2010) 363(17):1597–607. 10.1056/NEJMoa1008232 20961243

[4] Leon MB Smith CR Mack MJ Makkar RR Svensson LG Kodali SK Transcatheter or surgical aortic-valve replacement in intermediate-risk patients. N Engl J Med. (2016) 374(17):1609–20. 10.1056/NEJMoa1514616 27040324

[5] Reardon MJ Van Mieghem NM Popma JJ Kleiman NS Søndergaard L Mumtaz M Surgical or transcatheter aortic-valve replacement in intermediate-risk patients. N Engl J Med. (2017) 376(14):1321–31. 10.1056/NEJMoa1700456 28304219

[6] Heidenreich PA Bozkurt B Aguilar D Allen LA Byun JJ Colvin MM 2022 AHA/ACC/HFSA guideline for the management of heart failure: a report of the American College of Cardiology/American Heart Association joint committee on clinical practice guidelines. Circulation. (2022) 145(18):e895–1032. 10.1161/cir.0000000000001063 35363499

[7] Vahanian A Beyersdorf F Praz F Milojevic M Baldus S Bauersachs J 2021 ESC/EACTS guidelines for the management of valvular heart disease. Eur Heart J. (2022) 43(7):561–632. 10.1093/eurheartj/ehab395 34453165

[8] Mack MJ Leon MB Thourani VH Makkar R Kodali SK Russo M Transcatheter aortic-valve replacement with a balloon-expandable valve in low-risk patients. N Engl J Med. (2019) 380(18):1695–705. 10.1056/NEJMoa1814052 30883058

[9] Thyregod HGH Jørgensen TH Ihlemann N Steinbrüchel DA Nissen H Kjeldsen BJ Transcatheter or surgical aortic valve implantation: 10-year outcomes of the notion trial. Eur Heart J. (2024) 45(13):1116–24. 10.1093/eurheartj/ehae043 38321820 PMC10984572

[10] Généreux P Piazza N Alu MC Nazif T Hahn RT Pibarot P Valve academic research consortium 3: updated endpoint definitions for aortic valve clinical research. Eur Heart J. (2021) 42(19):1825–57. 10.1093/eurheartj/ehaa799 33871579

[11] Zhang S Kolominsky-Rabas PL. How tavi registries report clinical outcomes-a systematic review of endpoints based on varc-2 definitions. PLoS One. (2017) 12(9):e0180815. 10.1371/journal.pone.0180815 28910289 PMC5598923

[12] Julien HM Stebbins A Vemulapalli S Nathan AS Eneanya ND Groeneveld P Incidence, predictors, and outcomes of acute kidney injury in patients undergoing transcatheter aortic valve replacement: insights from the society of thoracic surgeons/American College of Cardiology national cardiovascular data registry-transcatheter valve therapy registry. Circ Cardiovasc Interv. (2021) 14(4):e010032. 10.1161/circinterventions.120.010032 33877860

[13] Tay E Khaing T Yin WH Posas EF Kao PH Buddhari W Asia Pacific Tavi registry (an APSIC initiative): initial report of early outcomes: Asia Pacific Tavi registry. AsiaIntervention. (2021) 7(1):54–9. 10.4244/AIJ-D-18-00053 34913003 PMC8657041

[14] Crimi G De Marzo V De Marco F Conrotto F Oreglia J D’Ascenzo F Acute kidney injury after transcatheter aortic valve replacement mediates the effect of chronic kidney disease. J Am Heart Assoc. (2022) 11(19):e024589. 10.1161/jaha.121.024589 36172945 PMC9673702

[15] Elhmidi Y Bleiziffer S Deutsch MA Krane M Mazzitelli D Lange R Acute kidney injury after transcatheter aortic valve implantation: incidence, predictors and impact on mortality. Arch Cardiovasc Dis. (2014) 107(2):133–9. 10.1016/j.acvd.2014.01.002 24556191

[16] Moriyama N Laakso T Raivio P Dahlbacka S Kinnunen EM Juvonen T Acute kidney injury following aortic valve replacement in patients without chronic kidney disease. Can J Cardiol. (2021) 37(1):37–46. 10.1016/j.cjca.2020.03.015 32535038

[17] Elbadawi A Elgendy IY Mentias A Saad M Mohamed AH Choudhry MW Outcomes of urgent versus nonurgent transcatheter aortic valve replacement. Catheter Cardiovasc Interv. (2020) 96(1):189–95. 10.1002/ccd.28563 31647180

[18] Hoshi T Ieda M. Urgent transcatheter aortic valve replacement for acutely decompensated severe aortic stenosis. Intern Med. (2023) 62(17):2449–50. 10.2169/internalmedicine.1416-22 36725033 PMC10518540

[19] Ktenopoulos N Apostolos A Chlorogiannis DD Kachrimanidis I Vlachakis P Sagris M Conversion to cardiac surgery during elective and urgent transcatheter aortic valve implantation: a systematic review and meta-analysis. Catheter Cardiovasc Interv. (2026) 107(1):210–20. 10.1002/ccd.70309 41214437 PMC12775193

[20] Scherner M Wahlers T. Acute kidney injury after transcatheter aortic valve implantation. J Thorac Dis. (2015) 7(9):1527–35. 10.3978/j.issn.2072-1439.2015.06.14 26543598 PMC4598516

[21] Stang A. Critical evaluation of the Newcastle-Ottawa scale for the assessment of the quality of nonrandomized studies in meta-analyses. Eur J Epidemiol. (2010) 25(9):603–5. 10.1007/s10654-010-9491-z 20652370

[22] Aregger F Wenaweser P Hellige GJ Kadner A Carrel T Windecker S Risk of acute kidney injury in patients with severe aortic valve stenosis undergoing transcatheter valve replacement. Nephrol Dial Transplant. (2009) 24(7):2175–9. 10.1093/ndt/gfp036 19211648

[23] Bagur R Webb JG Nietlispach F Dumont E De Larochellière R Doyle D Acute kidney injury following transcatheter aortic valve implantation: predictive factors, prognostic value, and comparison with surgical aortic valve replacement. Eur Heart J. (2010) 31(7):865–74. 10.1093/eurheartj/ehp552 20037180 PMC2848323

[24] Sinning JM Ghanem A Steinhäuser H Adenauer V Hammerstingl C Nickenig G Renal function as predictor of mortality in patients after percutaneous transcatheter aortic valve implantation. JACC Cardiovasc Interv. (2010) 3(11):1141–9. 10.1016/j.jcin.2010.09.009 21087750

[25] Elhmidi Y Bleiziffer S Piazza N Hutter A Opitz A Hettich I Incidence and predictors of acute kidney injury in patients undergoing transcatheter aortic valve implantation. Am Heart J. (2011) 161(4):735–9. 10.1016/j.ahj.2011.01.009 21473973

[26] Nuis RJ Van Mieghem NM Tzikas A Piazza N Otten AM Cheng J Frequency, determinants, and prognostic effects of acute kidney injury and red blood cell transfusion in patients undergoing transcatheter aortic valve implantation. Catheter Cardiovasc Interv. (2011) 77(6):881–9. 10.1002/ccd.22874 21061244

[27] Khawaja MZ Thomas M Joshi A Asrress KN Wilson K Bolter K The effects of VARC-defined acute kidney injury after transcatheter aortic valve implantation Tavi) using the Edwards bioprosthesis. EuroIntervention. (2012) 8(5):563–70. 10.4244/eijv8i5a87 22995082

[28] Kong WY Yong G Irish A. Incidence, risk factors and prognosis of acute kidney injury after transcatheter aortic valve implantation. Nephrology (Carlton). (2012) 17(5):445–51. 10.1111/j.1440-1797.2012.01593.x 22390156

[29] Nuis RJ Rodés-Cabau J Sinning JM van Garsse L Kefer J Bosmans J Blood transfusion and the risk of acute kidney injury after transcatheter aortic valve implantation. Circ Cardiovasc Interv. (2012) 5(5):680–8. 10.1161/circinterventions.112.971291 23048055

[30] Wessely M Rau S Lange P Kehl K Renz V Schönermarck U Chronic kidney disease is not associated with a higher risk for mortality or acute kidney injury in transcatheter aortic valve implantation. Nephrol Dial Transplant. (2012) 27(9):3502–8. 10.1093/ndt/gfs102 22535634

[31] Goebel N Baumbach H Ahad S Voehringer M Hill S Albert M Transcatheter aortic valve replacement: does kidney function affect outcome? Ann Thorac Surg. (2013) 96(2):507–12. 10.1016/j.athoracsur.2013.04.035 23773729

[32] Konigstein M Ben-Assa E Abramowitz Y Steinvil A Leshem Rubinow E Havakuk O Usefulness of updated valve academic research consortium-2 criteria for acute kidney injury following transcatheter aortic valve implantation. Am J Cardiol. (2013) 112(11):1807–11. 10.1016/j.amjcard.2013.07.048 24012024

[33] Saia F Ciuca C Taglieri N Marrozzini C Savini C Bordoni B Acute kidney injury following transcatheter aortic valve implantation: incidence, predictors and clinical outcome. Int J Cardiol. (2013) 168(2):1034–40. 10.1016/j.ijcard.2012.10.029 23164594

[34] Yamamoto M Hayashida K Mouillet G Chevalier B Meguro K Watanabe Y Renal function-based contrast dosing predicts acute kidney injury following transcatheter aortic valve implantation. JACC Cardiovasc Interv. (2013) 6(5):479–86. 10.1016/j.jcin.2013.02.007 23702012

[35] Chatani K Abdel-Wahab M Wübken-Kleinfeld N Gordian K Pötzing K Mostafa AE Acute kidney injury after transcatheter aortic valve implantation: impact of contrast agents, predictive factors, and prognostic importance in 203 patients with long-term follow-up. J Cardiol. (2015) 66(6):514–9. 10.1016/j.jjcc.2015.02.007 25801148

[36] Aalaei-Andabili SH Pourafshar N Bavry AA Klodell CT Anderson RD Karimi A Acute kidney injury after transcatheter aortic valve replacement. J Card Surg. (2016) 31(7):416–22. 10.1111/jocs.12768 27212701

[37] Arsalan M Squiers JJ Farkas R Worley C Herbert M Stewart W Prognostic usefulness of acute kidney injury after transcatheter aortic valve replacement. Am J Cardiol. (2016) 117(8):1327–31. 10.1016/j.amjcard.2016.01.037 26976788

[38] Crowhurst JA Savage M Subban V Incani A Raffel OC Poon K Factors contributing to acute kidney injury and the impact on mortality in patients undergoing transcatheter aortic valve replacement. Heart Lung Circ. (2016) 25(3):282–9. 10.1016/j.hlc.2015.06.832 26672437

[39] Thongprayoon C Cheungpasitporn W Srivali N Kittanamongkolchai W Greason KL Kashani KB. Incidence and risk factors of acute kidney injury following transcatheter aortic valve replacement. Nephrology (Carlton). (2016) 21(12):1041–6. 10.1111/nep.12704 26714182

[40] Meneguz-Moreno RA Ramos AI Siqueira D de Castro A Jatene T Jeronimo AD Prognostic value of renal function in patients with aortic stenosis treated with transcatheter aortic valve replacement. Catheter Cardiovasc Interv. (2017) 89(3):452–9. 10.1002/ccd.26693 27514499

[41] Türen S Yildirim A Satilmisoglu MH Öz K. Incidence, risk factors and prognostic impact of acute kidney injury on 30-day mortality after transcatheter aortic valve implantation. Turk Gogus Kalp Damar Cerrahisi Dergisi-Turk Jo Thorac Cardiovasc Surg. (2017) 25(4):573–9. 10.5606/tgkdc.dergisi.2017.14529

[42] Attard S Buttigieg J Galea S Mintoff M Farrugia E Cassar A. The incidence, predictors, and prognosis of acute kidney injury after transcatheter aortic valve implantation. Clin Nephrol. (2018) 90(6):373–9. 10.5414/cn109544 30369403

[43] Gül I Cerit L Senturk B Zungur M Alkan MB Kemal H The negative effect of mean perfusion pressure on the development of acute kidney injury after transcatheter aortic valve implantation. Braz J Cardiovasc Surg. (2018) 33(6):559–66. 10.21470/1678-9741-2018-0137 30652744 PMC6326433

[44] Kandathil A Abbara S Hanna M Minhajuddin A Wehrmann L Merchant AM Atherosclerosis on ct angiogram predicts acute kidney injury after transcatheter aortic valve replacement. AJR Am J Roentgenol. (2018) 211(3):677–83. 10.2214/ajr.17.19340 30016147

[45] Shishikura D Kataoka Y Pisaniello AD Delacroix S Montarello JK Nicholls SJ The extent of aortic atherosclerosis predicts the occurrence, severity, and recovery of acute kidney injury after transcatheter aortic valve replacement. Circ Cardiovasc Interv. (2018) 11(8):e006367. 10.1161/circinterventions.117.006367 30354779

[46] Merchant AM Neyra JA Minhajuddin A Wehrmann LE Mills RA Gualano SK Packed red blood cell transfusion associates with acute kidney injury after transcatheter aortic valve replacement. BMC Anesthesiol. (2019) 19(1):99. 10.1186/s12871-019-0764-0 31185915 PMC6560735

[47] Miura D Yamada Y Kusaba S Nogami E Yunoki J Sakamoto Y Influence of preoperative Serum creatinine level and intraoperative volume of contrast Medium on the risk of acute kidney injury after transfemoral transcatheter aortic valve implantation: a retrospective observational study. BMC Res Notes. (2019) 12(1):484. 10.1186/s13104-019-4527-2 31383003 PMC6683543

[48] Nunes Filho ACB Katz M Campos CM Carvalho LA Siqueira DA Tumelero RT Impact of acute kidney injury on short- and long-term outcomes after transcatheter aortic valve implantation. Rev Esp Cardiol (Engl Ed). (2019) 72(1):21–9. 10.1016/j.rec.2017.11.024 29358043

[49] Chandrasekhar J Sartori S Mehran R Aquino M Vogel B Asgar AW Incidence, predictors, and outcomes associated with acute kidney injury in patients undergoing transcatheter aortic valve replacement: from the bravo-3 randomized trial. Clin Res Cardiol. (2021) 110(5):649–57. 10.1007/s00392-020-01787-7 33839885

[50] De Marzo V Viglino U Zecchino S Matos JG Piredda E Pigati M Supra-Renal aortic atheroma extent and composition predict acute kidney injury after transcatheter aortic valve replacement: a three-dimensional computed tomography study. Int J Cardiol. (2023) 381:8–15. 10.1016/j.ijcard.2023.03.053 37001646

[51] Jäckel M Keller S Prager EP Staudacher DL Schlett C Zehender M The impact of transcatheter aortic valve implantation planning and procedure on acute and chronic renal failure. Cardiol J. (2023) 30(2):247–55. 10.5603/CJ.a2021.0057 34312832 PMC10129259

[52] Obata Y Kamijo-Ikemori A Shimmi S Inoue S. Clinical usefulness of urinary biomarkers for early prediction of acute kidney injury in patients undergoing transaortic valve implantation. Sci Rep. (2023) 13(1). 10.1038/s41598-023-46015-0 PMC10616062 37903844

[53] Eckrich K Mangner N Erbs S Woitek F Kiefer P Schlotter F Baseline nt-probnp predicts acute kidney injury following transcatheter aortic valve implantation. Cardiovasc Revasc Med. (2024) 66:15–20. 10.1016/j.carrev.2024.03.027 38599917

[54] Kutsal DA Terzi S. Factors associated with acute kidney injury in patients undergoing transcatheter aortic valve implantation: short-term outcomes and impact of right heart failure. North Clin Istanb. (2024) 11(2):133–9. 10.14744/nci.2024.87864 38757106 PMC11095335

[55] Xu Z Zhou X Yang H Chen Q Luo G Cai S Incidence and risk factors of acute kidney injury after transcatheter aortic valve replacement. Clin Nephrol. (2024) 101(6):263–70. 10.5414/cn111269 38497685

[56] Hill GS Heudes D Jacquot C Gauthier E Bariéty J. Morphometric evidence for impairment of renal autoregulation in advanced essential hypertension. Kidney Int. (2006) 69(5):823–31. 10.1038/sj.ki.5000163 16518341

[57] Thongprayoon C Cheungpasitporn W Mao MA Srivali N Kittanamongkolchai W Harrison AM Persistent acute kidney injury following transcatheter aortic valve replacement. J Card Surg. (2017) 32(9):550–5. 10.1111/jocs.13200 28833503

[58] Giannini F Latib A Jabbour RJ Ruparelia N Aurelio A Ancona MB Impact of post-procedural hyperglycemia on acute kidney injury after transcatheter aortic valve implantation. Int J Cardiol. (2016) 221:892–7. 10.1016/j.ijcard.2016.07.029 27434367

[59] Gebauer K Meyborg M Malyar N Kerkhoff G Kaleschke G Baumgartner H Frequency and predictors of acute kidney injury, and its impact on outcome after transcatheter aortic valve implantation. Eur Heart J. (2011) 32:896. 10.1093/eurheartj/ehr32421106579

[60] Hajar R. Risk factors for coronary artery disease: historical perspectives. Heart Views. (2017) 18(3):109–14. 10.4103/heartviews.Heartviews_106_17 29184622 PMC5686931

[61] Yan AT Koh M Chan KK Guo H Alter DA Austin PC Association between cardiovascular risk factors and aortic stenosis: the canheart aortic stenosis study. J Am Coll Cardiol. (2017) 69(12):1523–32. 10.1016/j.jacc.2017.01.025 28335833

[62] Kotronias RA Kwok CS George S Capodanno D Ludman PF Townend JN Transcatheter aortic valve implantation with or without percutaneous coronary artery revascularization strategy: a systematic review and meta-analysis. J Am Heart Assoc. (2017) 6(6). 10.1161/jaha.117.005960 28655733 PMC5669191

[63] Søndergaard L Popma JJ Reardon MJ Van Mieghem NM Deeb GM Kodali S Comparison of a complete percutaneous versus surgical approach to aortic valve replacement and revascularization in patients at intermediate surgical risk: results from the randomized surtavi trial. Circulation. (2019) 140(16):1296–305. 10.1161/circulationaha.118.039564 31476897

[64] Rivera FB Al-Abcha A Ansay MFM Magalong JVU Tang VAS Ona HM Transcatheter aortic valve replacement-associated acute kidney injury: an update. Cardiorenal Med. (2023) 13(1):143–57. 10.1159/000529729 36801854

[65] Mohananey D Villablanca P Gupta T Ranka S Bhatia N Adegbala O Association of peripheral artery disease with in-hospital outcomes after endovascular transcatheter aortic valve replacement. Catheter Cardiovasc Interv. (2019) 94(2):249–55. 10.1002/ccd.28310 31025488 PMC6832693

[66] Katz ES Tunick PA Rusinek H Ribakove G Spencer FC Kronzon I. Protruding aortic atheromas predict stroke in elderly patients undergoing cardiopulmonary bypass: experience with intraoperative transesophageal echocardiography. J Am Coll Cardiol. (1992) 20(1):70–7. 10.1016/0735-1097(92)90139-e 1607541

[67] Stern A Tunick PA Culliford AT Lachmann J Baumann FG Kanchuger MS Protruding aortic arch atheromas: risk of stroke during heart surgery with and without aortic arch endarterectomy. Am Heart J. (1999) 138(4 Pt 1):746–52. 10.1016/S0002-8703(99)70191-2 10502222

[68] Ram P Mezue K Pressman G Rangaswami J. Acute kidney injury post-transcatheter aortic valve replacement. Clin Cardiol. (2017) 40(12):1357–62. 10.1002/clc.22820 29251358 PMC6490570

[69] Thongprayoon C Cheungpasitporn W Srivali N Harrison AM Gunderson TM Kittanamongkolchai W Aki after transcatheter or surgical aortic valve replacement. J Am Soc Nephrol. (2016) 27(6):1854–60. 10.1681/asn.2015050577 26487562 PMC4884118

[70] Wang J Yu W Zhou Y Yang Y Li C Liu N Independent risk factors contributing to acute kidney injury according to updated valve academic research consortium-2 criteria after transcatheter aortic valve implantation: a meta-analysis and meta-regression of 13 studies. J Cardiothorac Vasc Anesth. (2017) 31(3):816–26. 10.1053/j.jvca.2016.12.021 28385646

[71] Nso N Emmanuel K Nassar M Bhangal R Enoru S Iluyomade A Impact of new-onset versus Pre-existing atrial fibrillation on outcomes after transcatheter aortic valve replacement/implantation. Int J Cardiol Heart Vasc. (2022) 38:100910. 10.1016/j.ijcha.2021.100910 35146118 PMC8802123

[72] Tarantini G Mojoli M Urena M Vahanian A. Atrial fibrillation in patients undergoing transcatheter aortic valve implantation: epidemiology, timing, predictors, and outcome. Eur Heart J. (2017) 38(17):1285–93. 10.1093/eurheartj/ehw456 27744287

[73] Arrotti S Sgura FA Leo G Vitolo M Monopoli D Forzati N Atrial fibrillation before and after transcatheter aortic valve implantation: short- and long-term clinical implications. J Cardiovasc Med (Hagerstown). (2024) 25(1):51–9. 10.2459/JCM.0000000000001553 38079281 PMC10720825

[74] Oguri A Yamamoto M Mouillet G Gilard M Laskar M Eltchaninoff H Impact of chronic kidney disease on the outcomes of transcatheter aortic valve implantation: results from the France 2 registry. EuroIntervention. (2015) 10(9):e1–9. 10.4244/eijv10i9a183 25599700

[75] Rahman MS Sharma R Brecker SJD. Transcatheter aortic valve implantation in patients with Pre-existing chronic kidney disease. Int J Cardiol Heart Vasc. (2015) 8:9–18. 10.1016/j.ijcha.2015.04.006 28785672 PMC5497245

[76] Bavishi C Kolte D Gordon PC Abbott JD. Transcatheter aortic valve replacement in patients with severe aortic stenosis and heart failure. Heart Fail Rev. (2018) 23(6):821–9. 10.1007/s10741-018-9726-8 30094532

[77] Boorsma EM Ter Maaten JM Voors AA van Veldhuisen DJ. Renal compression in heart failure: the renal tamponade hypothesis. JACC Heart Fail. (2022) 10(3):175–83. 10.1016/j.jchf.2021.12.005 35241245

[78] Mitsas AC Elzawawi M Mavrogeni S Boekels M Khan A Eldawy M Heart failure and cardiorenal syndrome: a narrative review on pathophysiology, diagnostic and therapeutic regimens-from a cardiologist’s view. J Clin Med. (2022) 11(23). 10.3390/jcm11237041 36498617 PMC9741317

[79] Butala AD Nanayakkara S Navani RV Palmer S Noaman S Haji K Acute kidney injury following transcatheter aortic valve implantation-a contemporary perspective of incidence, predictors, and outcomes. Heart Lung Circ. (2024) 33(3):316–23. 10.1016/j.hlc.2023.11.018 38245395

[80] Zahid S Ullah W Khan MU Salama A Krupica T Khan MZ. Predictors of acute kidney injury after transcatheter aortic valve implantation (from national inpatient sample [2011–2018]). Am J Cardiol. (2021) 151:120–2. 10.1016/j.amjcard.2021.04.003 34006370

[81] Bhushan S Huang X Li Y He S Mao L Hong W Paravalvular leak after transcatheter aortic valve implantation its incidence, diagnosis, clinical implications, prevention, management, and future perspectives: a review article. Curr Probl Cardiol. (2022) 47(10):100957. 10.1016/j.cpcardiol.2021.100957 34364915

[82] Khodaei S Garber L Bauer J Emadi A Keshavarz-Motamed Z. Long-term prognostic impact of paravalvular leakage on coronary artery disease requires patient-specific quantification of hemodynamics. Sci Rep. (2022) 12(1):21357. 10.1038/s41598-022-21104-8 36494362 PMC9734172

[83] Wendt D Osswald BR Kayser K Thielmann M Tossios P Massoudy P Society of thoracic surgeons score is superior to the euroscore determining mortality in high risk patients undergoing isolated aortic valve replacement. Ann Thorac Surg. (2009) 88(2):468–74; discussion 74–5. 10.1016/j.athoracsur.2009.04.059 19632395

[84] Otto CM Nishimura RA Bonow RO Carabello BA Erwin JP 3rd Gentile F 2020 ACC/AHA guideline for the management of patients with valvular heart disease: executive summary: a report of the American College of Cardiology/American Heart Association joint committee on clinical practice guidelines. Circulation. (2021) 143(5):e35–71. 10.1161/cir.0000000000000932 33332149

[85] De Larochellière H Puri R Eikelboom JW Rodés-Cabau J. Blood disorders in patients undergoing transcatheter aortic valve replacement: a review. JACC Cardiovasc Interv. (2019) 12(1):1–11. 10.1016/j.jcin.2018.09.041 30621965

[86] Cammalleri V Muscoli S Versaci F Romeo F. Periprocedural anemia management in severe aortic stenosis patients undergoing transcatheter aortic valve implantation. J Cardiol. (2020) 75(2):117–23. 10.1016/j.jjcc.2019.08.016 31537438

[87] DeLarochellière H Urena M Amat-Santos IJ Ribeiro HB Allende R Laflamme L Effect on outcomes and exercise performance of Anemia in patients with aortic stenosis who underwent transcatheter aortic valve replacement. Am J Cardiol. (2015) 115(4):472–9. 10.1016/j.amjcard.2014.11.033 25549880

[88] Najjar M Salna M George I. Acute kidney injury after aortic valve replacement: incidence, risk factors and outcomes. Expert Rev Cardiovasc Ther. (2015) 13(3):301–16. 10.1586/14779072.2015.1002467 25592763

[89] Kutsal DA Terzi S. Short-term outcomes after transcatheter aortic valve implantation; risk factors for acute kidney injury and impact of right heart failure. Anatol J Cardiol. (2023) 27:S188–S9. 10.14744/nci.2024.87864 PMC11095335 38757106

[90] Jiménez-Xarrié E Asmarats L Roqué-Figuls M Millán X Li CHP Fernández-Peregrina E Impact of baseline Anemia in patients undergoing transcatheter aortic valve replacement: a prognostic systematic review and meta-analysis. J Clin Med. (2023) 12(18). 10.3390/jcm12186025 PMC10531747 37762965

[91] Costa F Cohen MG. Transfusion and mortality after transcatheter aortic valve replacement: association or causation? Circ Cardiovasc Interv. (2020) 13(12):e010225. 10.1161/circinterventions.120.010225 33272032

[92] Banerjee D Ali MA Wang AY Jha V. Acute kidney injury in acute heart failure-when to worry and when not to worry? Nephrol Dial Transplant. (2024) 40(1):10–8. 10.1093/ndt/gfae146 38944413 PMC11879425

[93] Thongprayoon C Cheungpasitporn W Srivali N Harrison AM Kittanamongkolchai W Greason KL Transapical versus transfemoral approach and risk of acute kidney injury following transcatheter aortic valve replacement: a propensity-adjusted analysis. Ren Fail. (2017) 39(1):13–8. 10.1080/0886022x.2016.1244072 27767371 PMC6014512

[94] Biancari F Rosato S D’Errigo P Ranucci M Onorati F Barbanti M Immediate and intermediate outcome after transapical versus transfemoral transcatheter aortic valve replacement. Am J Cardiol. (2016) 117(2):245–51 10.1016/j.amjcard.2015.10.036 26639038

[95] Morcos M Burgdorf C Vukadinivikj A Mahfoud F Latus J Persson PB Kidney injury as post-interventional complication of tavi. Clin Res Cardiol. (2021) 110(3):313–22. 10.1007/s00392-020-01732-8 32844282

[96] Bapat V Frank D Cocchieri R Jagielak D Bonaros N Aiello M Transcatheter aortic valve replacement using transaortic access: experience from the multicenter, multinational, prospective route registry. JACC Cardiovasc Interv. (2016) 9(17):1815–22. 10.1016/j.jcin.2016.06.031 27609256

[97] Sherwood M Allen KB Dahle TG Devireddy CM Gaca J Garcia S Scai expert consensus statement on alternative access for transcatheter aortic valve replacement. J Soc Cardiovasc Angiogr Interv. (2025) 4(3Part A):102514. 10.1016/j.jscai.2024.102514 40231056 PMC11993896

[98] Cribier A Eltchaninoff H Bash A Borenstein N Tron C Bauer F Percutaneous transcatheter implantation of an aortic valve prosthesis for calcific aortic stenosis: first human case description. Circulation. (2002) 106(24):3006–8. 10.1161/01.cir.0000047200.36165.b8 12473543

[99] Ahmed A Mathew DM Mathew SM Awad AK Varghese KS Khaja S General anesthesia versus local anesthesia in patients undergoing transcatheter aortic valve replacement: an updated meta-analysis and systematic review. J Cardiothorac Vasc Anesth. (2023) 37(8):1358–67. 10.1053/j.jvca.2023.03.007 37120319

[100] Ehret C Rossaint R Foldenauer AC Stoppe C Stevanovic A Dohms K Is local anaesthesia a favourable approach for transcatheter aortic valve implantation? A systematic review and meta-analysis comparing local and general anaesthesia. BMJ Open. (2017) 7(9):e016321. 10.1136/bmjopen-2017-016321 28951409 PMC5623571

[101] Liang Y Wang W Wang X Liu M Hei F Guan Y. General anesthesia increased the risk of atrial fibrillation and acute kidney injury in transcatheter aortic valve replacement. Heart Surg Forum. (2021) 24(1):E082–100. 10.1532/hsf.3361 33635259

[102] Sudarsky D Drutin Y Kusniec F Grosman-Rimon L Lubovich A Kinany W Acute kidney injury following transcatheter aortic valve implantation: association with contrast Media dosage and contrast Media based risk predication models. J Clin Med. (2022) 11(5). 10.3390/jcm11051181 35268271 PMC8911230

[103] Zaleska-Kociecka M Dabrowski M Stepinska J. Acute kidney injury after transcatheter aortic valve replacement in the elderly: outcomes and risk management. Clin Interv Aging. (2019) 14:195–201. 10.2147/cia.S149916 30718946 PMC6345183

[104] Giannini F Latib A Jabbour RJ Slavich M Benincasa S Chieffo A The ratio of contrast volume to glomerular filtration rate predicts acute kidney injury and mortality after transcatheter aortic valve implantation. Cardiovasc Revasc Med. (2017) 18(5):349–55. 10.1016/j.carrev.2017.02.011 28342840

[105] Reccia MR Notaristefano F Annunziata R Santucci A Bernardini G Angeli F Incidence of acute kidney injury in patients undergoing transcatheter aortic valve implantation: a novel parameter to choose the better valve? G Ital Cardiol. (2019) 20(10):e26.

[106] Faurie B Souteyrand G Staat P Godin M Caussin C Van Belle E Left ventricular rapid pacing via the valve delivery guidewire in transcatheter aortic valve replacement. JACC Cardiovasc Interv. (2019) 12(24):2449–59. 10.1016/j.jcin.2019.09.029 31857014

[107] Fefer P Bogdan A Grossman Y Berkovitch A Brodov Y Kuperstein R Impact of rapid ventricular pacing on outcome after transcatheter aortic valve replacement. J Am Heart Assoc. (2018) 7(14). 10.1161/jaha.118.009038 29987119 PMC6064853

[108] Blusztein D Raney A Walsh J Nazif T Woods C Daniels D. Best practices in left ventricular pacing for transcatheter aortic valve replacement. Struct Heart. (2023) 7(6):100213. 10.1016/j.shj.2023.100213 38046859 PMC10692352

[109] Neragi-Miandoab S Michler RE. A review of most relevant complications of transcatheter aortic valve implantation. ISRN Cardiol. (2013) 2013:956252. 10.1155/2013/956252 23844292 PMC3703377

[110] Tokuda T Yamamoto M. Vascular management during transcatheter aortic valve replacement. Cardiovasc Interv Ther. (2023) 38(1):18–27. 10.1007/s12928-022-00900-z 36447120

[111] Sardar MR Goldsweig AM Abbott JD Sharaf BL Gordon PC Ehsan A Vascular complications associated with transcatheter aortic valve replacement. Vasc Med. (2017) 22(3):234–44. 10.1177/1358863.17697832 28494713

[112] Ullery BW Jin R Kirker EB Hayes G Siwek L Brevig J Trends in vascular complications and associated treatment strategies following transfemoral transcatheter aortic valve replacement. J Vasc Surg. (2020) 72(4):1313–24.e5. 10.1016/j.jvs.2020.01.050 32169358

[113] Cho JH Han JK Yang HM Koo BK Kim HS. Acute ST-elevation myocardial infarction due to prosthetic valve endocarditis after transcatheter aortic valve implantation. Korean J Intern Med. (2020) 35(4):1020–1. 10.3904/kjim.2018.356 30836743 PMC7373981

[114] Li YJ Liao YB Wei X Feng Y Chen M. Acute myocardial infarction as the initial manifestation of delayed bioprosthesis thrombosis after transcatheter aortic valve replacement. Heart Lung Circ. (2018) 27(4):e46–50. 10.1016/j.hlc.2017.10.026 29198572

[115] Akuka A Landes U Manevich L Rubinshtein R Danenberg HD. Coronary embolism after transcatheter aortic valve replacement-case series and review of literature. Am J Cardiol. (2023) 205:234–40. 10.1016/j.amjcard.2023.07.137 37611416

[116] Faroux L Lhermusier T Vincent F Nombela-Franco L Tchétché D Barbanti M St-Segment elevation myocardial infarction following transcatheter aortic valve replacement. J Am Coll Cardiol. (2021) 77(17):2187–99. 10.1016/j.jacc.2021.03.014 33926655

[117] Gupta T Zimmer J Lahoud RN Murphy HR Harris AH Kolte D National trends and outcomes of acute myocardial infarction after transcatheter aortic valve replacement. JACC Cardiovasc Interv. (2024) 17(10):1267–76. 10.1016/j.jcin.2024.02.026 38530682

[118] Schmidt T Akdag O Wohlmuth P Thielsen T Schewel D Schewel J Histological findings and predictors of cerebral debris from transcatheter aortic valve replacement: the alster experience. J Am Heart Assoc. (2016) 5(11). 10.1161/jaha.116.004399 PMC5210356 27930358

[119] Lansky AJ Ghare MI Pietras C. Carotid disease and stroke after transcatheter aortic valve replacement. Circ Cardiovasc Interv. (2018) 11(6):e006826. 10.1161/circinterventions.118.006826 29895605

[120] Watanabe M Takahashi S Yamaoka H Sueda T Piperata A Zirphile X Comparison of transcarotid vs. Transfemoral transcatheter aortic valve implantation. Circ J. (2018) 82(10):2518–22. 10.1253/circj.CJ-18-0530 30068794

[121] Yahagi K Sato Y Virmani R. A controlled trial of rivaroxaban after transcatheter aortic-valve replacement. N Engl J Med. (2020) 383(2):e8. 10.1056/NEJMc2017351 32640142

[122] Castelo A Teixeira B Grazina A Mendonça T Rodrigues I Garcia Brás P Urgent versus non-urgent transcatheter aortic valve implantation outcomes. Cardiology. (2023) 148(5):469–77. 10.1159/000531815 37429257

[123] Siddiqui SA Kazemian S Gupta T Patel NK Sakhuja R Inglessis I Outcomes of transcatheter aortic valve replacement using third-generation balloon-expandable versus self-expanding valves: a meta-analysis. J Soc Cardiovasc Angiogr Interv. (2024) 3(7):102146. 10.1016/j.jscai.2024.102146 39131997 PMC11308705

[124] Apostolos A Ktenopoulos N Drakopoulou M Ielasi A Panoulas V Baumbach A Effectiveness and safety of myval versus other transcatheter valves in patients undergoing tavi: a meta-analysis. Catheter Cardiovasc Interv. (2025) 106(2):820–9. 10.1002/ccd.31611 40421696 PMC12336787

[125] Koifman E Segev A Fefer P Barbash I Sabbag A Medvedovsky D Comparison of acute kidney injury classifications in patients undergoing transcatheter aortic valve implantation: predictors and long-term outcomes. Catheter Cardiovasc Interv. (2016) 87(3):523–31. 10.1002/ccd.26138 26268940

