# Supplementary material for: Risk factors for acute kidney injury following transcatheter aortic valve replacement: a systematic review and meta-analysis
Source: Front Cardiovasc Med. 2026 Mar 2;13:1684953. doi: 10.3389/fcvm.2026.1684953 (PMC12989351; doi:10.3389/fcvm.2026.1684953)
Supplement: Supplementary file 1 [file Datasheet1.docx]

**Supplementary materials**

TableS1 Literature search strategy

1. Cochrane

ID Search Hits

#1 MeSH descriptor: [Transcatheter Aortic Valve Replacement] explode all trees 516

#2 (Transcatheter Aortic Valve Replacement):ti,ab,kw OR (Transcatheter Aortic Valve Implantation):ti,ab,kw OR (TAVR):ti,ab,kw OR (TAVI):ti,ab,kw 1568

#3 #1 or #2 1568

#4 MeSH descriptor: [Acute Kidney Injury] explode all trees 2472

#5 (Acute kidney injury):ti,ab,kw OR (Acute Kidney Injuries):ti,ab,kw OR (Kidney Injuries, Acute):ti,ab,kw OR (Kidney Injury, Acute):ti,ab,kw OR (Acute Renal Injury):ti,ab,kw 6673

#6 (Acute Renal Injuries):ti,ab,kw OR (Renal Injuries, Acute):ti,ab,kw OR (Renal Injury, Acute):ti,ab,kw OR (Kidney Failure, Acute):ti,ab,kw OR (Acute Kidney Failures):ti,ab,kw 9601

#7 (Kidney Failures, Acute):ti,ab,kw OR (Acute Kidney Failure):ti,ab,kw AND (Acute Renal Failure):ti,ab,kw AND (Acute Renal Failures):ti,ab,kw AND (Renal Failures, Acute):ti,ab,kw 95

#8 (Renal Failure, Acute):ti,ab,kw OR (Renal Insufficiency, Acute):ti,ab,kw OR (Acute Renal Insufficiencies):ti,ab,kw OR (Renal Insufficiencies, Acute):ti,ab,kw OR (Acute Kidney Insufficiency):ti,ab,kw 7570

#9 (Acute Renal Insufficiency):ti,ab,kw OR (Kidney Insufficiency, Acute):ti,ab,kw OR (Acute Kidney Insufficiencies):ti,ab,kw OR (Kidney Insufficiencies, Acute):ti,ab,kw 1451

#10 #4 or #5 or #6 or #7 or #8 or #9 13003

#11 MeSH descriptor: [Risk Factors] explode all trees 38765

#12 (Risk Factors):ti,ab,kw OR (Factor, Risk):ti,ab,kw OR (Risk Factor):ti,ab,kw OR (Population at Risk):ti,ab,kw OR (Populations at Risk):ti,ab,kw 162468

#13 (Risk Scores):ti,ab,kw OR (Risk Score):ti,ab,kw OR (Score, Risk):ti,ab,kw OR (Risk Factor Scores):ti,ab,kw OR (Risk Factor Score):ti,ab,kw 57055

#14 (Score, Risk Factor):ti,ab,kw OR (Health Correlates):ti,ab,kw OR (Correlates, Health):ti,ab,kw OR (Social Risk Factors):ti,ab,kw OR (Factor, Social Risk):ti,ab,kw 16751

#15 (Factors, Social Risk):ti,ab,kw OR (Risk Factor, Social):ti,ab,kw OR (Risk Factors, Social):ti,ab,kw OR (Social Risk Factor):ti,ab,kw 7536

#16 #11 or #12 or #13 or #14 or #15 191712

#17 #3 and #10 and #16 75

2. Embase

| No. | Query | Results | Date |
| --- | --- | --- | --- |
| #54 | #6 AND #32 AND #53 | 536 | 23-Jan-25 |
| #53 | #33 OR #34 OR #35 OR #36 OR #37 OR #38 OR #39 OR #40 OR #41 OR #42 OR #43 OR #44 OR #45 OR #46 OR #47 OR #48 OR #49 OR #50 OR #51 OR #52 | 1985128 | 23-Jan-25 |
| #52 | 'social risk factor':ab,ti | 134 | 23-Jan-25 |
| #51 | 'risk factors, social':ab,ti | 161 | 23-Jan-25 |
| #50 | 'risk factor, social':ab,ti | 9 | 23-Jan-25 |
| #49 | 'factors, social risk':ab,ti | 10 | 23-Jan-25 |
| #48 | 'factor, social risk':ab,ti | 0 | 23-Jan-25 |
| #47 | 'social risk factors':ab,ti | 1535 | 23-Jan-25 |
| #46 | 'correlates, health':ab,ti | 12 | 23-Jan-25 |
| #45 | 'health correlates':ab,ti | 672 | 23-Jan-25 |
| #44 | 'score, risk factor':ab,ti | 17 | 23-Jan-25 |
| #43 | 'risk factor score':ab,ti | 305 | 23-Jan-25 |
| #42 | 'risk factor scores':ab,ti | 154 | 23-Jan-25 |
| #41 | 'score, risk':ab,ti | 1418 | 23-Jan-25 |
| #40 | 'risk score':ab,ti | 54089 | 23-Jan-25 |
| #39 | 'risk scores':ab,ti | 25829 | 23-Jan-25 |
| #38 | 'populations at risk':ab,ti | 3890 | 23-Jan-25 |
| #37 | 'population at risk':ab,ti | 6225 | 23-Jan-25 |
| #36 | 'risk factor':ab,ti | 431653 | 23-Jan-25 |
| #35 | 'factor, risk':ab,ti | 364 | 23-Jan-25 |
| #34 | 'risk factors':ab,ti | 932086 | 23-Jan-25 |
| #33 | 'risk factor'/exp | 1503840 | 23-Jan-25 |
| #32 | #7 OR #8 OR #9 OR #10 OR #11 OR #12 OR #13 OR #14 OR #15 OR #16 OR #17 OR #18 OR #19 OR #20 OR #21 OR #22 OR #23 OR #24 OR #25 OR #26 OR #27 OR #28 OR #29 OR #30 OR #31 | 166050 | 23-Jan-25 |
| #31 | 'kidney insufficiencies, acute':ab,ti | 0 | 23-Jan-25 |
| #30 | 'acute kidney insufficiencies':ab,ti | 1 | 23-Jan-25 |
| #29 | 'kidney insufficiency, acute':ab,ti | 1 | 23-Jan-25 |
| #28 | 'acute renal insufficiency':ab,ti | 2163 | 23-Jan-25 |
| #27 | 'acute kidney insufficiency':ab,ti | 148 | 23-Jan-25 |
| #26 | 'renal insufficiencies, acute':ab,ti | 0 | 23-Jan-25 |
| #25 | 'acute renal insufficiencies':ab,ti | 15 | 23-Jan-25 |
| #24 | 'renal insufficiency, acute':ab,ti | 50 | 23-Jan-25 |
| #23 | 'renal failure, acute':ab,ti | 435 | 23-Jan-25 |
| #22 | 'renal failures, acute':ab,ti | 1 | 23-Jan-25 |
| #21 | 'acute renal failures':ab,ti | 75 | 23-Jan-25 |
| #20 | 'acute renal failure':ab,ti | 36069 | 23-Jan-25 |
| #19 | 'acute kidney failure':ab,ti | 2142 | 23-Jan-25 |
| #18 | 'kidney failures, acute':ab,ti | 0 | 23-Jan-25 |
| #17 | 'acute kidney failures':ab,ti | 7 | 23-Jan-25 |
| #16 | 'kidney failure, acute':ab,ti | 39 | 23-Jan-25 |
| #15 | 'renal injury, acute':ab,ti | 26 | 23-Jan-25 |
| #14 | 'renal injuries, acute':ab,ti | 1 | 23-Jan-25 |
| #13 | 'acute renal injuries':ab,ti | 19 | 23-Jan-25 |
| #12 | 'acute renal injury':ab,ti | 2342 | 23-Jan-25 |
| #11 | 'kidney injury, acute':ab,ti | 295 | 23-Jan-25 |
| #10 | 'kidney injuries, acute':ab,ti | 3 | 23-Jan-25 |
| #9 | 'acute kidney injuries':ab,ti | 316 | 23-Jan-25 |
| #8 | 'acute kidney injury':ab,ti | 73386 | 23-Jan-25 |
| #7 | 'acute kidney failure'/exp | 143815 | 23-Jan-25 |
| #6 | #1 OR #2 OR #3 OR #4 OR #5 | 41055 | 23-Jan-25 |
| #5 | 'tavi':ab,ti | 14344 | 23-Jan-25 |
| #4 | 'tavr':ab,ti | 12973 | 23-Jan-25 |
| #3 | 'transcatheter aortic valve implantation':ab,ti | 14051 | 23-Jan-25 |
| #2 | 'transcatheter aortic valve replacement':ab,ti | 13780 | 23-Jan-25 |
| #1 | 'transcatheter aortic valve implantation'/exp | 37468 | 23-Jan-25 |

3. PubMed

| Search number | Query | Sort By | Filters | Search Details | Results | Time |
| --- | --- | --- | --- | --- | --- | --- |
| 10 | (((Transcatheter Aortic Valve Replacement[MeSH Terms]) OR ((((Transcatheter Aortic Valve Replacement[Title/Abstract]) OR (Transcatheter Aortic Valve Implantation[Title/Abstract])) OR (TAVR[Title/Abstract])) OR (TAVI[Title/Abstract]))) AND ((Acute kidney injury[MeSH Terms]) OR ((((((((((((((((((((((((Acute kidney injury[Title/Abstract]) OR (Acute Kidney Injuries[Title/Abstract])) OR (Kidney Injuries, Acute[Title/Abstract])) OR (Kidney Injury, Acute[Title/Abstract])) OR (Acute Renal Injury[Title/Abstract])) OR (Acute Renal Injuries[Title/Abstract])) OR (Renal Injuries, Acute[Title/Abstract])) OR (Renal Injury, Acute[Title/Abstract])) OR (Kidney Failure, Acute[Title/Abstract])) OR (Acute Kidney Failures[Title/Abstract])) OR (Kidney Failures, Acute[Title/Abstract])) OR (Acute Kidney Failure[Title/Abstract])) OR (Acute Renal Failure[Title/Abstract])) OR (Acute Renal Failures[Title/Abstract])) OR (Renal Failures, Acute[Title/Abstract])) OR (Renal Failure, Acute[Title/Abstract])) OR (Renal Insufficiency, Acute[Title/Abstract])) OR (Acute Renal Insufficiencies[Title/Abstract])) OR (Renal Insufficiencies, Acute[Title/Abstract])) OR (Acute Kidney Insufficiency[Title/Abstract])) OR (Acute Renal Insufficiency[Title/Abstract])) OR (Kidney Insufficiency, Acute[Title/Abstract])) OR (Acute Kidney Insufficiencies[Title/Abstract])) OR (Kidney Insufficiencies, Acute[Title/Abstract])))) AND ((Risk Factors[MeSH Terms]) OR (((((((((((((((((((Risk Factors[Title/Abstract]) OR (Factor, Risk[Title/Abstract])) OR (Risk Factor[Title/Abstract])) OR (Population at Risk[Title/Abstract])) OR (Populations at Risk[Title/Abstract])) OR (Risk Scores[Title/Abstract])) OR (Risk Score[Title/Abstract])) OR (Score, Risk[Title/Abstract])) OR (Risk Factor Scores[Title/Abstract])) OR (Risk Factor Score[Title/Abstract])) OR (Score, Risk Factor[Title/Abstract])) OR (Health Correlates[Title/Abstract])) OR (Correlates, Health[Title/Abstract])) OR (Social Risk Factors[Title/Abstract])) OR (Factor, Social Risk[Title/Abstract])) OR (Factors, Social Risk[Title/Abstract])) OR (Risk Factor, Social[Title/Abstract])) OR (Risk Factors, Social[Title/Abstract])) OR (Social Risk Factor[Title/Abstract]))) | Most Recent |  | ("transcatheter aortic valve replacement"[MeSH Terms] OR ("transcatheter aortic valve replacement"[Title/Abstract] OR "transcatheter aortic valve implantation"[Title/Abstract] OR "TAVR"[Title/Abstract] OR "TAVI"[Title/Abstract])) AND ("acute kidney injury"[MeSH Terms] OR ("acute kidney injury"[Title/Abstract] OR "acute kidney injuries"[Title/Abstract] OR "kidney injuries acute"[Title/Abstract] OR "kidney injury acute"[Title/Abstract] OR "acute renal injury"[Title/Abstract] OR "acute renal injuries"[Title/Abstract] OR (("Renal"[All Fields] OR "renals"[All Fields]) AND "injuries acute"[Title/Abstract]) OR "renal injury acute"[Title/Abstract] OR "kidney failure acute"[Title/Abstract] OR "acute kidney failures"[Title/Abstract] OR (("Kidney"[MeSH Terms] OR "Kidney"[All Fields] OR "kidneys"[All Fields] OR "kidney s"[All Fields]) AND "failures acute"[Title/Abstract]) OR "acute kidney failure"[Title/Abstract] OR "acute renal failure"[Title/Abstract] OR "acute renal failures"[Title/Abstract] OR (("Renal"[All Fields] OR "renals"[All Fields]) AND "failures acute"[Title/Abstract]) OR "renal failure acute"[Title/Abstract] OR "renal insufficiency acute"[Title/Abstract] OR "acute renal insufficiencies"[Title/Abstract] OR (("renal insufficiency"[MeSH Terms] OR ("Renal"[All Fields] AND "Insufficiency"[All Fields]) OR "renal insufficiency"[All Fields] OR ("Renal"[All Fields] AND "Insufficiencies"[All Fields]) OR "renal insufficiencies"[All Fields]) AND "Acute"[Title/Abstract]) OR "acute kidney insufficiency"[Title/Abstract] OR "acute renal insufficiency"[Title/Abstract] OR (("Kidney"[MeSH Terms] OR "Kidney"[All Fields] OR "kidneys"[All Fields] OR "kidney s"[All Fields]) AND "insufficiency acute"[Title/Abstract]) OR "acute kidney insufficiencies"[Title/Abstract] OR (("renal insufficiency"[MeSH Terms] OR ("Renal"[All Fields] AND "Insufficiency"[All Fields]) OR "renal insufficiency"[All Fields] OR ("Kidney"[All Fields] AND "Insufficiencies"[All Fields]) OR "kidney insufficiencies"[All Fields]) AND "Acute"[Title/Abstract]))) AND ("risk factors"[MeSH Terms] OR ("risk factors"[Title/Abstract] OR "factor risk"[Title/Abstract] OR "risk factor"[Title/Abstract] OR "population at risk"[Title/Abstract] OR "populations at risk"[Title/Abstract] OR "risk scores"[Title/Abstract] OR "risk score"[Title/Abstract] OR "score risk"[Title/Abstract] OR "risk factor scores"[Title/Abstract] OR "risk factor score"[Title/Abstract] OR "score risk factor"[Title/Abstract] OR "health correlates"[Title/Abstract] OR "correlates health"[Title/Abstract] OR "social risk factors"[Title/Abstract] OR (("Factor"[All Fields] OR "factor s"[All Fields] OR "Factors"[All Fields]) AND "social risk"[Title/Abstract]) OR "factors social risk"[Title/Abstract] OR "risk factor social"[Title/Abstract] OR "risk factors social"[Title/Abstract] OR "social risk factor"[Title/Abstract])) | 527 | 5:04:54 |
| 9 | (Risk Factors[MeSH Terms]) OR (((((((((((((((((((Risk Factors[Title/Abstract]) OR (Factor, Risk[Title/Abstract])) OR (Risk Factor[Title/Abstract])) OR (Population at Risk[Title/Abstract])) OR (Populations at Risk[Title/Abstract])) OR (Risk Scores[Title/Abstract])) OR (Risk Score[Title/Abstract])) OR (Score, Risk[Title/Abstract])) OR (Risk Factor Scores[Title/Abstract])) OR (Risk Factor Score[Title/Abstract])) OR (Score, Risk Factor[Title/Abstract])) OR (Health Correlates[Title/Abstract])) OR (Correlates, Health[Title/Abstract])) OR (Social Risk Factors[Title/Abstract])) OR (Factor, Social Risk[Title/Abstract])) OR (Factors, Social Risk[Title/Abstract])) OR (Risk Factor, Social[Title/Abstract])) OR (Risk Factors, Social[Title/Abstract])) OR (Social Risk Factor[Title/Abstract])) | Most Recent |  | "risk factors"[MeSH Terms] OR ("risk factors"[Title/Abstract] OR "factor risk"[Title/Abstract] OR "risk factor"[Title/Abstract] OR "population at risk"[Title/Abstract] OR "populations at risk"[Title/Abstract] OR "risk scores"[Title/Abstract] OR "risk score"[Title/Abstract] OR "score risk"[Title/Abstract] OR "risk factor scores"[Title/Abstract] OR "risk factor score"[Title/Abstract] OR "score risk factor"[Title/Abstract] OR "health correlates"[Title/Abstract] OR "correlates health"[Title/Abstract] OR "social risk factors"[Title/Abstract] OR (("Factor"[All Fields] OR "factor s"[All Fields] OR "Factors"[All Fields]) AND "social risk"[Title/Abstract]) OR "factors social risk"[Title/Abstract] OR "risk factor social"[Title/Abstract] OR "risk factors social"[Title/Abstract] OR "social risk factor"[Title/Abstract]) | 1,523,473 | 5:03:52 |
| 8 | ((((((((((((((((((Risk Factors[Title/Abstract]) OR (Factor, Risk[Title/Abstract])) OR (Risk Factor[Title/Abstract])) OR (Population at Risk[Title/Abstract])) OR (Populations at Risk[Title/Abstract])) OR (Risk Scores[Title/Abstract])) OR (Risk Score[Title/Abstract])) OR (Score, Risk[Title/Abstract])) OR (Risk Factor Scores[Title/Abstract])) OR (Risk Factor Score[Title/Abstract])) OR (Score, Risk Factor[Title/Abstract])) OR (Health Correlates[Title/Abstract])) OR (Correlates, Health[Title/Abstract])) OR (Social Risk Factors[Title/Abstract])) OR (Factor, Social Risk[Title/Abstract])) OR (Factors, Social Risk[Title/Abstract])) OR (Risk Factor, Social[Title/Abstract])) OR (Risk Factors, Social[Title/Abstract])) OR (Social Risk Factor[Title/Abstract]) | Most Recent |  | "risk factors"[Title/Abstract] OR "factor risk"[Title/Abstract] OR "risk factor"[Title/Abstract] OR "population at risk"[Title/Abstract] OR "populations at risk"[Title/Abstract] OR "risk scores"[Title/Abstract] OR "risk score"[Title/Abstract] OR "score risk"[Title/Abstract] OR "risk factor scores"[Title/Abstract] OR "risk factor score"[Title/Abstract] OR "score risk factor"[Title/Abstract] OR "health correlates"[Title/Abstract] OR "correlates health"[Title/Abstract] OR "social risk factors"[Title/Abstract] OR (("Factor"[All Fields] OR "factor s"[All Fields] OR "Factors"[All Fields]) AND "social risk"[Title/Abstract]) OR "factors social risk"[Title/Abstract] OR "risk factor social"[Title/Abstract] OR "risk factors social"[Title/Abstract] OR "social risk factor"[Title/Abstract] | 901,965 | 5:03:43 |
| 7 | Risk Factors[MeSH Terms] | Most Recent |  | "risk factors"[MeSH Terms] | 1,019,611 | 5:01:13 |
| 6 | (Acute kidney injury[MeSH Terms]) OR ((((((((((((((((((((((((Acute kidney injury[Title/Abstract]) OR (Acute Kidney Injuries[Title/Abstract])) OR (Kidney Injuries, Acute[Title/Abstract])) OR (Kidney Injury, Acute[Title/Abstract])) OR (Acute Renal Injury[Title/Abstract])) OR (Acute Renal Injuries[Title/Abstract])) OR (Renal Injuries, Acute[Title/Abstract])) OR (Renal Injury, Acute[Title/Abstract])) OR (Kidney Failure, Acute[Title/Abstract])) OR (Acute Kidney Failures[Title/Abstract])) OR (Kidney Failures, Acute[Title/Abstract])) OR (Acute Kidney Failure[Title/Abstract])) OR (Acute Renal Failure[Title/Abstract])) OR (Acute Renal Failures[Title/Abstract])) OR (Renal Failures, Acute[Title/Abstract])) OR (Renal Failure, Acute[Title/Abstract])) OR (Renal Insufficiency, Acute[Title/Abstract])) OR (Acute Renal Insufficiencies[Title/Abstract])) OR (Renal Insufficiencies, Acute[Title/Abstract])) OR (Acute Kidney Insufficiency[Title/Abstract])) OR (Acute Renal Insufficiency[Title/Abstract])) OR (Kidney Insufficiency, Acute[Title/Abstract])) OR (Acute Kidney Insufficiencies[Title/Abstract])) OR (Kidney Insufficiencies, Acute[Title/Abstract])) | Most Recent |  | "acute kidney injury"[MeSH Terms] OR ("acute kidney injury"[Title/Abstract] OR "acute kidney injuries"[Title/Abstract] OR "kidney injuries acute"[Title/Abstract] OR "kidney injury acute"[Title/Abstract] OR "acute renal injury"[Title/Abstract] OR "acute renal injuries"[Title/Abstract] OR (("Renal"[All Fields] OR "renals"[All Fields]) AND "injuries acute"[Title/Abstract]) OR "renal injury acute"[Title/Abstract] OR "kidney failure acute"[Title/Abstract] OR "acute kidney failures"[Title/Abstract] OR (("Kidney"[MeSH Terms] OR "Kidney"[All Fields] OR "kidneys"[All Fields] OR "kidney s"[All Fields]) AND "failures acute"[Title/Abstract]) OR "acute kidney failure"[Title/Abstract] OR "acute renal failure"[Title/Abstract] OR "acute renal failures"[Title/Abstract] OR (("Renal"[All Fields] OR "renals"[All Fields]) AND "failures acute"[Title/Abstract]) OR "renal failure acute"[Title/Abstract] OR "renal insufficiency acute"[Title/Abstract] OR "acute renal insufficiencies"[Title/Abstract] OR (("renal insufficiency"[MeSH Terms] OR ("Renal"[All Fields] AND "Insufficiency"[All Fields]) OR "renal insufficiency"[All Fields] OR ("Renal"[All Fields] AND "Insufficiencies"[All Fields]) OR "renal insufficiencies"[All Fields]) AND "Acute"[Title/Abstract]) OR "acute kidney insufficiency"[Title/Abstract] OR "acute renal insufficiency"[Title/Abstract] OR (("Kidney"[MeSH Terms] OR "Kidney"[All Fields] OR "kidneys"[All Fields] OR "kidney s"[All Fields]) AND "insufficiency acute"[Title/Abstract]) OR "acute kidney insufficiencies"[Title/Abstract] OR (("renal insufficiency"[MeSH Terms] OR ("Renal"[All Fields] AND "Insufficiency"[All Fields]) OR "renal insufficiency"[All Fields] OR ("Kidney"[All Fields] AND "Insufficiencies"[All Fields]) OR "kidney insufficiencies"[All Fields]) AND "Acute"[Title/Abstract])) | 102,983 | 4:45:56 |
| 5 | (((((((((((((((((((((((Acute kidney injury[Title/Abstract]) OR (Acute Kidney Injuries[Title/Abstract])) OR (Kidney Injuries, Acute[Title/Abstract])) OR (Kidney Injury, Acute[Title/Abstract])) OR (Acute Renal Injury[Title/Abstract])) OR (Acute Renal Injuries[Title/Abstract])) OR (Renal Injuries, Acute[Title/Abstract])) OR (Renal Injury, Acute[Title/Abstract])) OR (Kidney Failure, Acute[Title/Abstract])) OR (Acute Kidney Failures[Title/Abstract])) OR (Kidney Failures, Acute[Title/Abstract])) OR (Acute Kidney Failure[Title/Abstract])) OR (Acute Renal Failure[Title/Abstract])) OR (Acute Renal Failures[Title/Abstract])) OR (Renal Failures, Acute[Title/Abstract])) OR (Renal Failure, Acute[Title/Abstract])) OR (Renal Insufficiency, Acute[Title/Abstract])) OR (Acute Renal Insufficiencies[Title/Abstract])) OR (Renal Insufficiencies, Acute[Title/Abstract])) OR (Acute Kidney Insufficiency[Title/Abstract])) OR (Acute Renal Insufficiency[Title/Abstract])) OR (Kidney Insufficiency, Acute[Title/Abstract])) OR (Acute Kidney Insufficiencies[Title/Abstract])) OR (Kidney Insufficiencies, Acute[Title/Abstract]) | Most Recent |  | "acute kidney injury"[Title/Abstract] OR "acute kidney injuries"[Title/Abstract] OR "kidney injuries acute"[Title/Abstract] OR "kidney injury acute"[Title/Abstract] OR "acute renal injury"[Title/Abstract] OR "acute renal injuries"[Title/Abstract] OR (("Renal"[All Fields] OR "renals"[All Fields]) AND "injuries acute"[Title/Abstract]) OR "renal injury acute"[Title/Abstract] OR "kidney failure acute"[Title/Abstract] OR "acute kidney failures"[Title/Abstract] OR (("Kidney"[MeSH Terms] OR "Kidney"[All Fields] OR "kidneys"[All Fields] OR "kidney s"[All Fields]) AND "failures acute"[Title/Abstract]) OR "acute kidney failure"[Title/Abstract] OR "acute renal failure"[Title/Abstract] OR "acute renal failures"[Title/Abstract] OR (("Renal"[All Fields] OR "renals"[All Fields]) AND "failures acute"[Title/Abstract]) OR "renal failure acute"[Title/Abstract] OR "renal insufficiency acute"[Title/Abstract] OR "acute renal insufficiencies"[Title/Abstract] OR (("renal insufficiency"[MeSH Terms] OR ("Renal"[All Fields] AND "Insufficiency"[All Fields]) OR "renal insufficiency"[All Fields] OR ("Renal"[All Fields] AND "Insufficiencies"[All Fields]) OR "renal insufficiencies"[All Fields]) AND "Acute"[Title/Abstract]) OR "acute kidney insufficiency"[Title/Abstract] OR "acute renal insufficiency"[Title/Abstract] OR (("Kidney"[MeSH Terms] OR "Kidney"[All Fields] OR "kidneys"[All Fields] OR "kidney s"[All Fields]) AND "insufficiency acute"[Title/Abstract]) OR "acute kidney insufficiencies"[Title/Abstract] OR (("renal insufficiency"[MeSH Terms] OR ("Renal"[All Fields] AND "Insufficiency"[All Fields]) OR "renal insufficiency"[All Fields] OR ("Kidney"[All Fields] AND "Insufficiencies"[All Fields]) OR "kidney insufficiencies"[All Fields]) AND "Acute"[Title/Abstract]) | 88,212 | 4:45:41 |
| 4 | Acute kidney injury[MeSH Terms] | Most Recent |  | "acute kidney injury"[MeSH Terms] | 60,054 | 4:42:14 |
| 3 | (Transcatheter Aortic Valve Replacement[MeSH Terms]) OR ((((Transcatheter Aortic Valve Replacement[Title/Abstract]) OR (Transcatheter Aortic Valve Implantation[Title/Abstract])) OR (TAVR[Title/Abstract])) OR (TAVI[Title/Abstract])) | Most Recent |  | "transcatheter aortic valve replacement"[MeSH Terms] OR "transcatheter aortic valve replacement"[Title/Abstract] OR "transcatheter aortic valve implantation"[Title/Abstract] OR "TAVR"[Title/Abstract] OR "TAVI"[Title/Abstract] | 20,562 | 4:41:23 |
| 2 | (((Transcatheter Aortic Valve Replacement[Title/Abstract]) OR (Transcatheter Aortic Valve Implantation[Title/Abstract])) OR (TAVR[Title/Abstract])) OR (TAVI[Title/Abstract]) | Most Recent |  | "transcatheter aortic valve replacement"[Title/Abstract] OR "transcatheter aortic valve implantation"[Title/Abstract] OR "TAVR"[Title/Abstract] OR "TAVI"[Title/Abstract] | 19,132 | 4:39:39 |
| 1 | Transcatheter Aortic Valve Replacement[MeSH Terms] | Most Recent |  | "transcatheter aortic valve replacement"[MeSH Terms] | 12,640 | 4:38:14 |

4. web of science

| 权限 | # | 检索式 | 数据库 | 检索结果 | 运行日期 |
| --- | --- | --- | --- | --- | --- |
| - WOS.SCI: 1900 to 2025 - WOS.AHCI: 1975 to 2025 - WOS.BHCI: 2005 to 2025 - WOS.BSCI: 2005 to 2025 - WOS.ESCI: 2020 to 2025 - WOS.ISTP: 1990 to 2025 - WOS.SSCI: 1900 to 2025 - WOS.ISSHP: 1990 to 2025 | 1 | TS=(Transcatheter Aortic Valve Replacement) OR TS=(Transcatheter Aortic Valve Implantation) OR TS=(TAVR) OR TS=（TAVI） | Web of Science 核心合集 | 26969 | Fri Jan 24 2025 20:02:08 GMT+0800 (中国标准时间) |
| - WOS.SCI: 1900 to 2025 - WOS.AHCI: 1975 to 2025 - WOS.BHCI: 2005 to 2025 - WOS.BSCI: 2005 to 2025 - WOS.ESCI: 2020 to 2025 - WOS.ISTP: 1990 to 2025 - WOS.SSCI: 1900 to 2025 - WOS.ISSHP: 1990 to 2025 | 2 | TS=(Acute kidney injury) OR TS= (Acute Kidney Injuries) OR TS=(Kidney Injuries, Acute) OR TS=(Kidney Injury, Acute) OR TS=(Acute Renal Injury) OR TS=(Acute Renal Injuries) OR TS=(Renal Injuries, Acute) OR TS=(Renal Injury, Acute) OR TS=(Kidney Failure, Acute) OR TS=(Acute Kidney Failures) OR TS=(Kidney Failures, Acute) OR TS=(Acute Kidney Failure) OR TS=(Acute Renal Failure) OR TS=(Acute Renal Failures) OR TS=(Renal Failures, Acute) OR TS=(Renal Failure, Acute) OR TS=(Renal Insufficiency, Acute) OR TS=(Acute Renal Insufficiencies) OR TS=(Renal Insufficiencies, Acute) OR TS=(Acute Kidney Insufficiency) OR TS=(Acute Renal Insufficiency) OR TS=(Kidney Insufficiency, Acute) OR TS=(Acute Kidney Insufficiencies) OR TS=(Kidney Insufficiencies, Acute) | Web of Science 核心合集 | 116819 | Fri Jan 24 2025 20:11:45 GMT+0800 (中国标准时间) |
| - WOS.SCI: 1900 to 2025 - WOS.AHCI: 1975 to 2025 - WOS.BHCI: 2005 to 2025 - WOS.BSCI: 2005 to 2025 - WOS.ESCI: 2020 to 2025 - WOS.ISTP: 1990 to 2025 - WOS.SSCI: 1900 to 2025 - WOS.ISSHP: 1990 to 2025 | 3 | TS=(Risk Factors) OR TS=(Factor, Risk) OR TS=(Risk Factor) OR TS=(Population at Risk) OR TS=(Populations at Risk) OR TS=(Risk Scores) OR TS=(Risk Score) OR TS=(Score, Risk) OR TS=(Risk Factor Scores) OR TS=(Risk Factor Score) OR TS=(Score, Risk Factor) OR TS=(Health Correlates) OR TS=(Correlates, Health) OR TS=(Social Risk Factors) OR TS=(Factor, Social Risk) OR TS=(Factors, Social Risk) OR TS=(Risk Factor, Social) OR TS=(Risk Factors, Social) OR TS=(Social Risk Factor) | Web of Science 核心合集 | 2315685 | Fri Jan 24 2025 20:15:47 GMT+0800 (中国标准时间) |
| - WOS.SCI: 1900 to 2025 - WOS.AHCI: 1975 to 2025 - WOS.BHCI: 2005 to 2025 - WOS.BSCI: 2005 to 2025 - WOS.ESCI: 2020 to 2025 - WOS.ISTP: 1990 to 2025 - WOS.SSCI: 1900 to 2025 - WOS.ISSHP: 1990 to 2025 | 4 | #3 AND #2 AND #1 | Web of Science 核心合集 | 462 | Fri Jan 24 2025 20:15:56 GMT+0800 (中国标准时间) |

TableS2 GRADE evaluation

| Risk factors | Grade |
| --- | --- |
| ***Hypertension*** | Moderate |
| ***Coronary Artery Disease*** | Low |
| ***Peripheral Vascular Disease*** | Low |
| ***Chronic Kidney Disease*** | Low |
| ***Transapical Access*** | Low |
| ***Serum Creatinine Level*** | Low |
| ***STS Score*** | Low |
| ***Prior Stroke*** | Low |


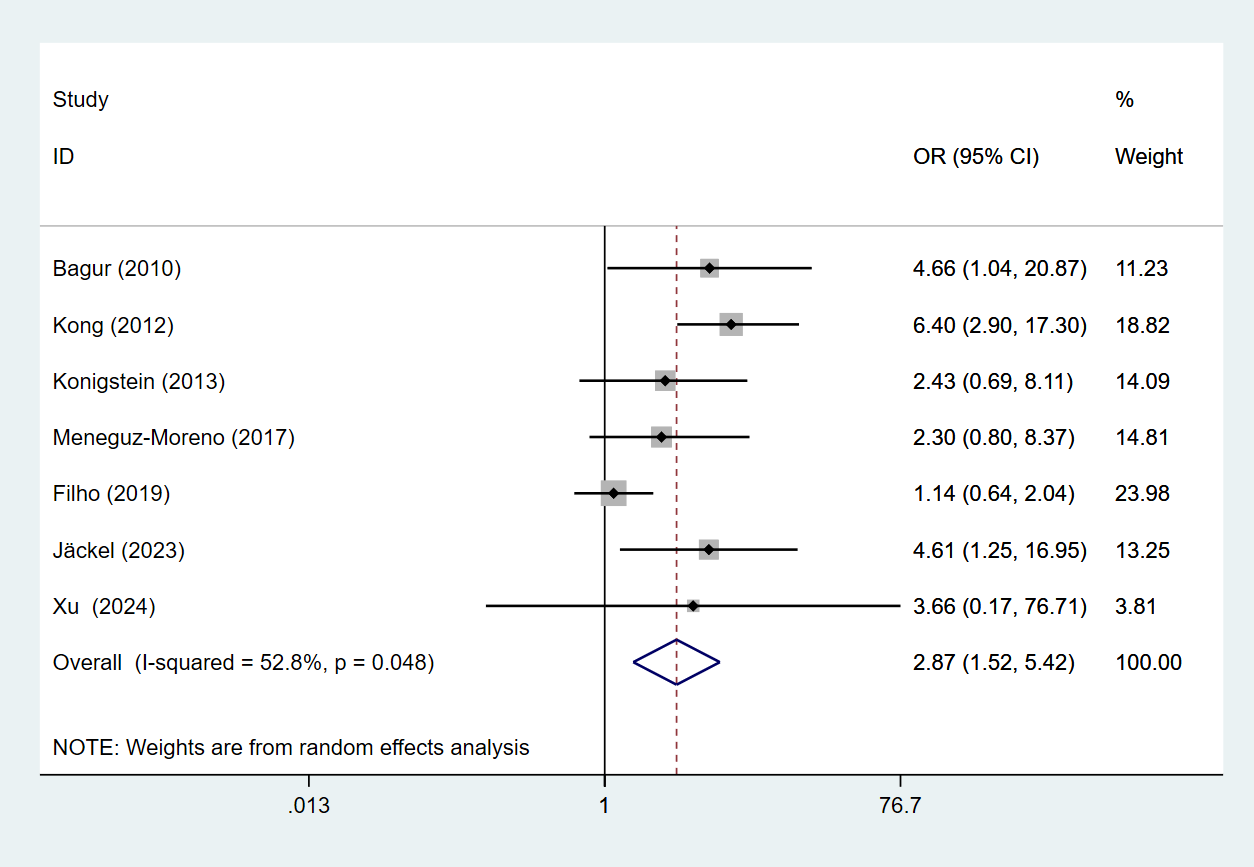


Figure S1 Hypertension Multivariable Forest Plot


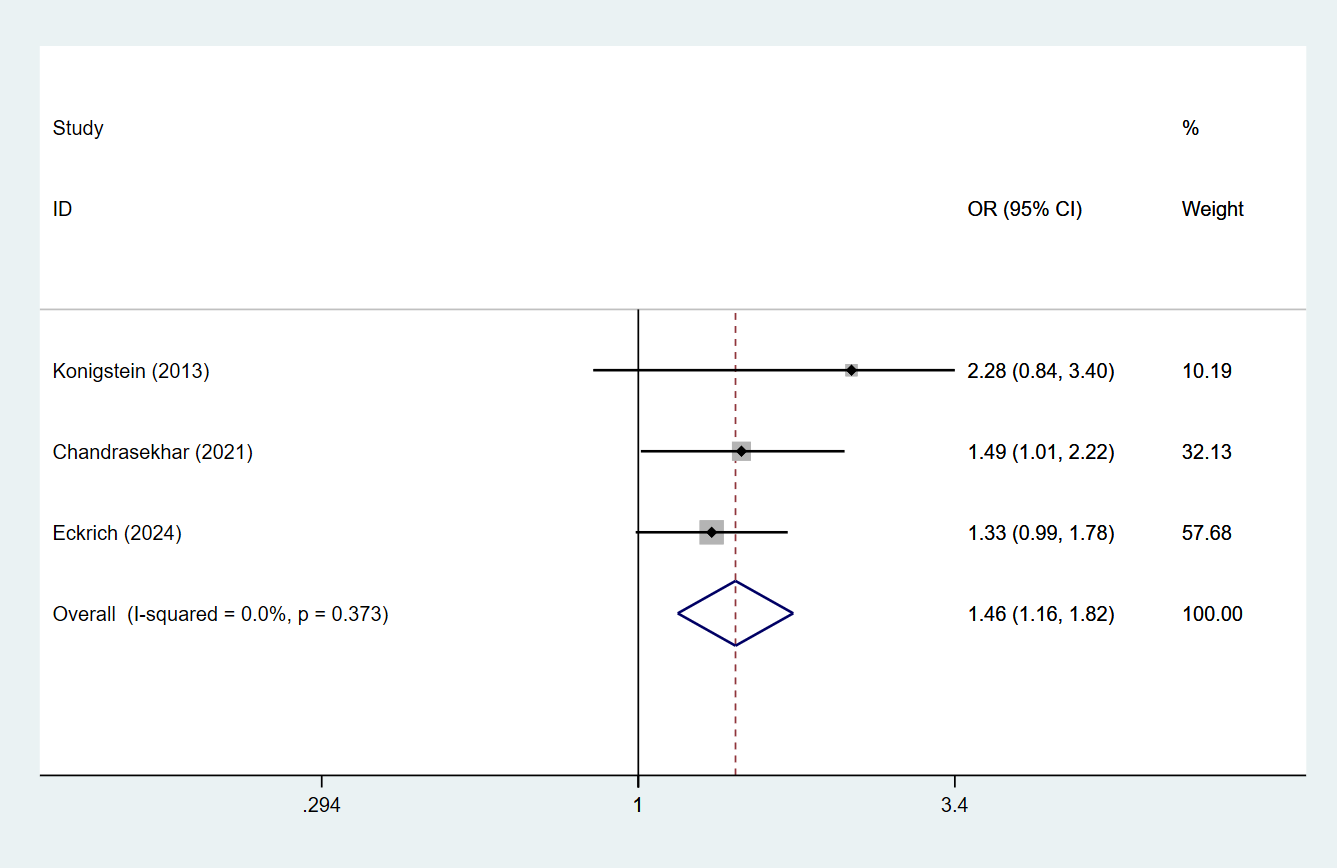


Figure S2 Coronary artery disease Multivariable Forest Plot


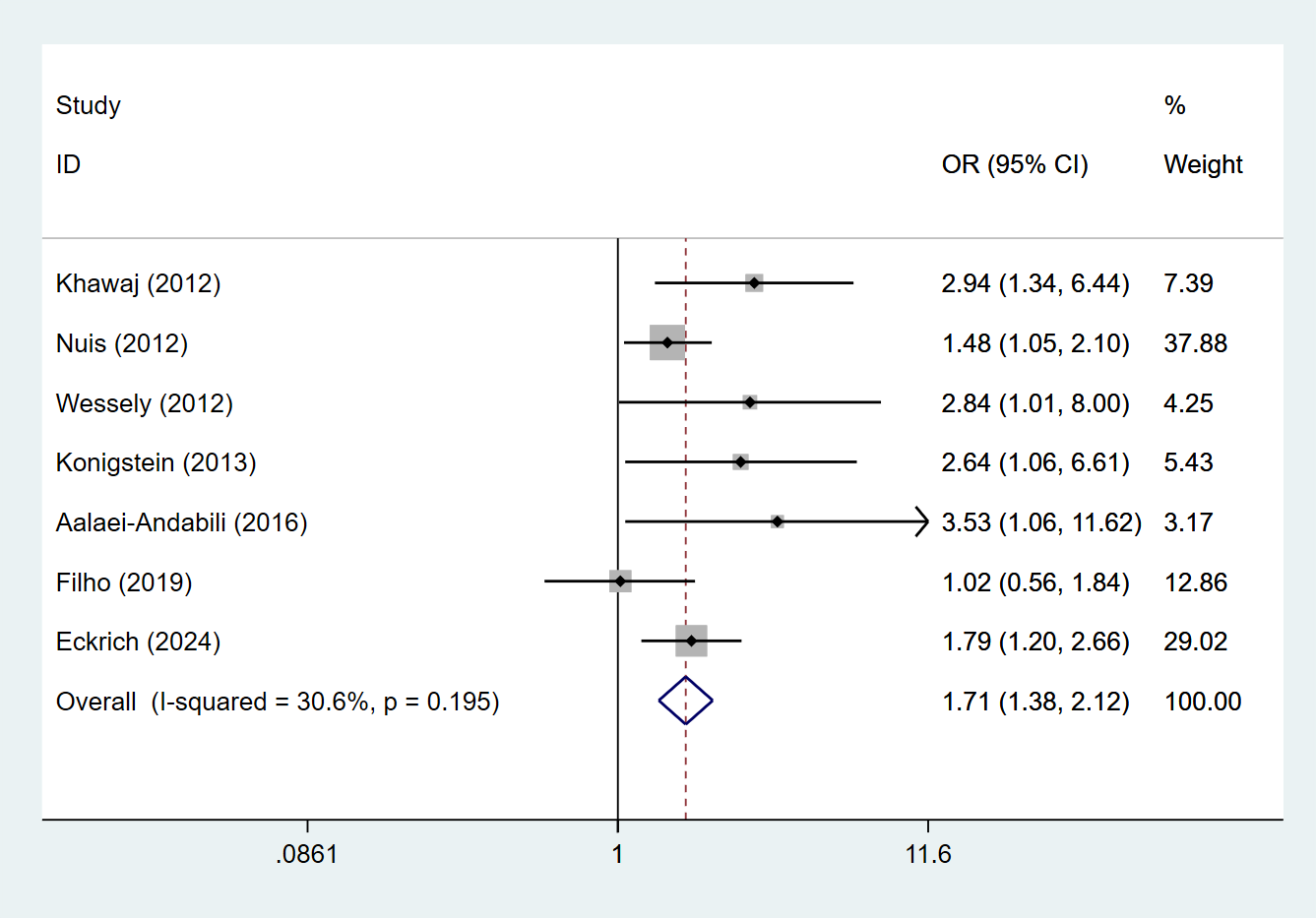


Figure S3 Peripheral vascular disease Multivariable Forest Plot


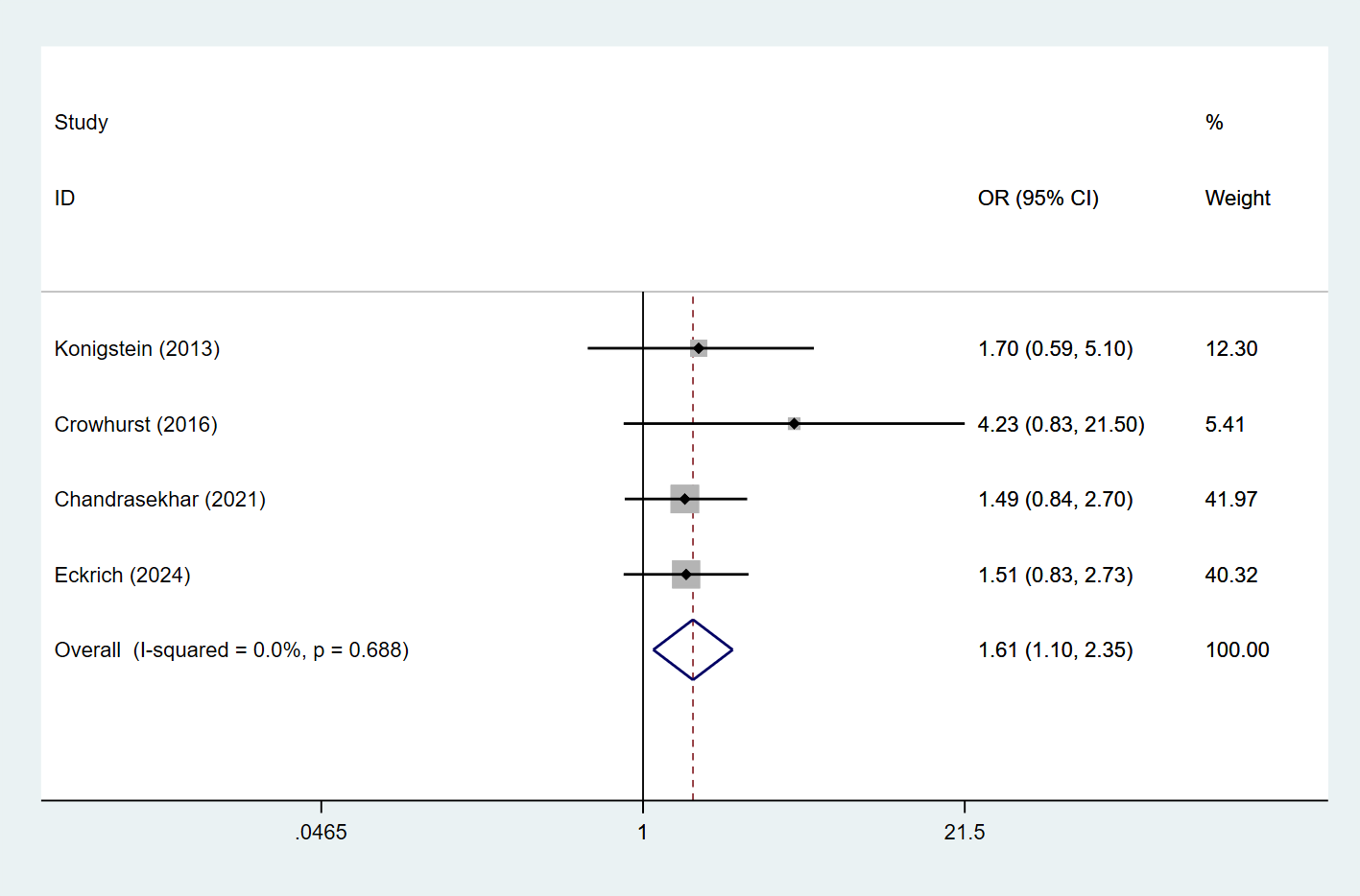


Figure S4 History of stroke Multivariable Forest Plot


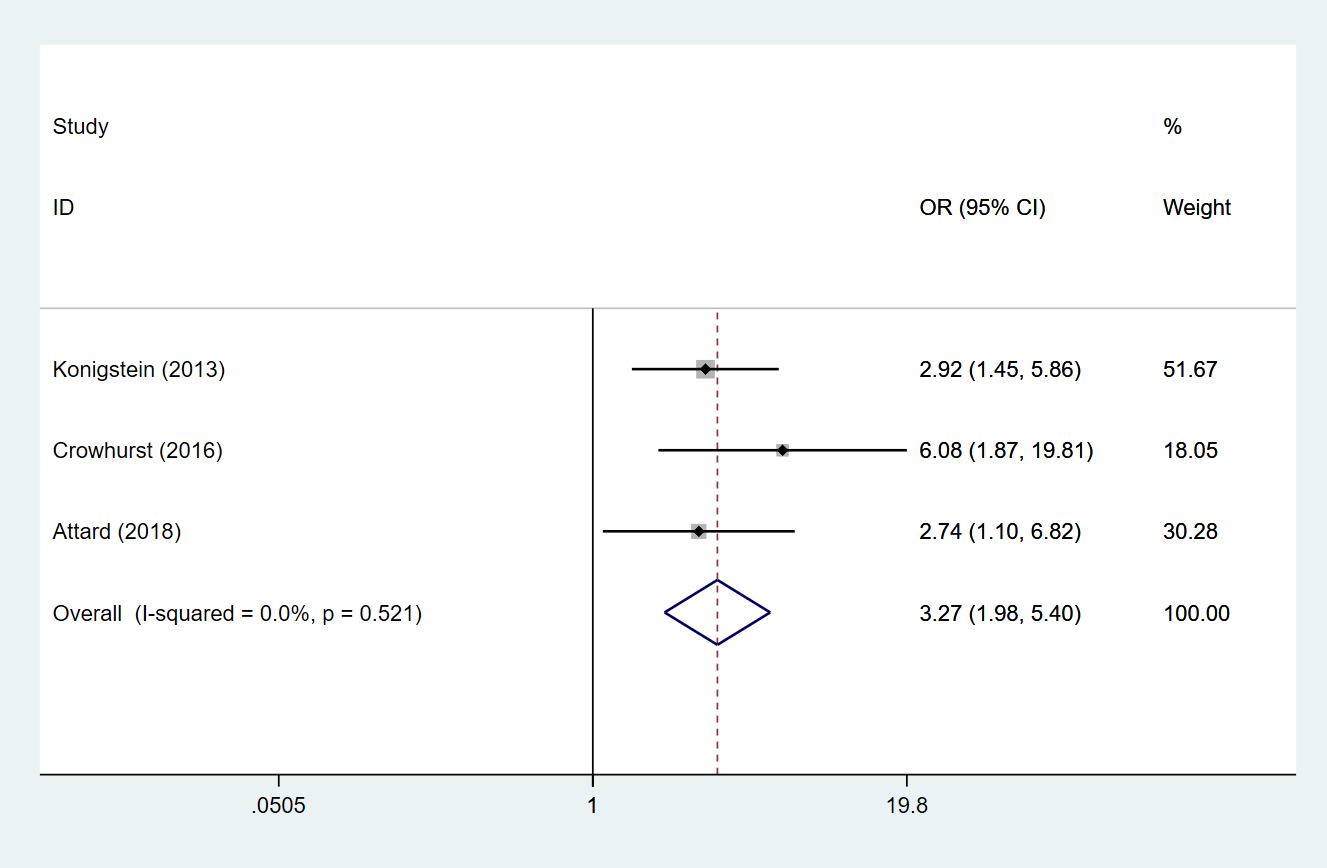


Figure S5 CKD Multivariable Forest Plot


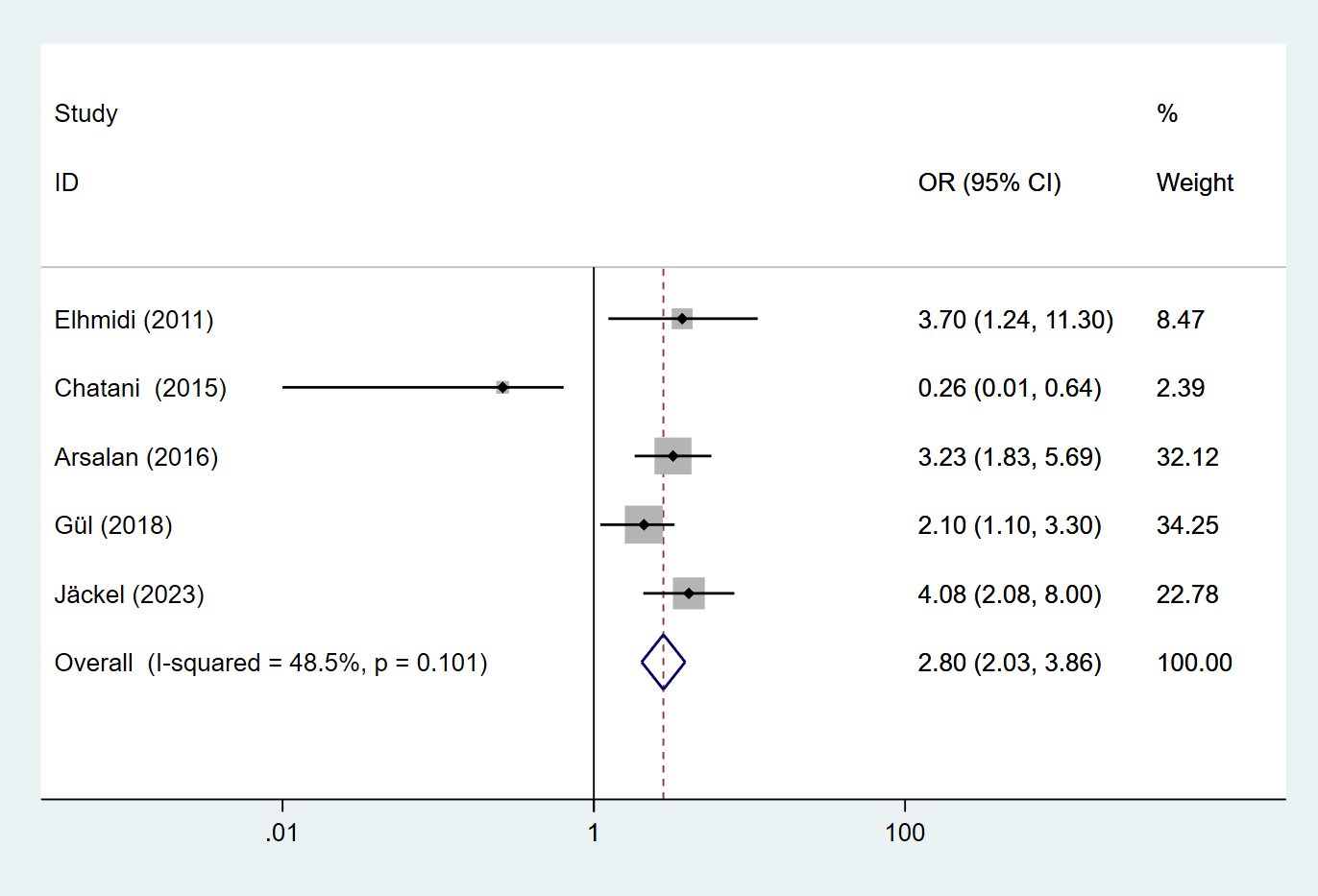


Figure S6 Serum creatinine level Multivariable Forest Plot


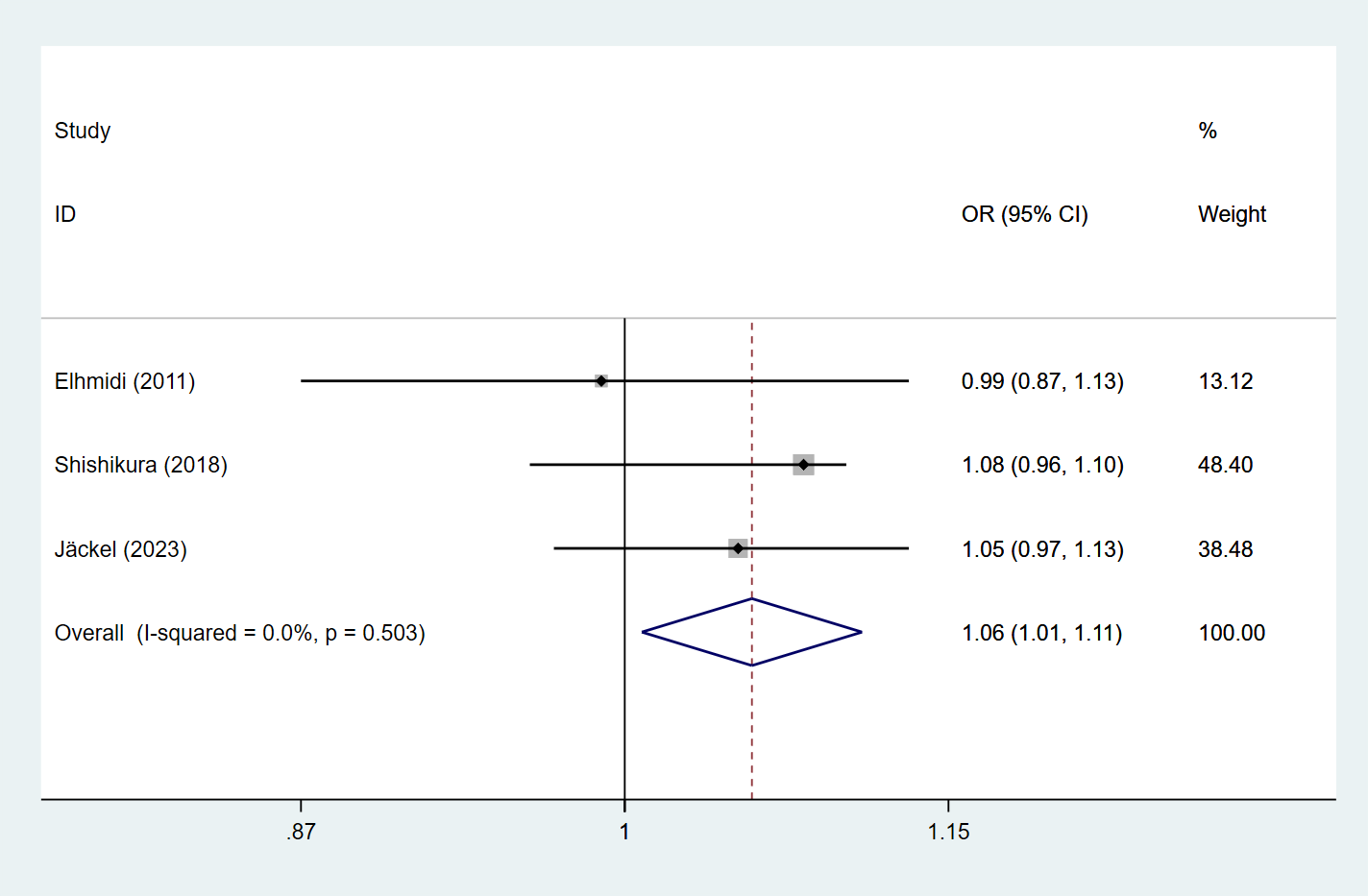


Figure S7 STS score Multivariable Forest Plot


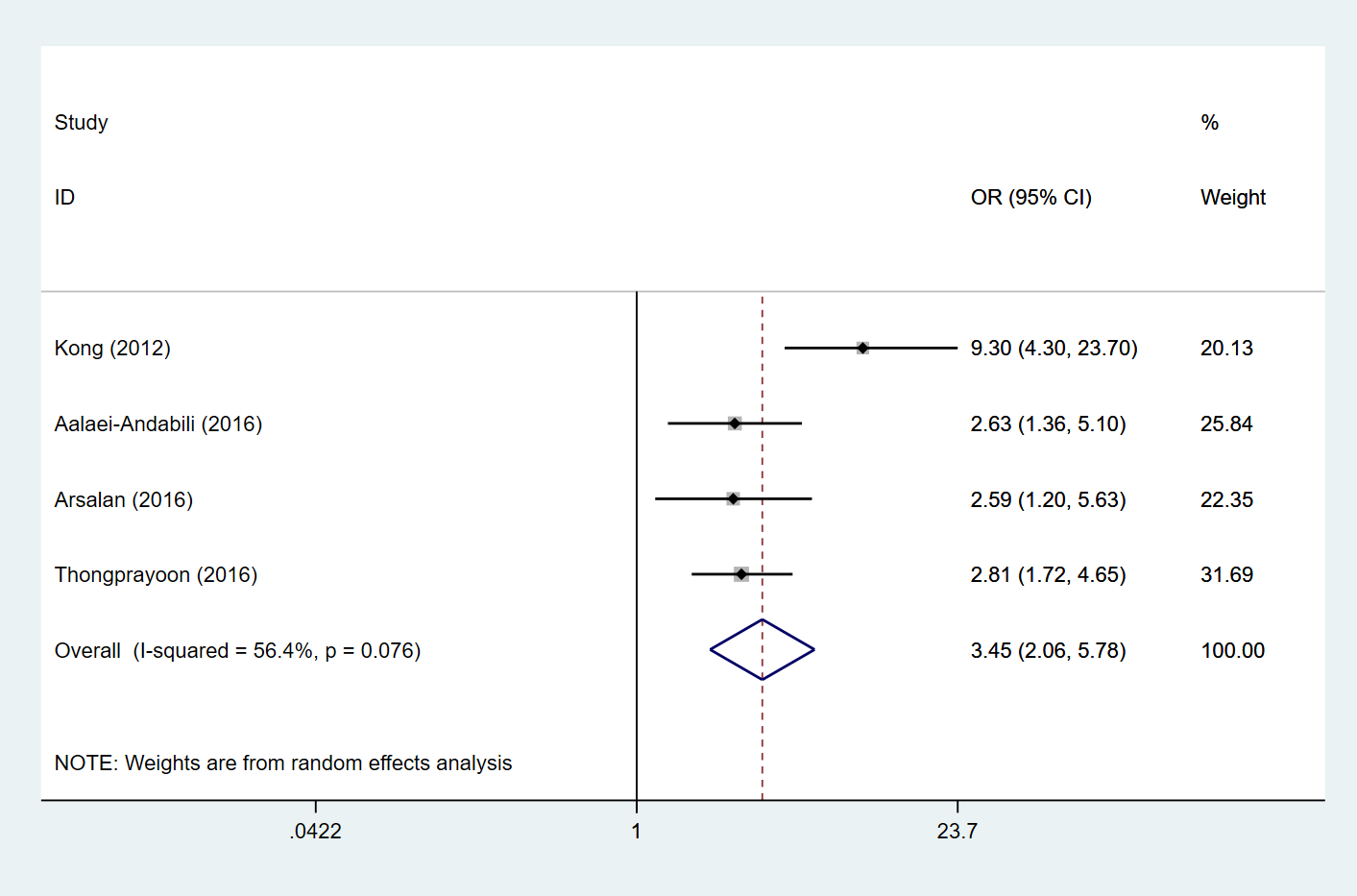


Figure S8 Transapical Access Multivariable Forest Plot


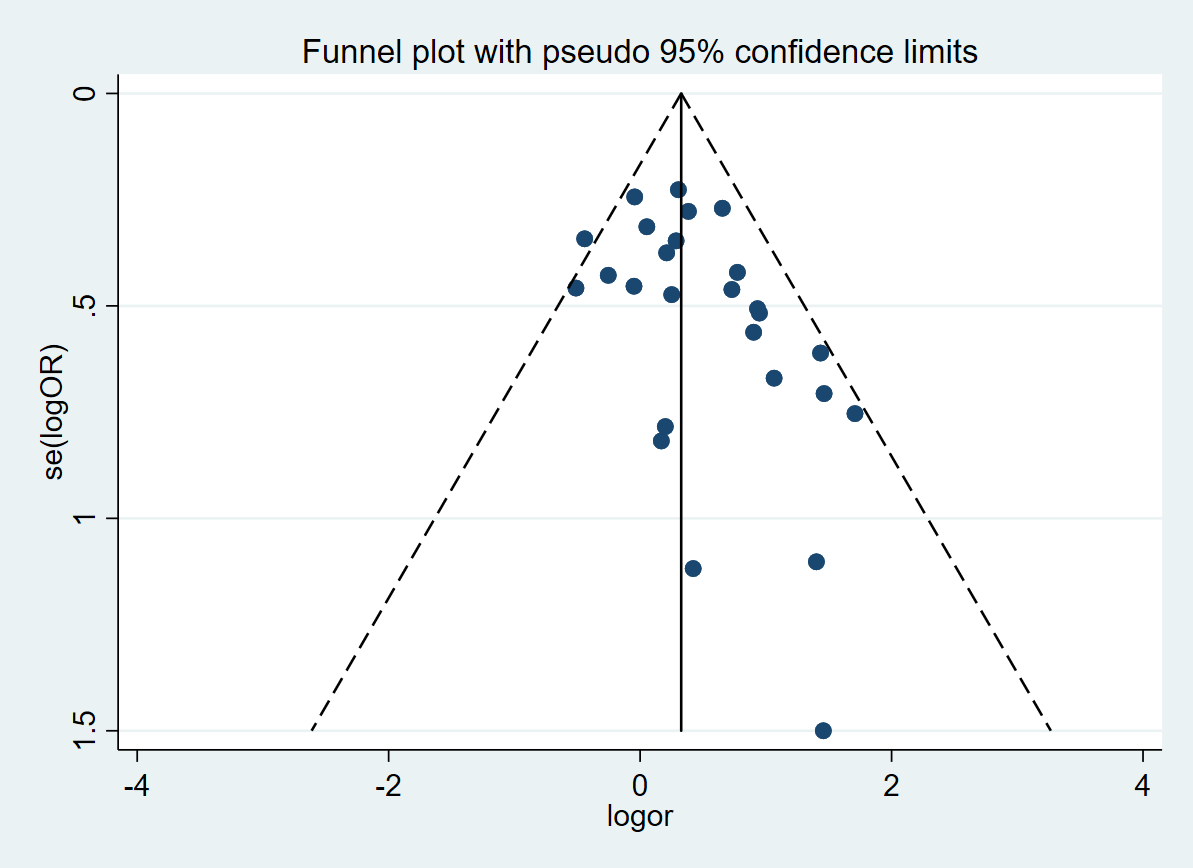


Figure S9 Hypertension Univariate Funnel Plotegger; Egger's Test P=0.025


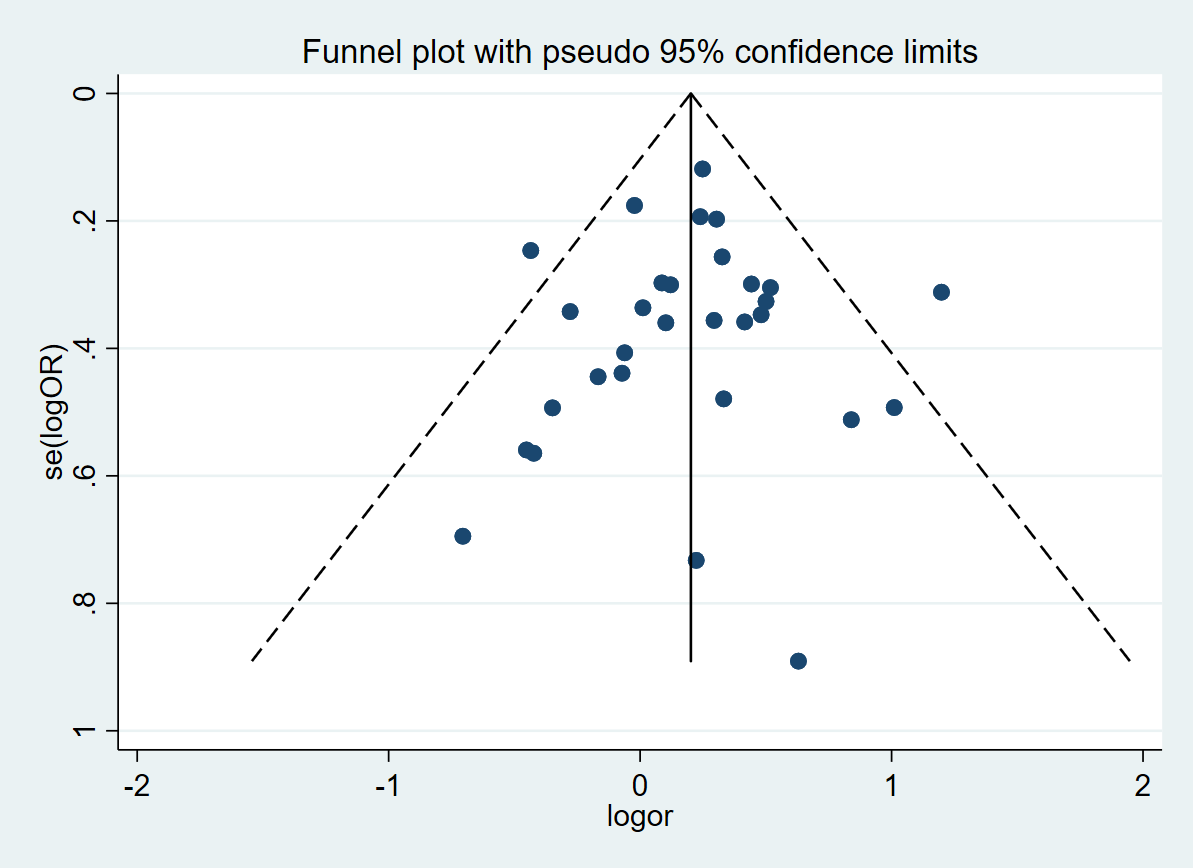


Figure S10 Diabetes Univariate Funnel Plotegger；Egger's Test P=0.801


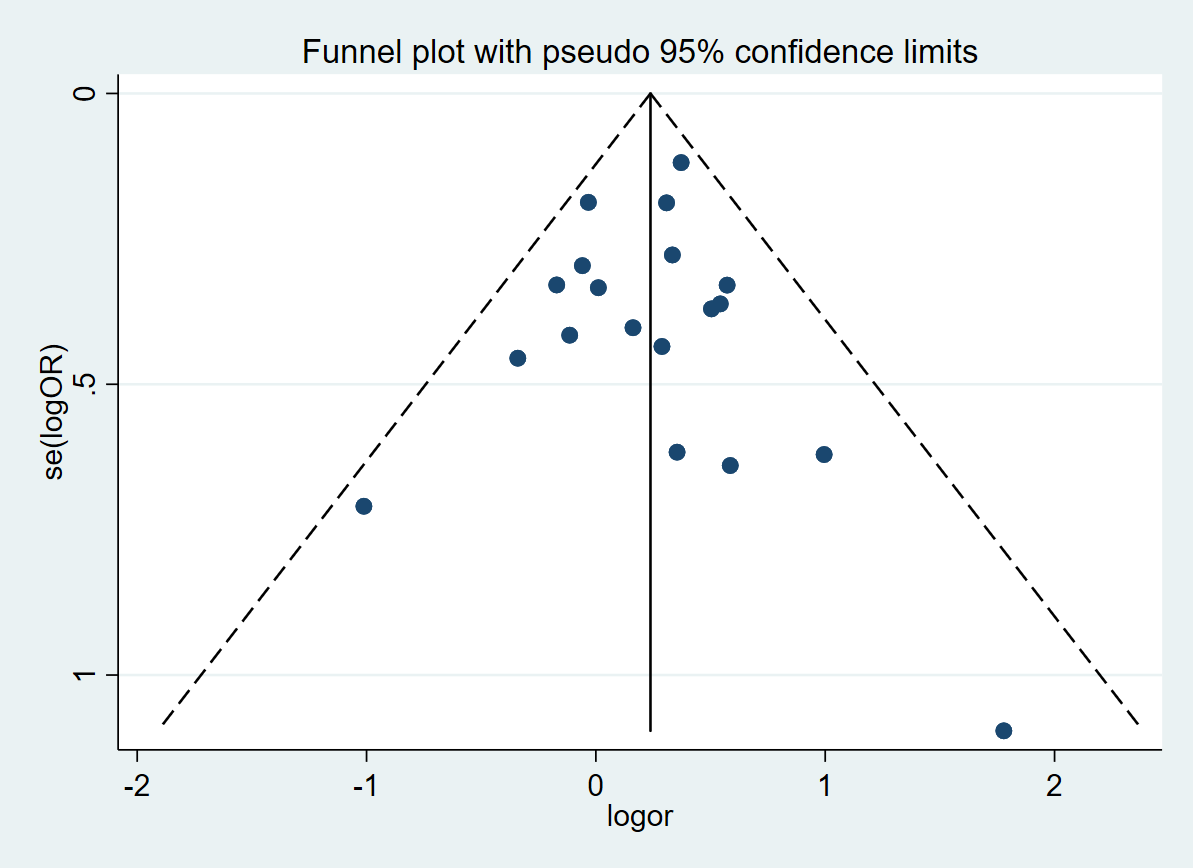


Figure S11 Coronary artery disease Univariate Funnel Plotegger；Egger's Test P=0.808


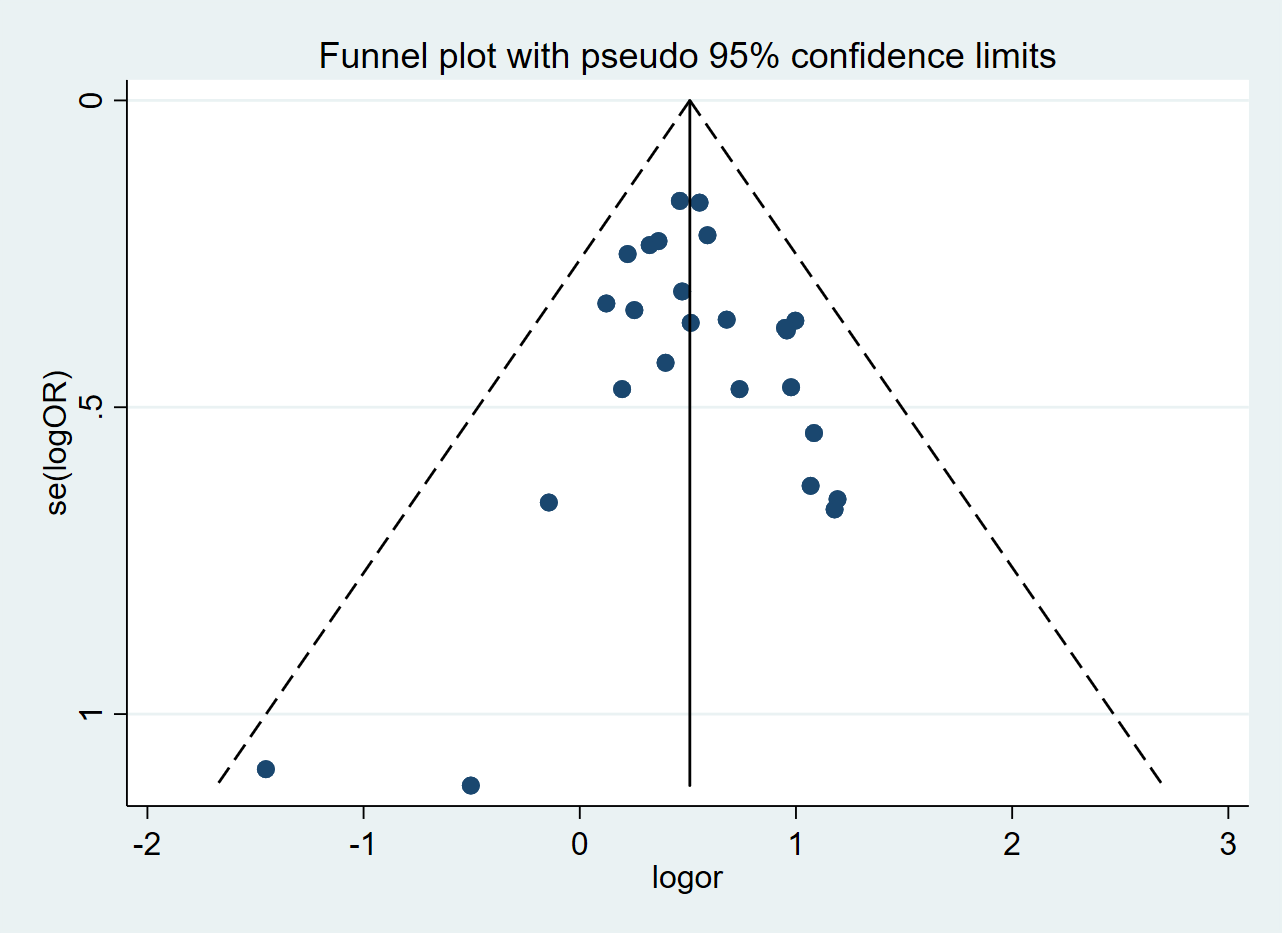


Figure S12 Peripheral Vascular Disease Univariate Funnel Plotegger；Egger's Test P=0.68


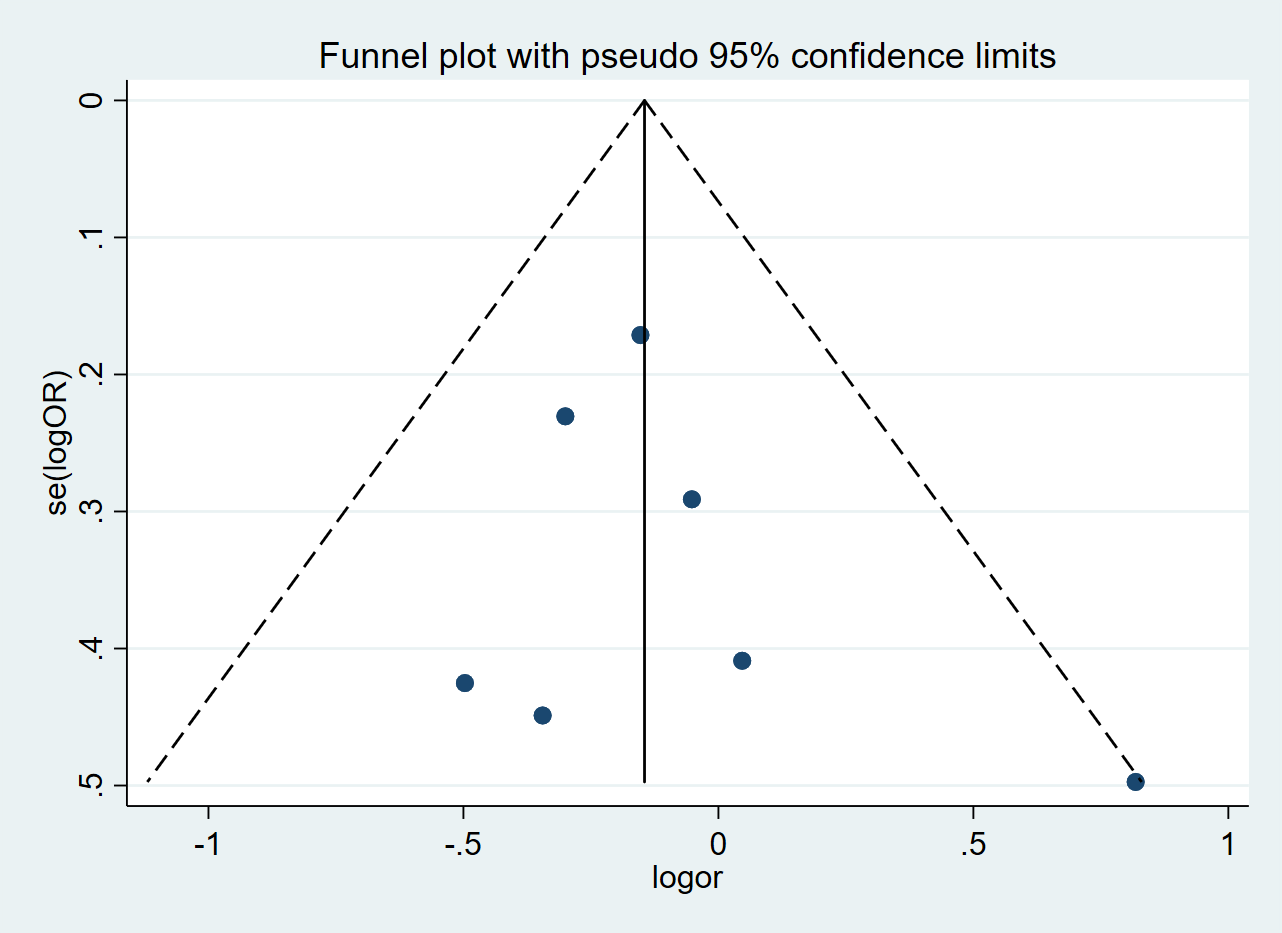


Figure S13 Porcelain aorta Univariate Funnel Plotegger；Egger's Test P=0.489


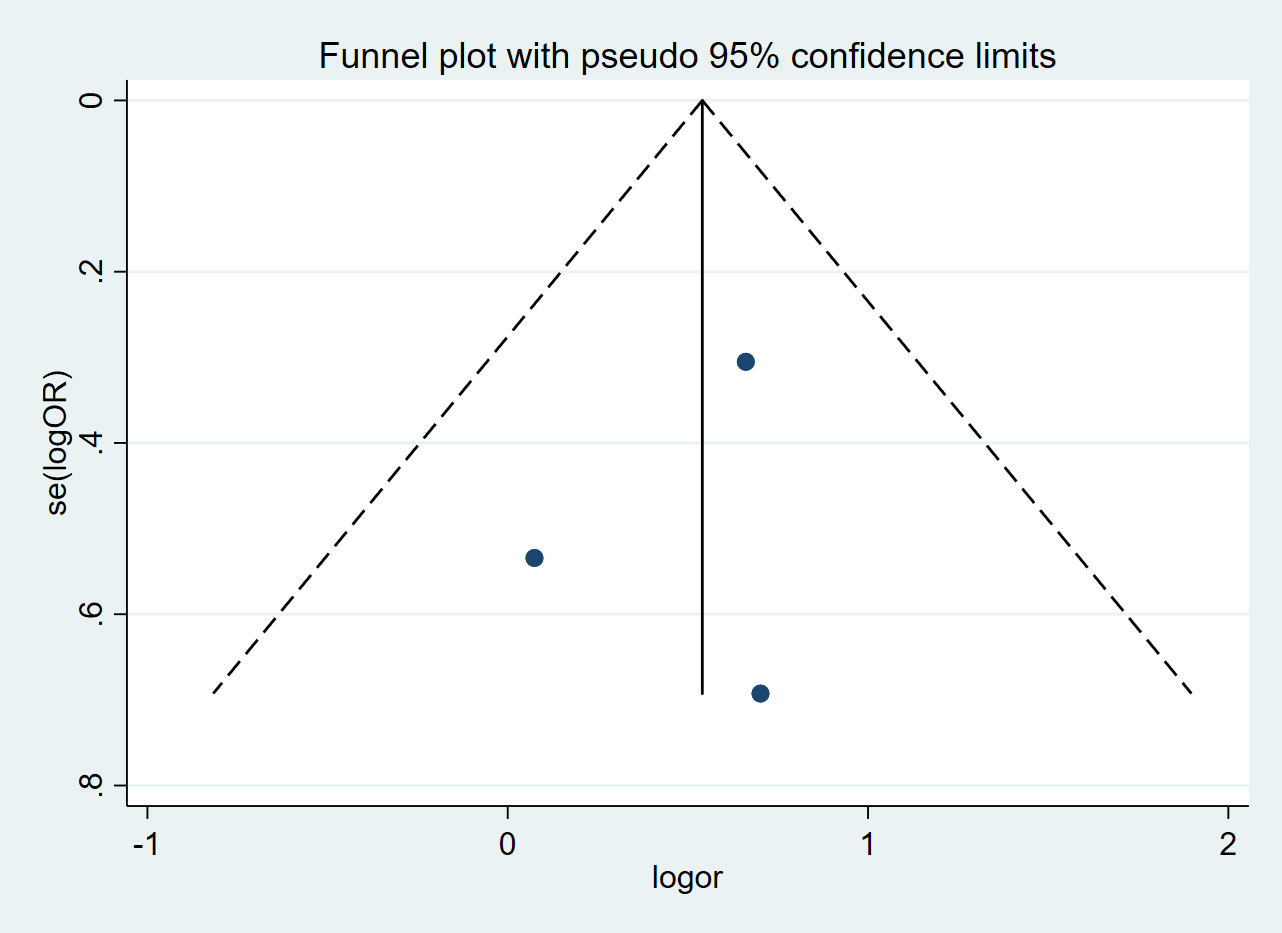


Figure S14 PCI for TAVR Univariate Funnel Plotegger；Egger's Test P=0.721


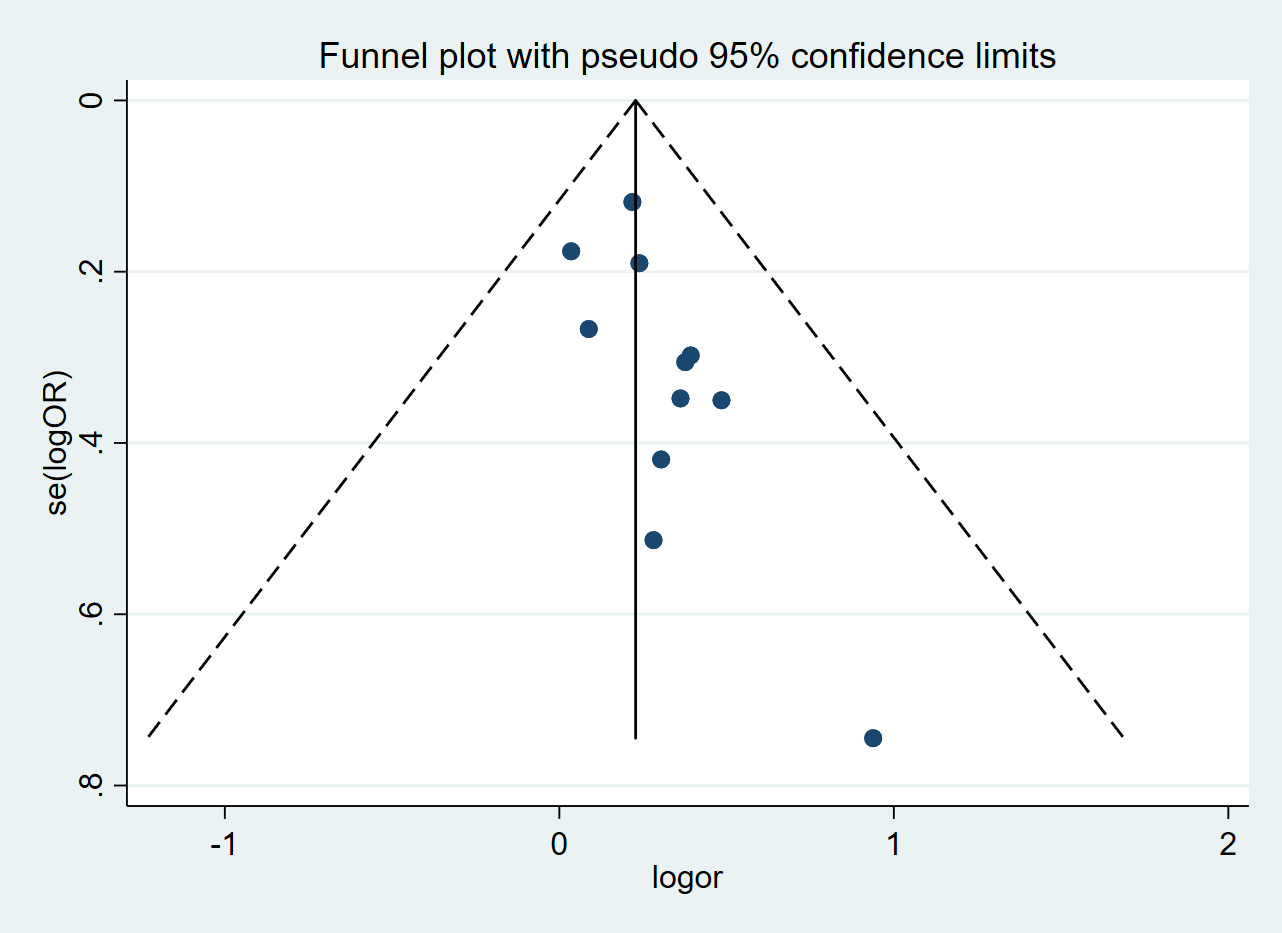


Figure S15 Atrial Fibrillation Univariate Funnel Plotegger；Egger's Test P=0.049


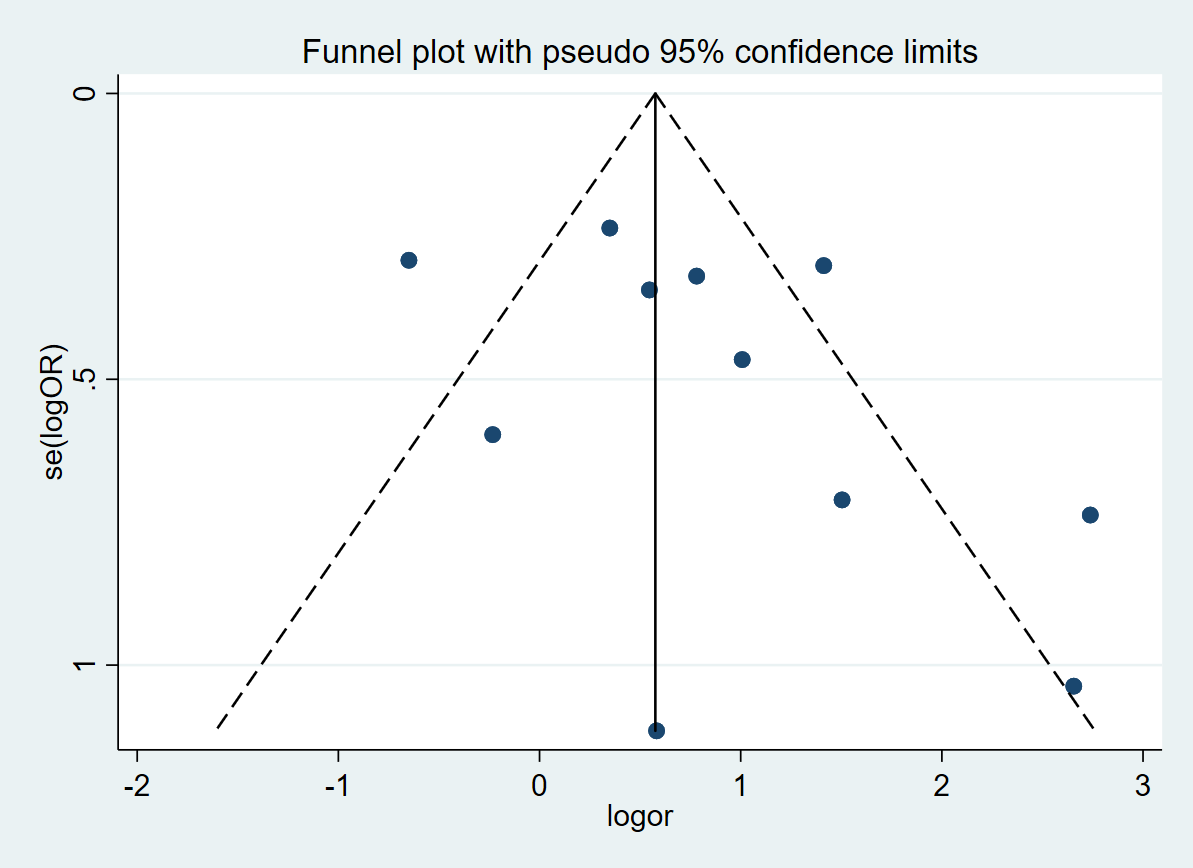


Figure S16 CKD Univariate Funnel Plotegger；Egger's Test P=0.188


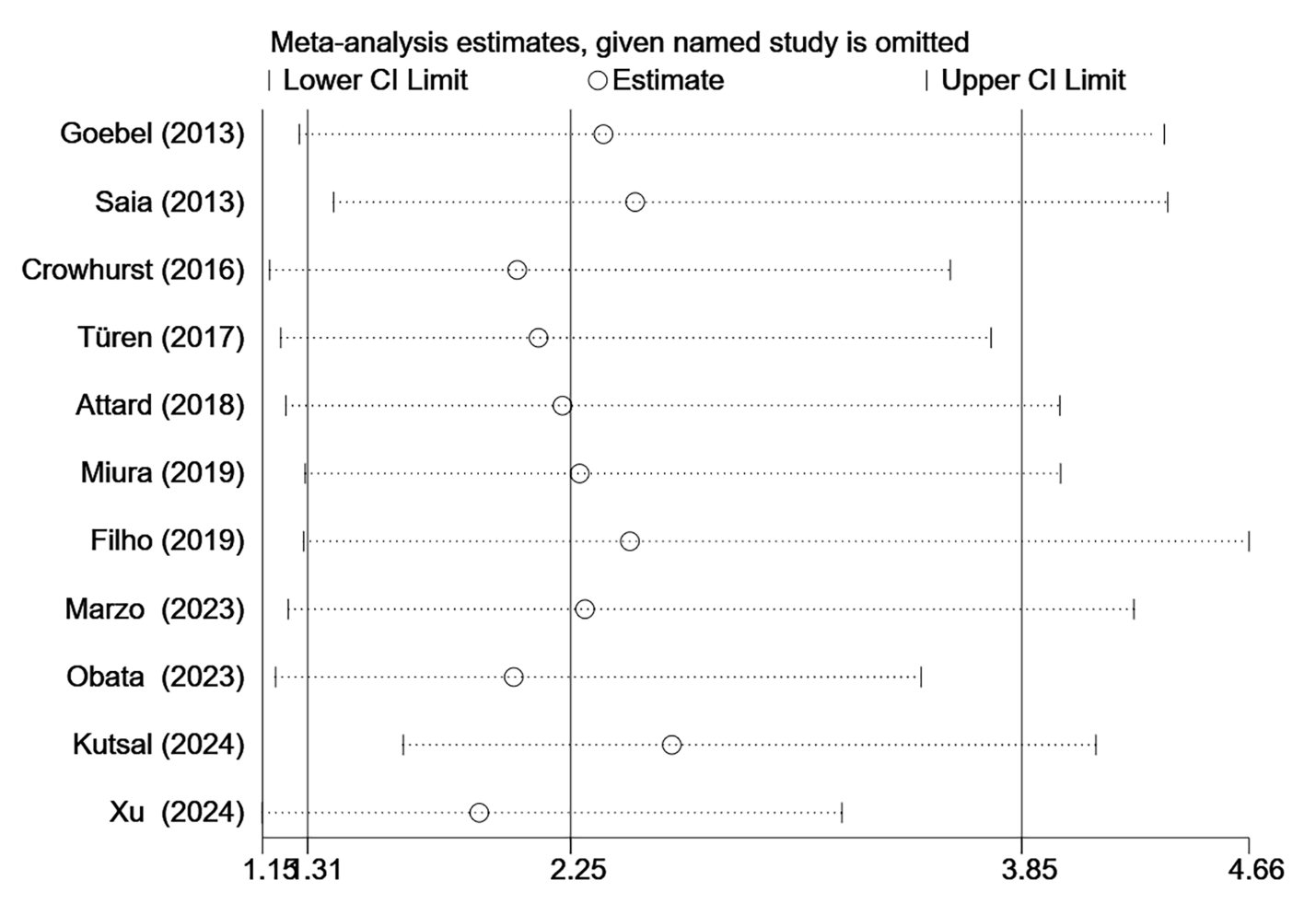


Figure S17 CKD Univariate Sensitivity Analysis


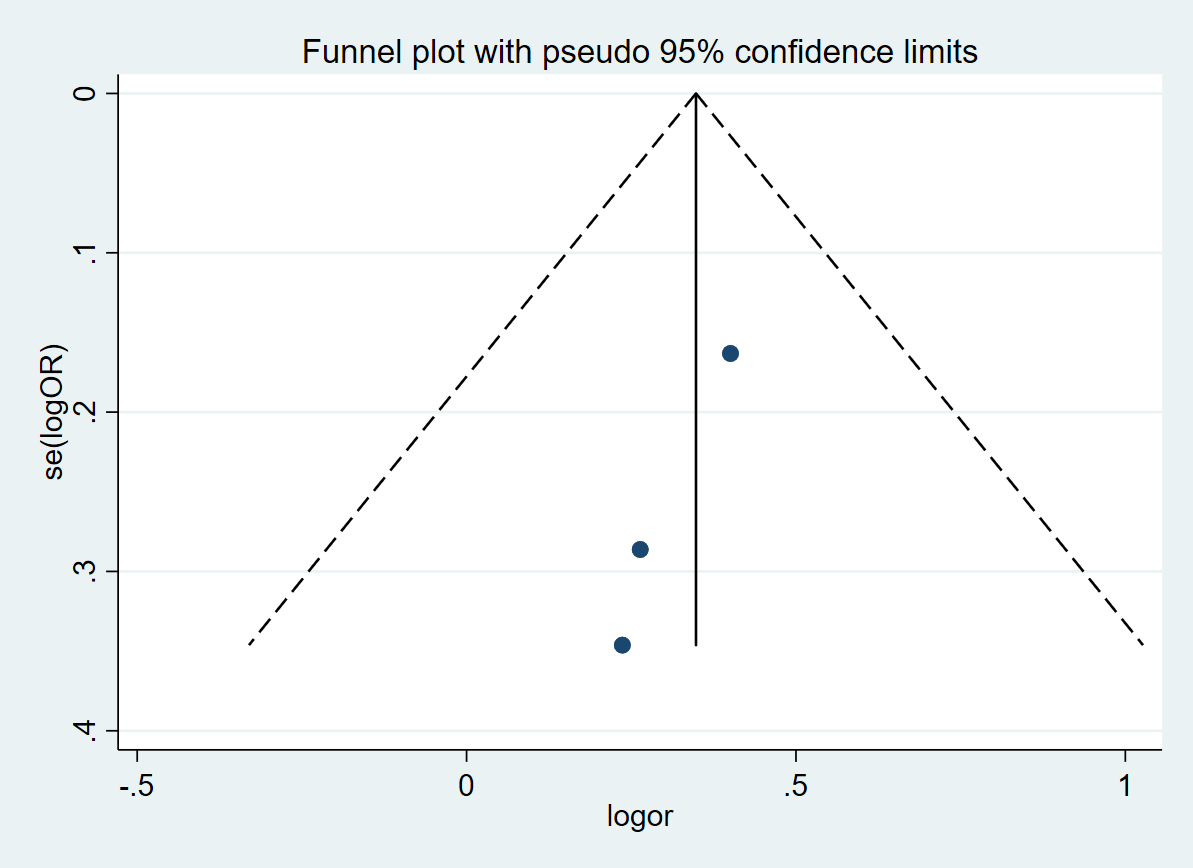


Figure S18 Congestive Heart Failure Univariate Funnel Plotegger；Egger's Test P=0.084


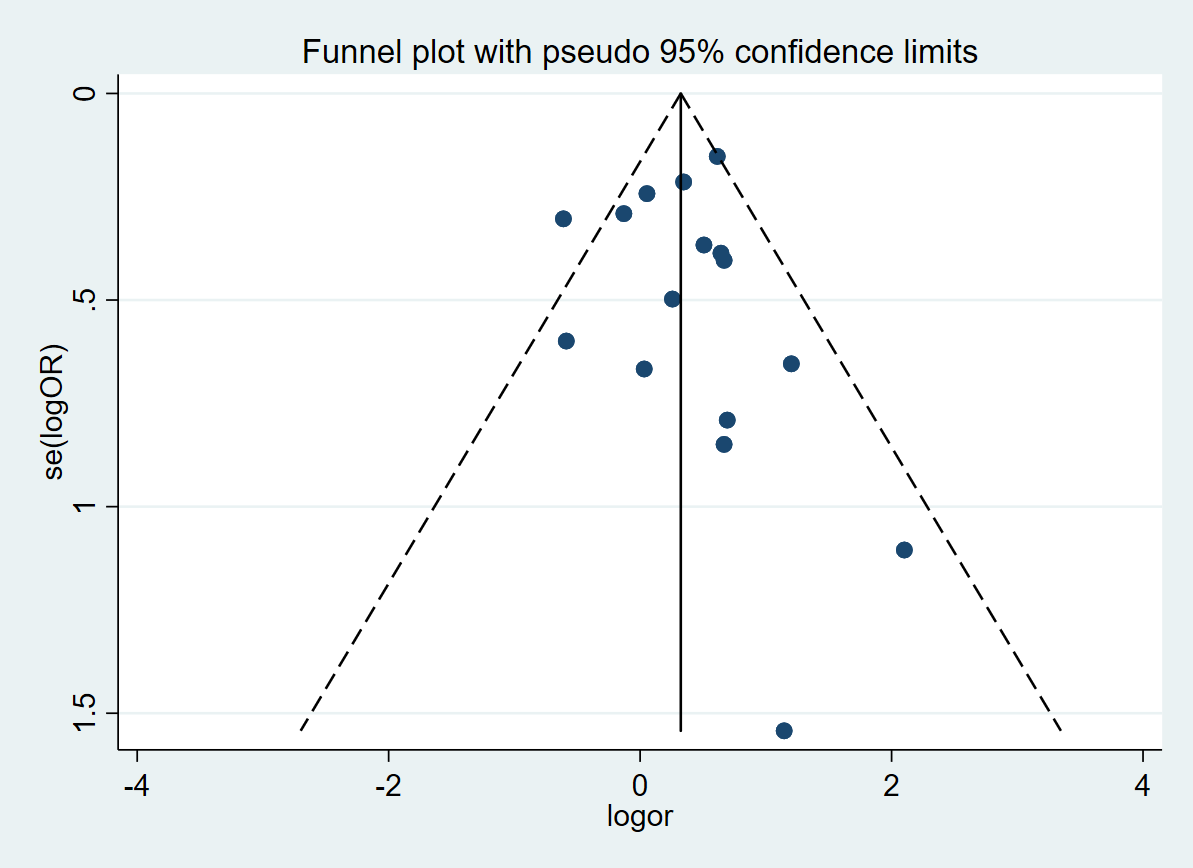


Figure S19 NYHA Class III–IV Univariate Funnel Plotegger；Egger's Test P=0.082


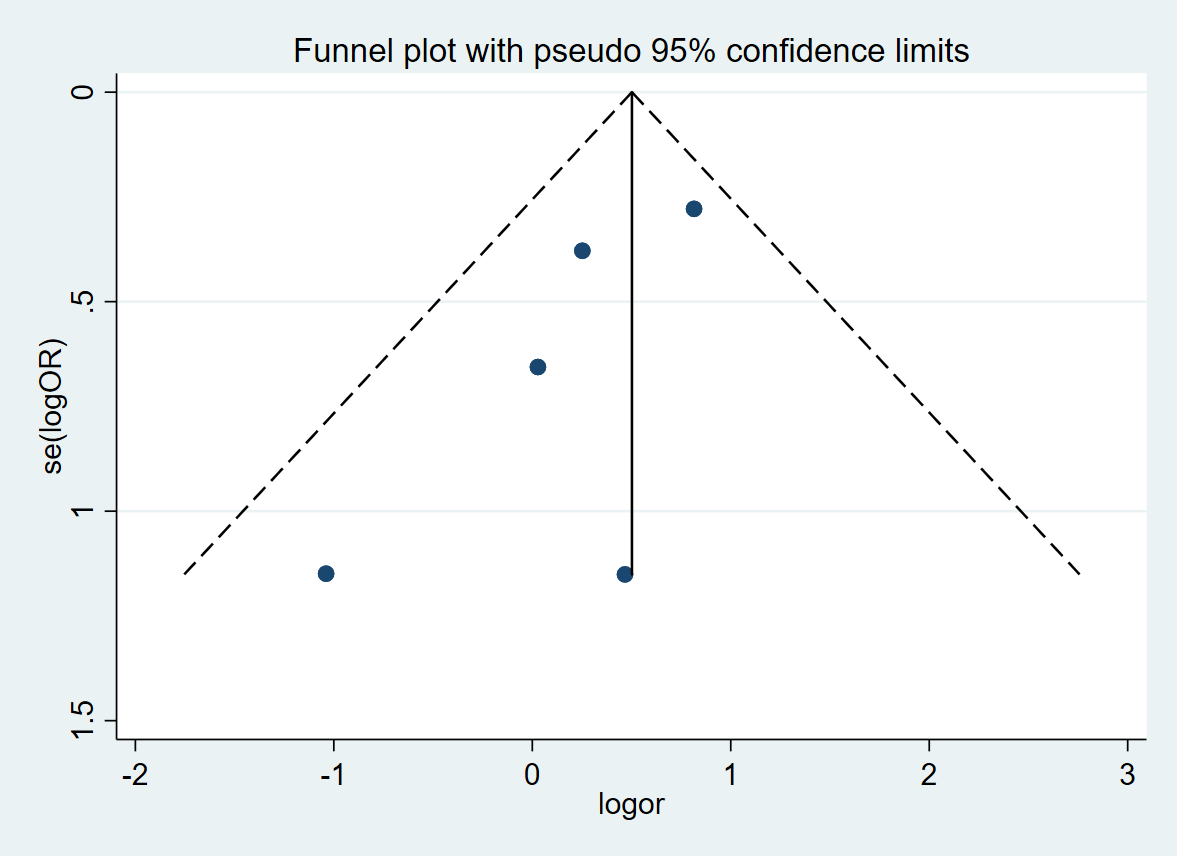


Figure S20 LVEF less than 40% Univariate Funnel Plotegger；Egger's Test P=0.15


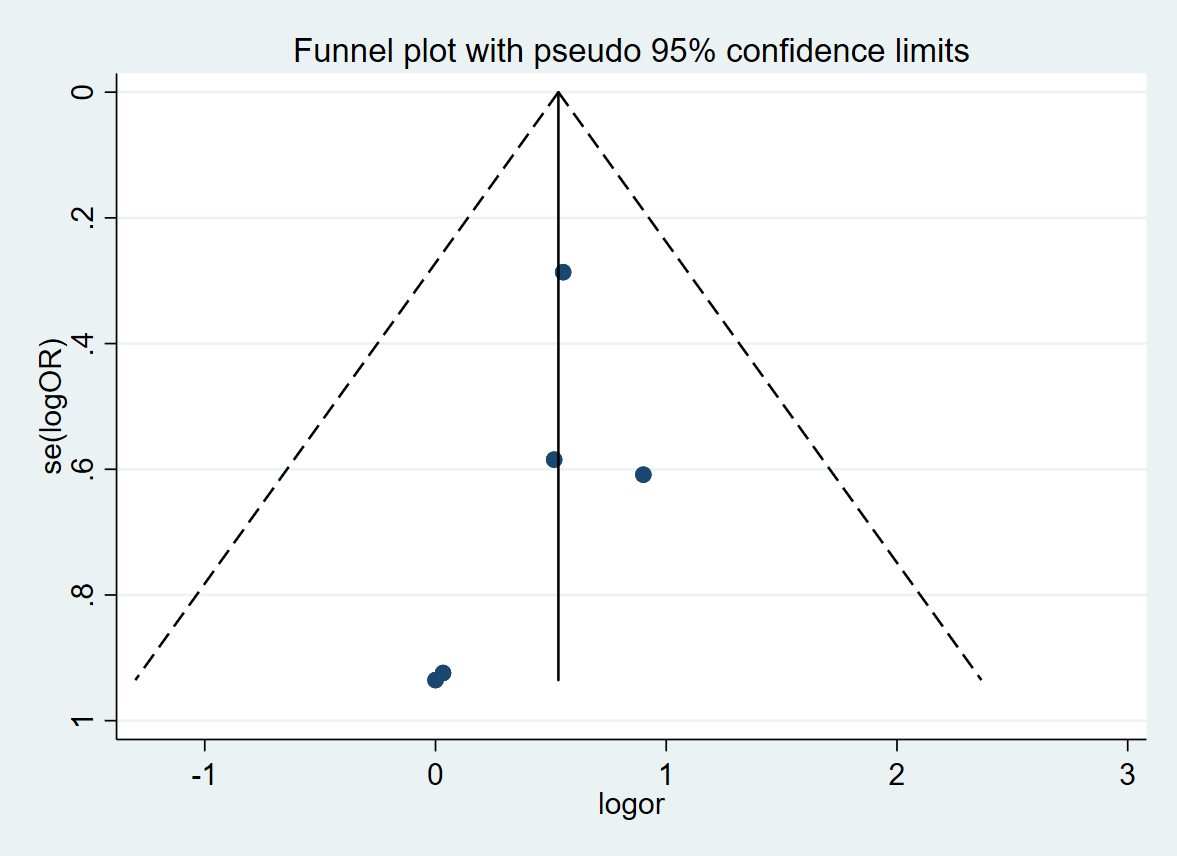


Figure S21 Postoperative Aortic Regurgitation more than Grade 2 Univariate Funnel Plotegger；Egger's Test P=0.423


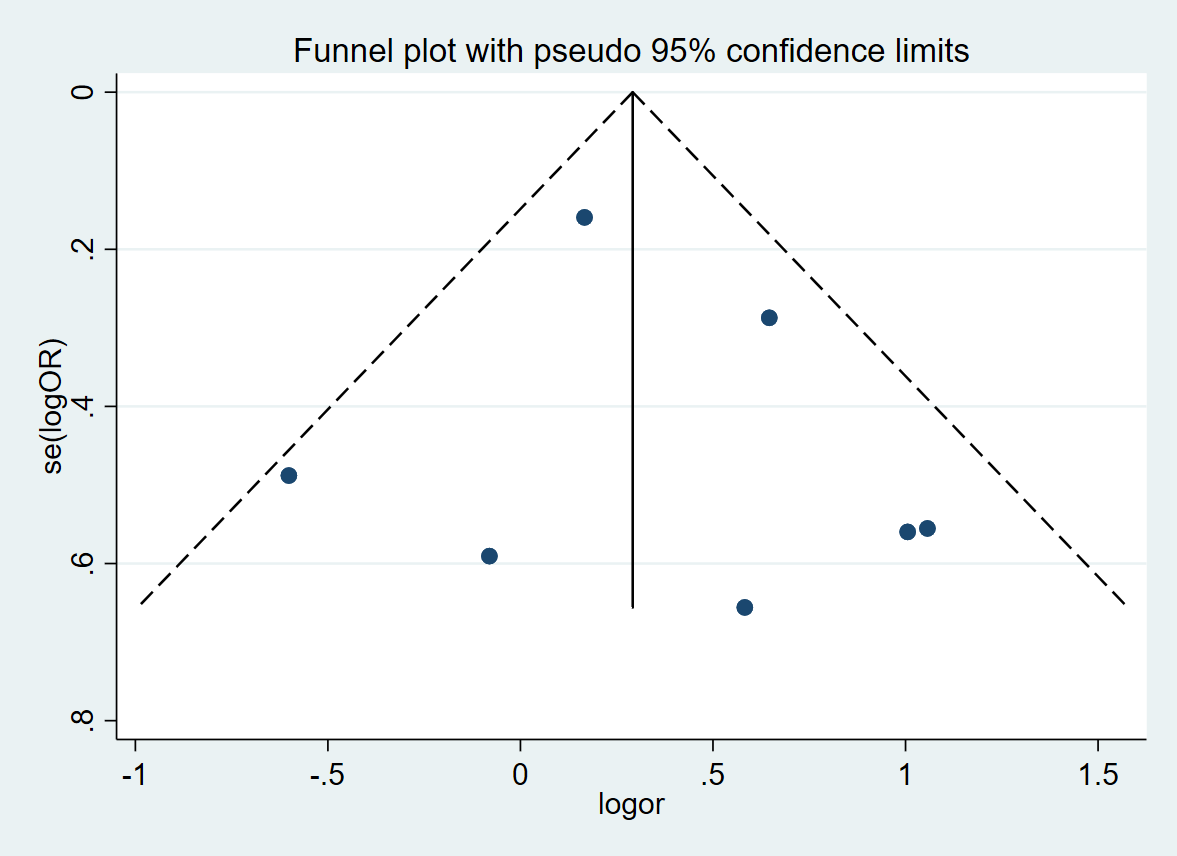


Figure S22 Preoperative Anemia Univariate Funnel Plotegger；Egger's Test P=0.577


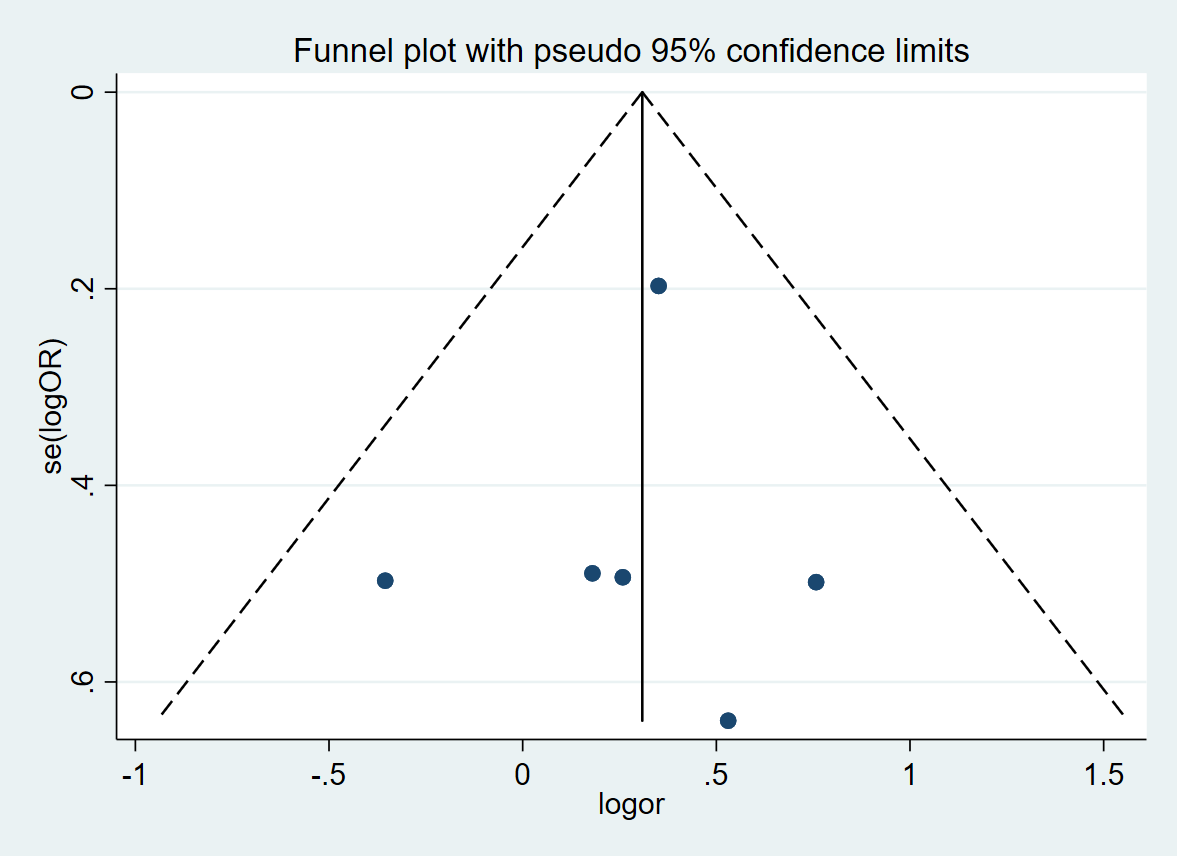


Figure S23 Diuretic Use Univariate Funnel Plotegger；Egger's Test P=0.8


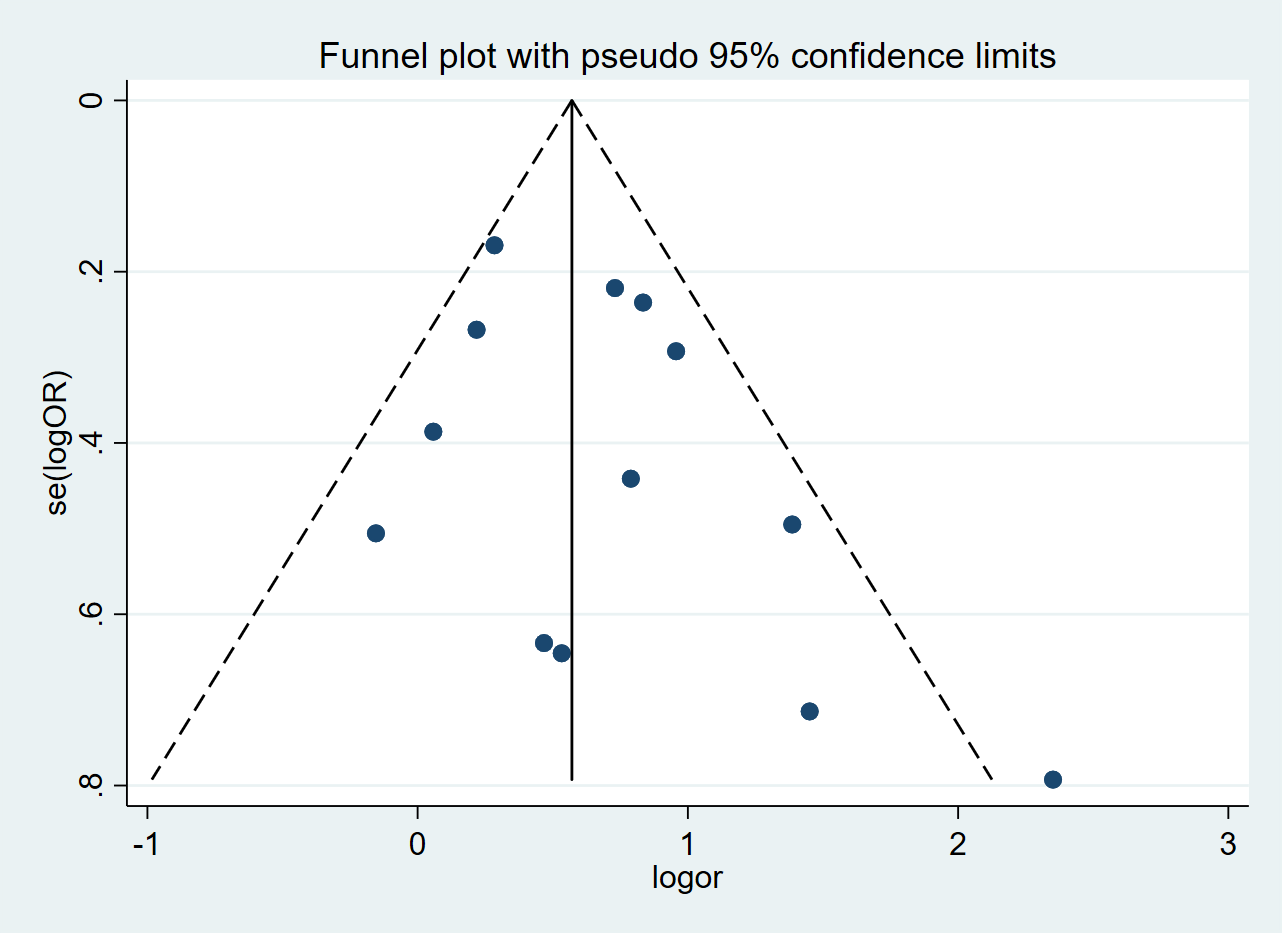


Figure S24 Transapical Access Univariate Funnel Plotegger；Egger's Test P=0.206


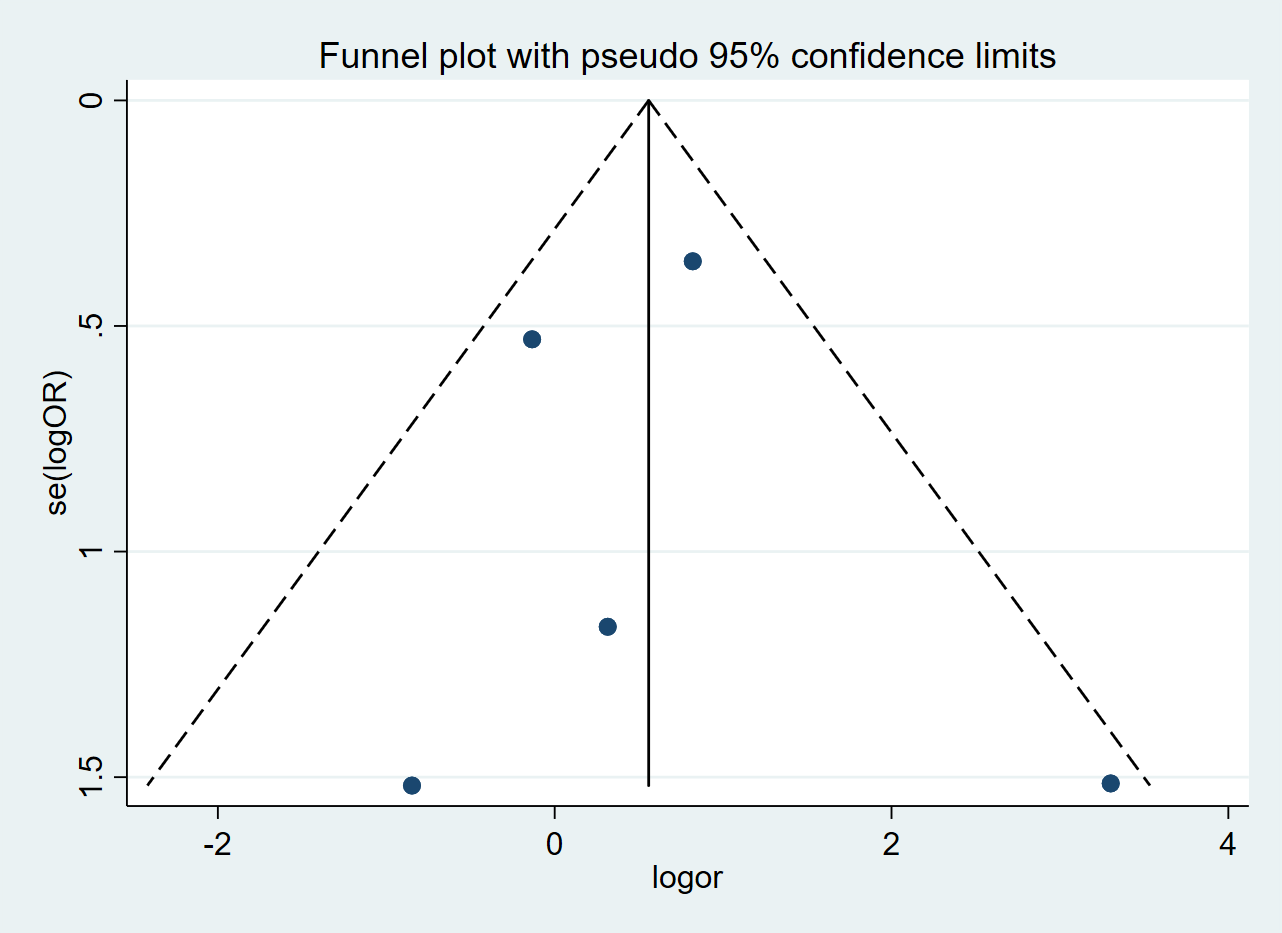


Figure S25 Transaortic Access Univariate Funnel Plotegger；Egger's Test P=0.955


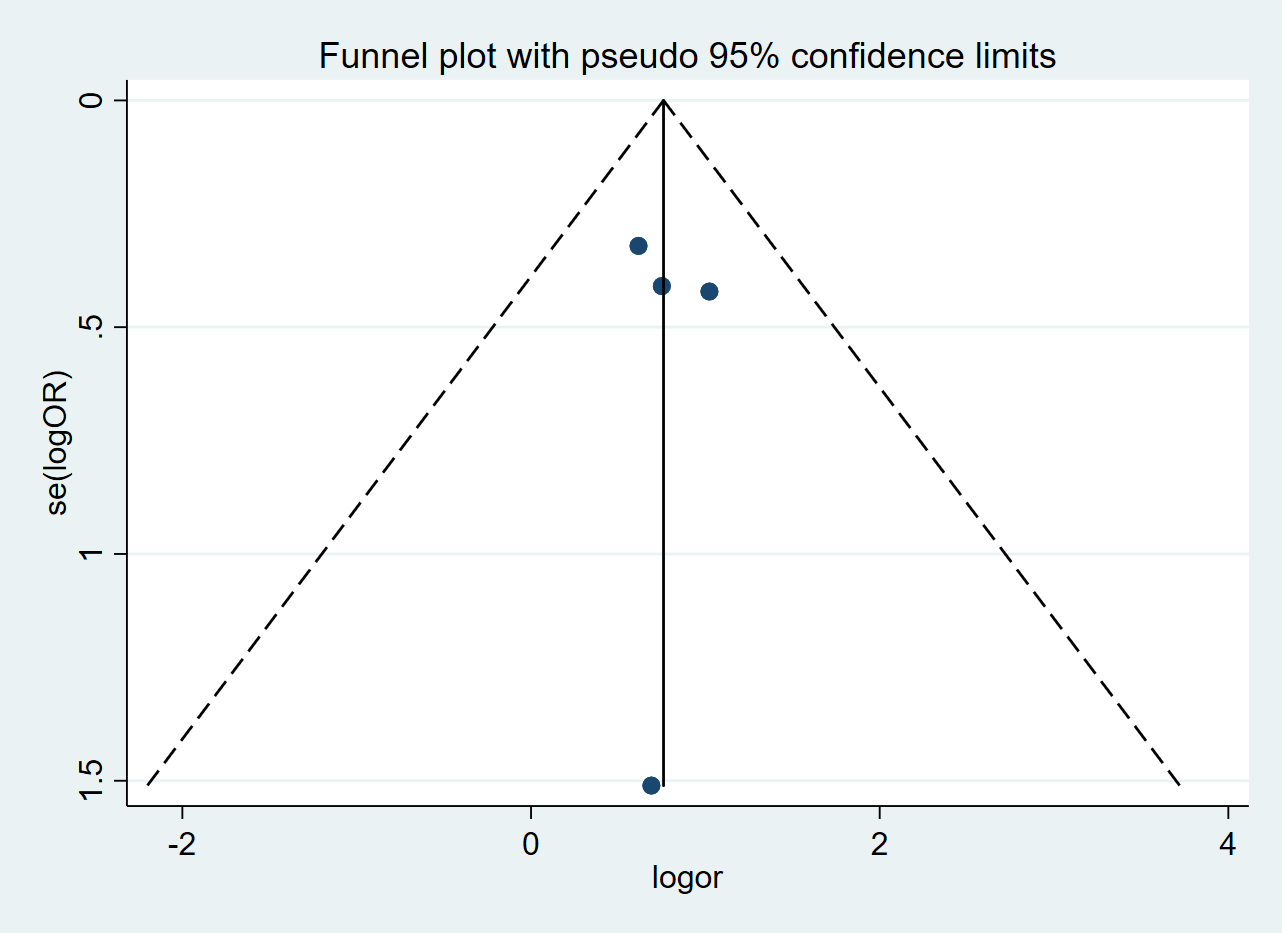


Figure S26 General Anesthesia Univariate Funnel Plotegger；Egger's Test P=0.82


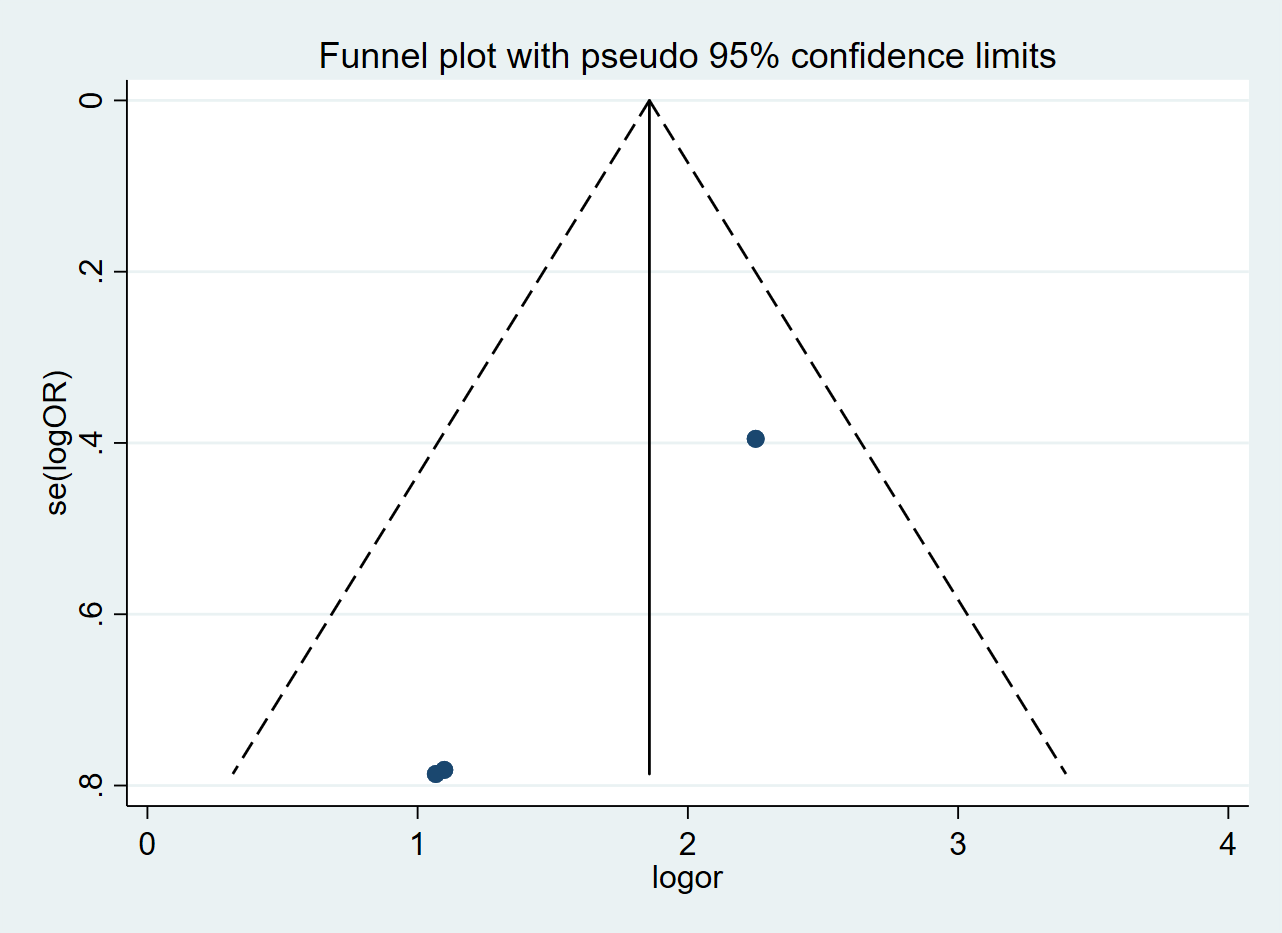


Figure S27 Intraoperative Rapid Pacing  Univariate Funnel Plotegger；Egger's Test P=0.006


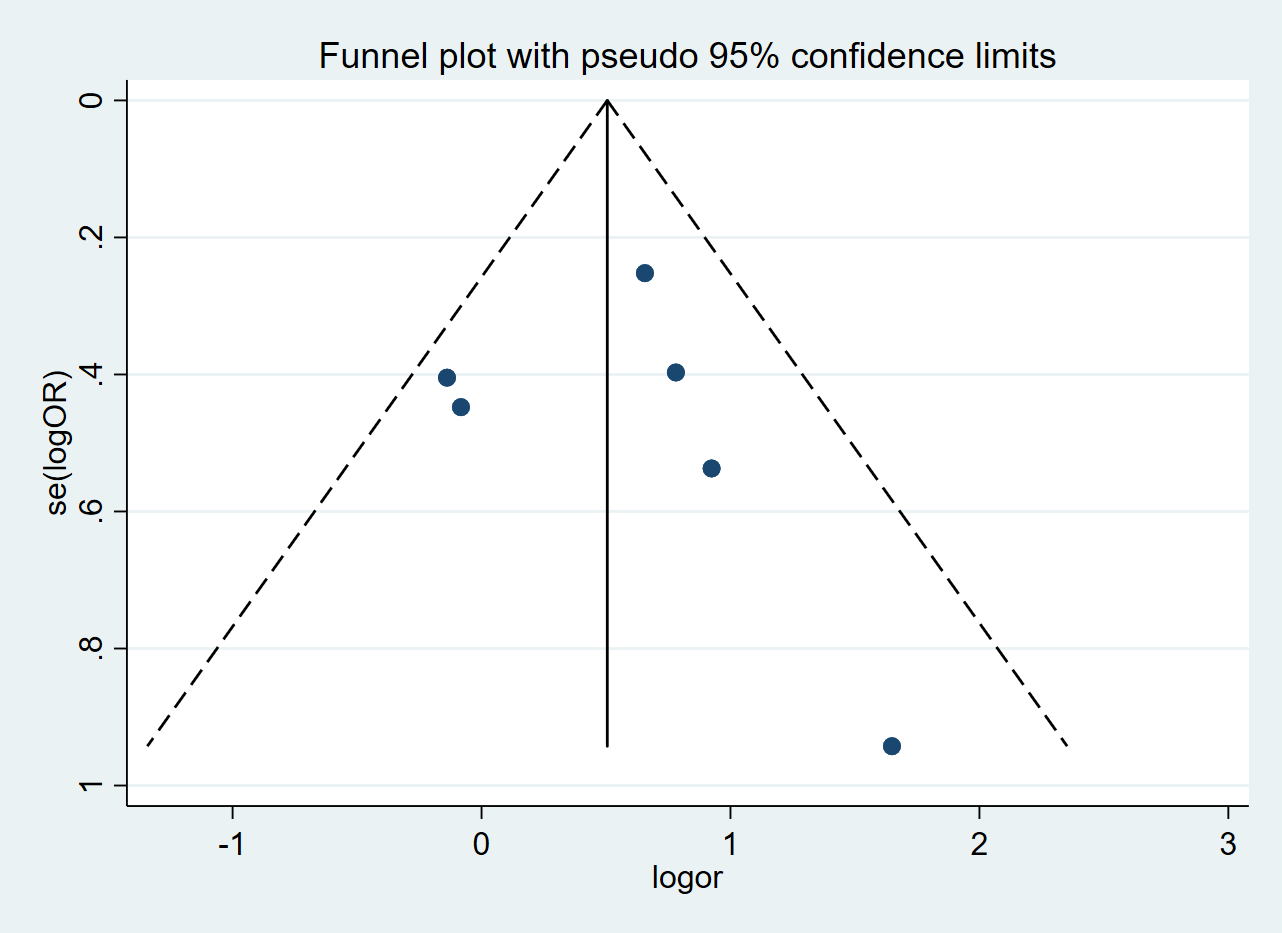


Figure S28 Vascular Complications Univariate Funnel Plotegger；Egger's Test P=0.751


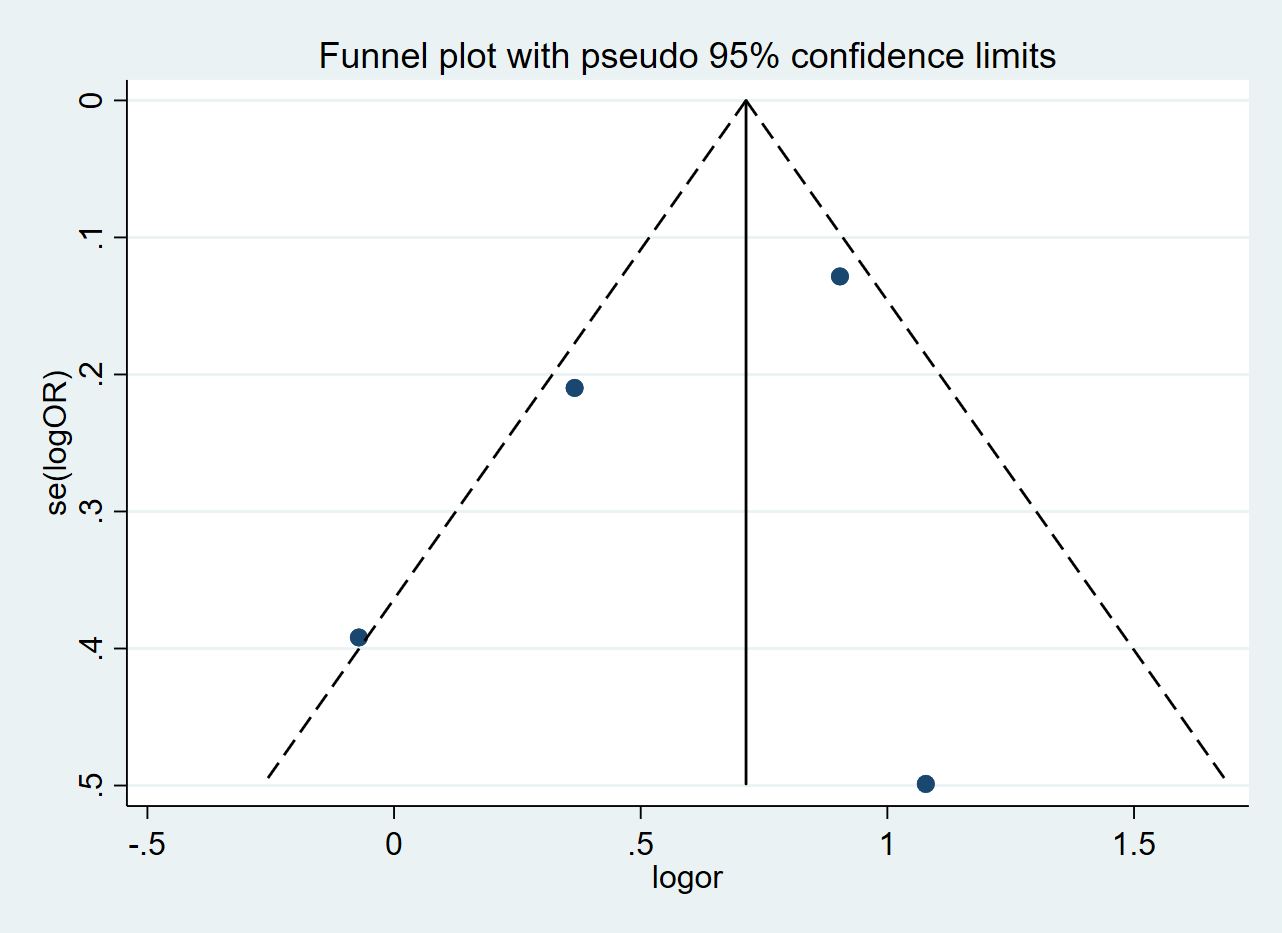


Figure S29 Bleeding Complications Univariate Funnel Plotegger；Egger's Test P=0.5


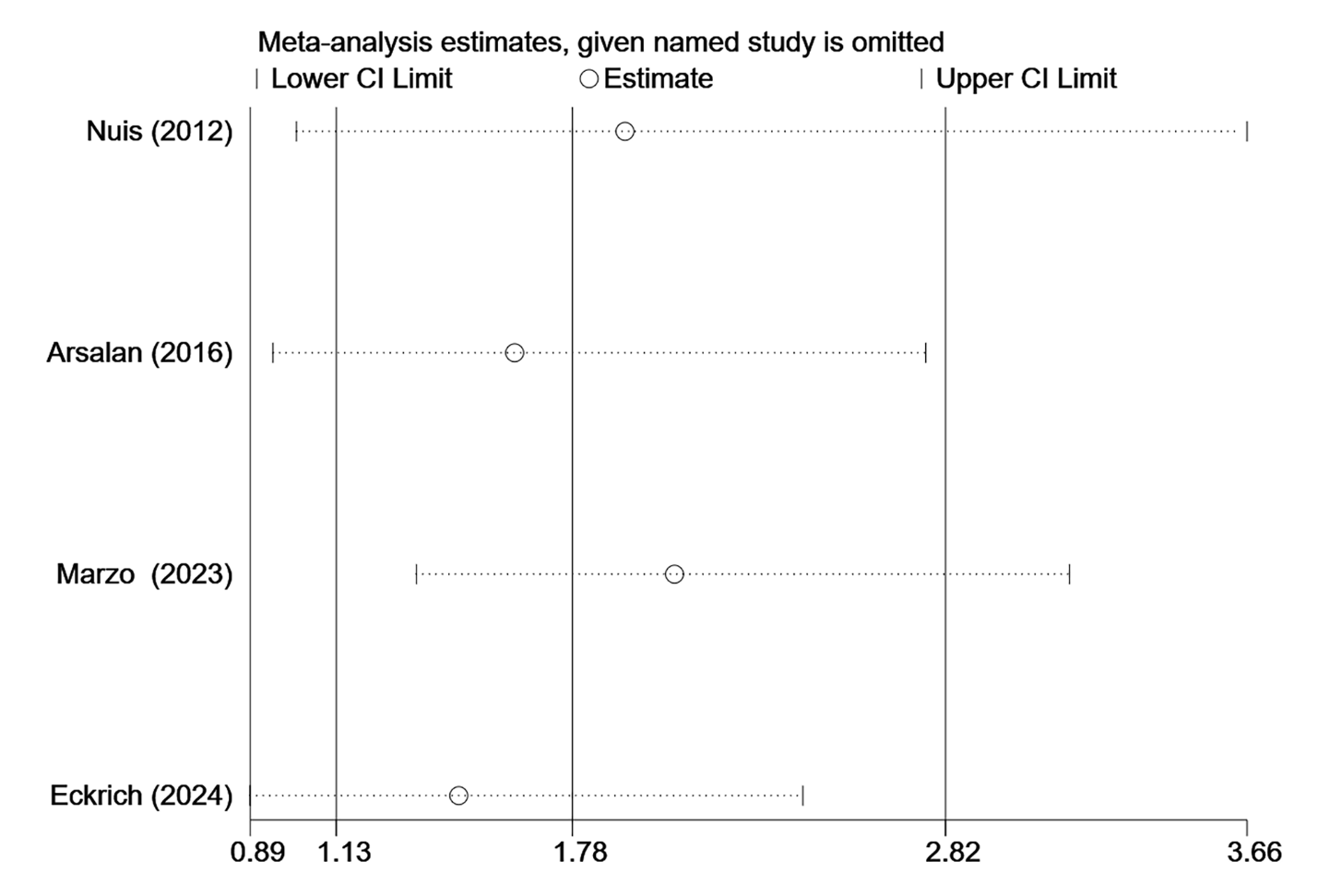


Figure S30 Bleeding Complications Univariate Sensitivity Analysis


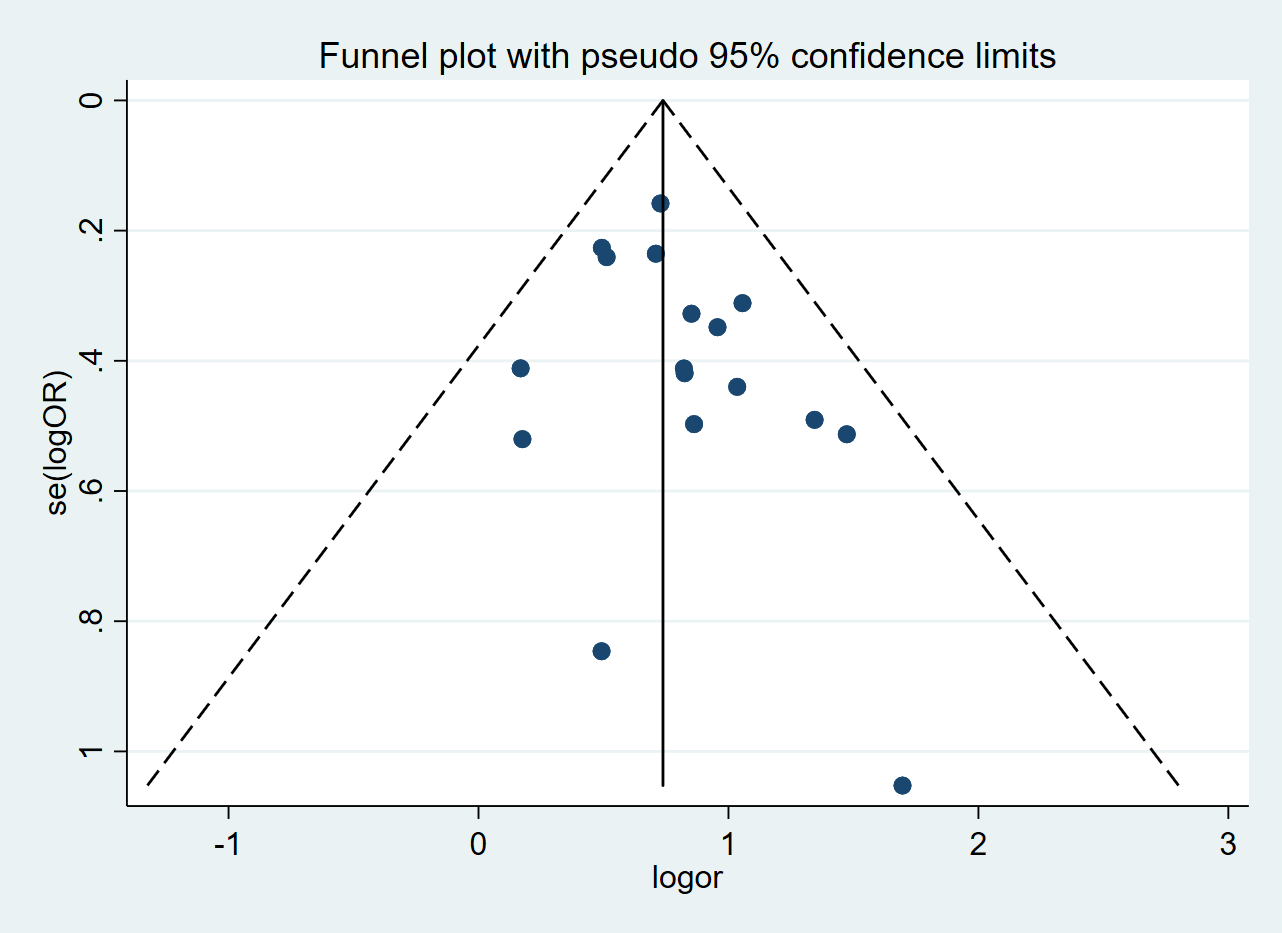


Figure S31 Blood Transfusion Univariate Funnel Plotegger；Egger's Test P=0.195


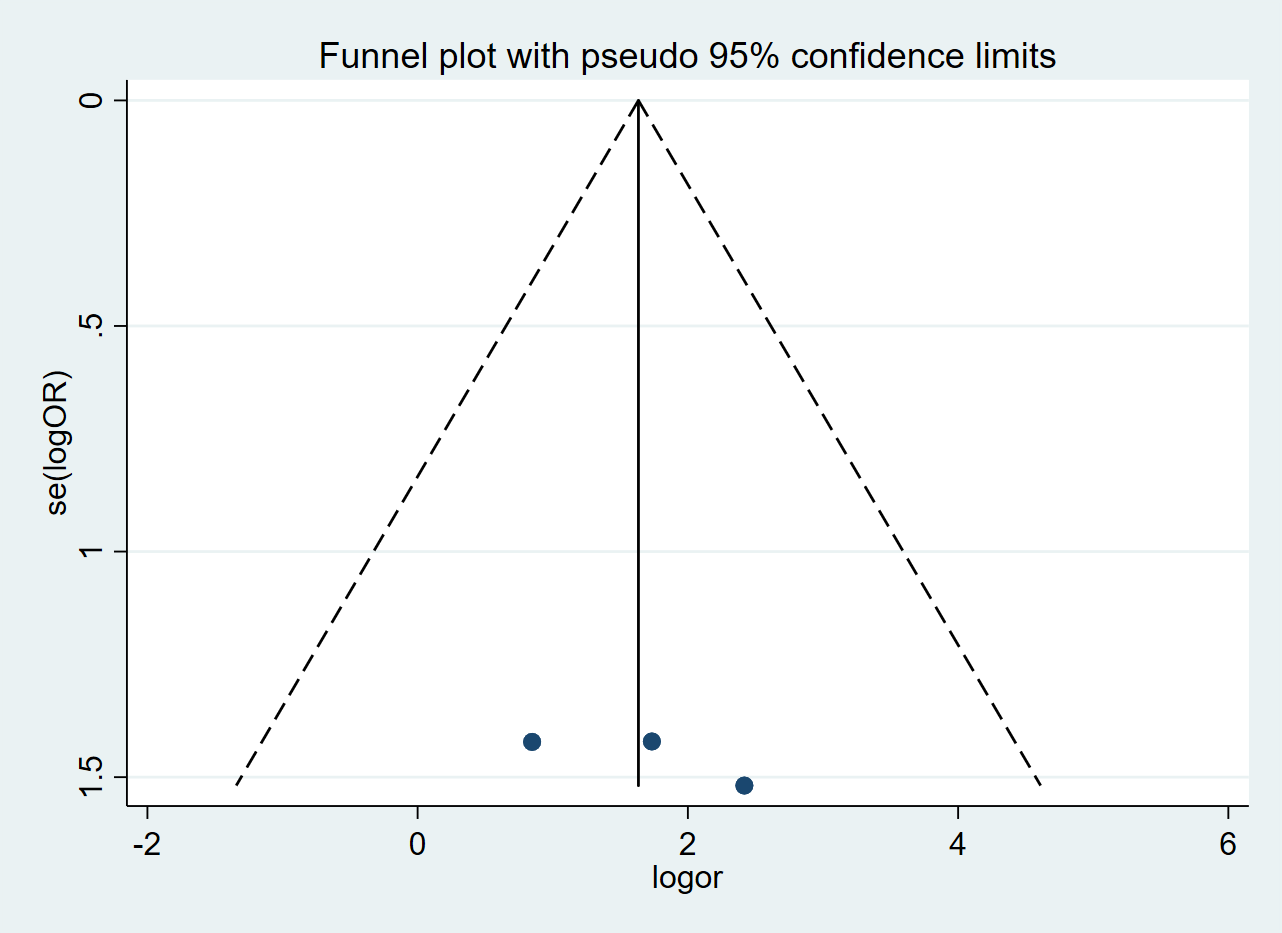


Figure S32 Postoperative MI Univariate Funnel Plotegger；Egger's Test P=0.4


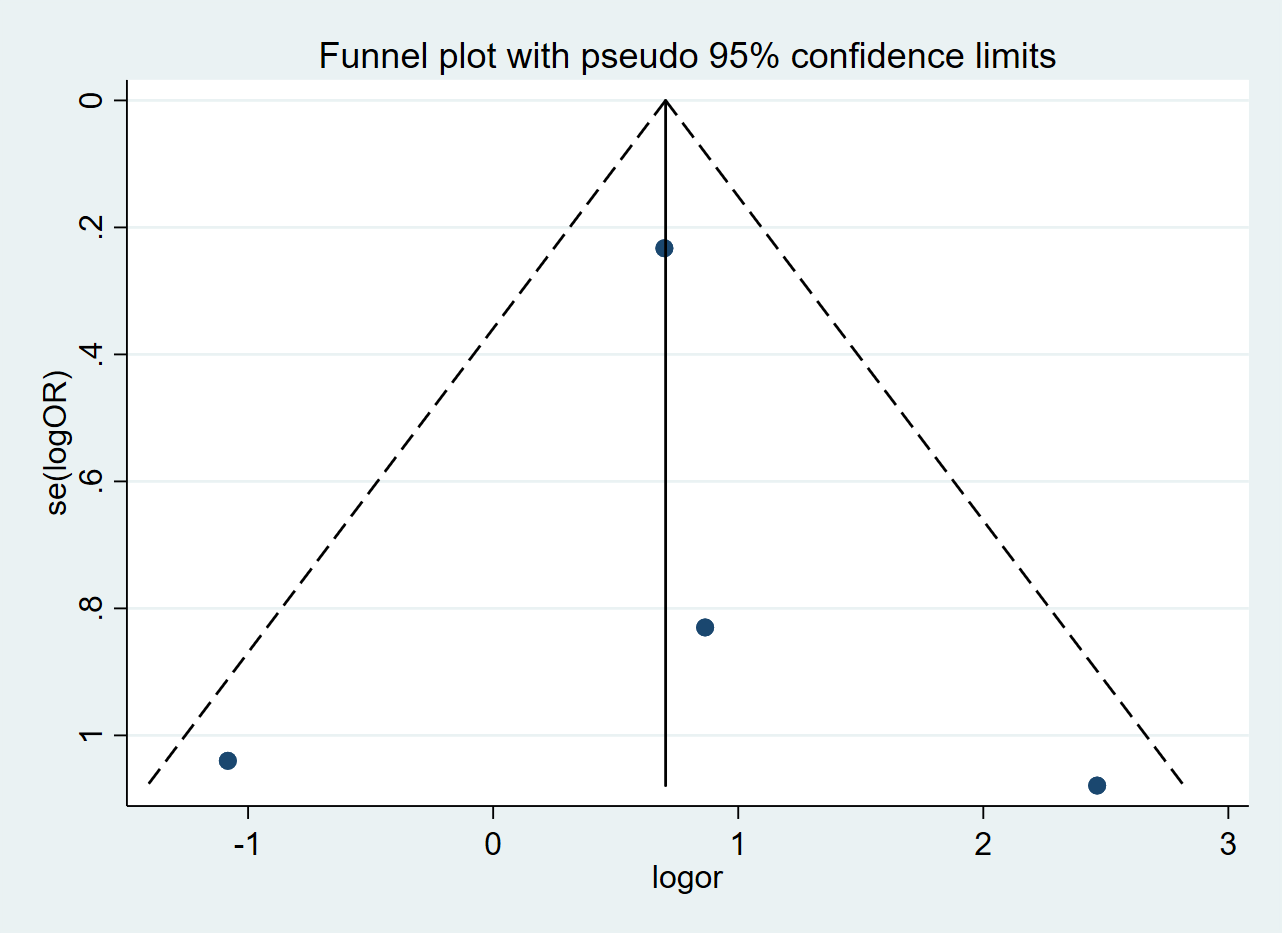


Figure S33 Postoperative Stroke Univariate Funnel Plotegger；Egger's Test P=0.97


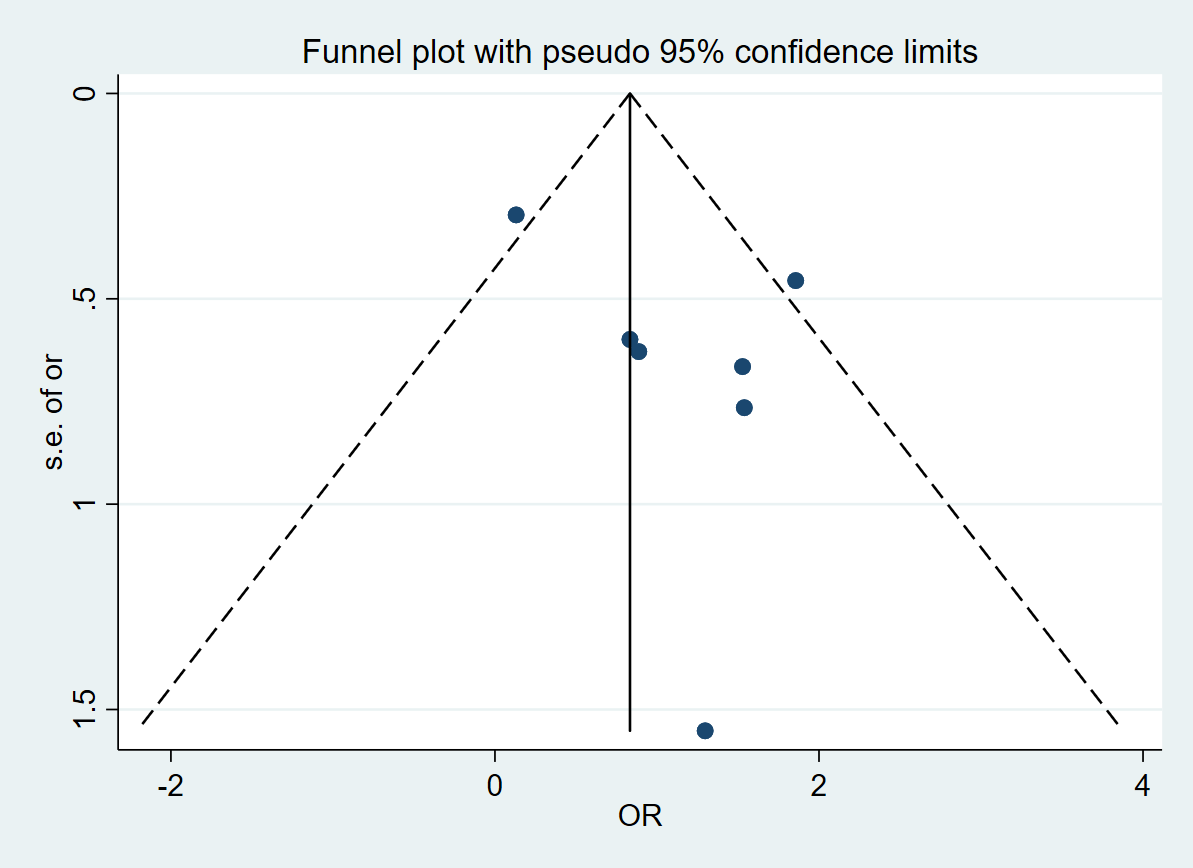


Figure S34 Hypertension Multivariable Funnel Plotegger；Egger's Test P=0.178


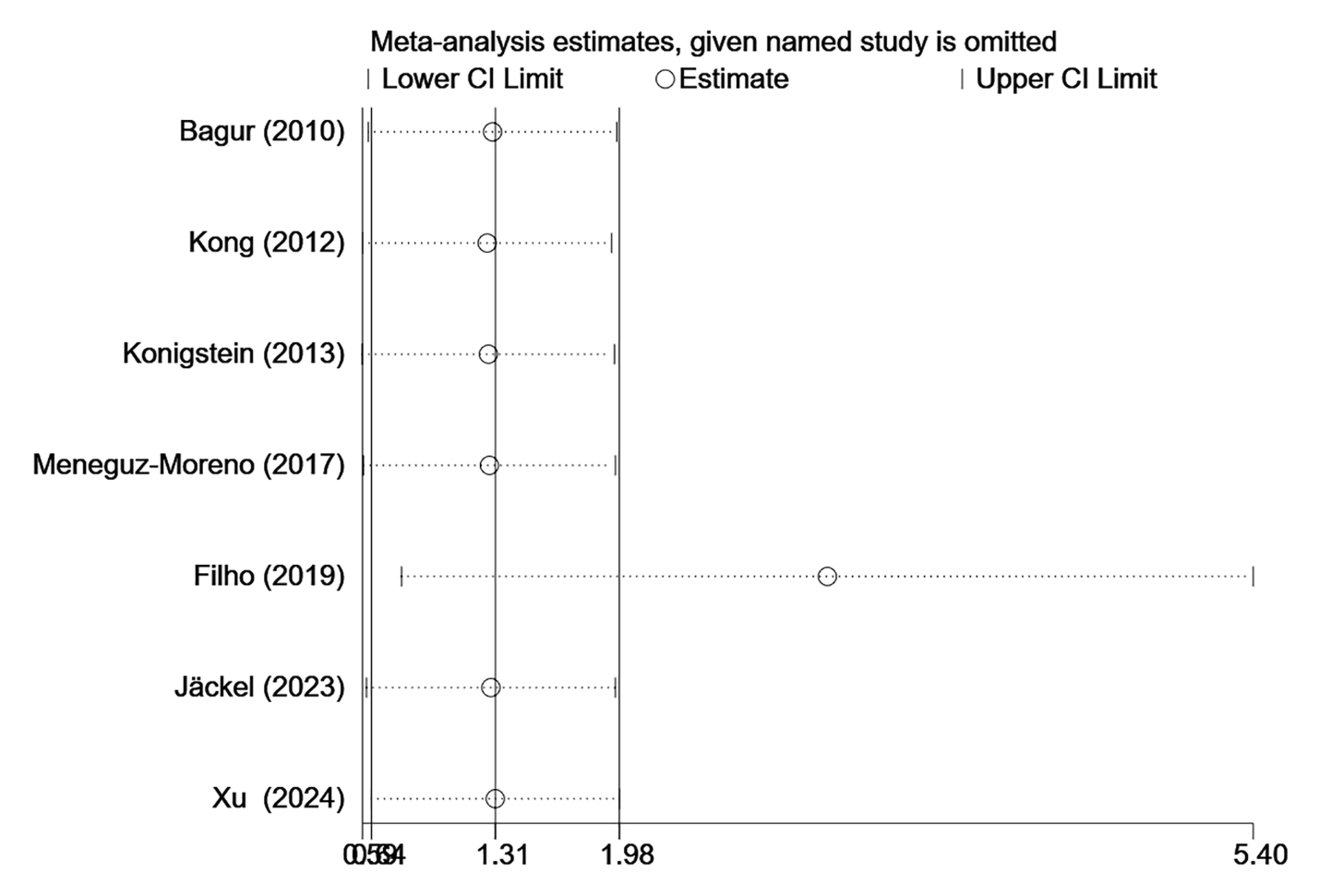


Figure S35 Hypertension Multivariable Sensitivity Analysis


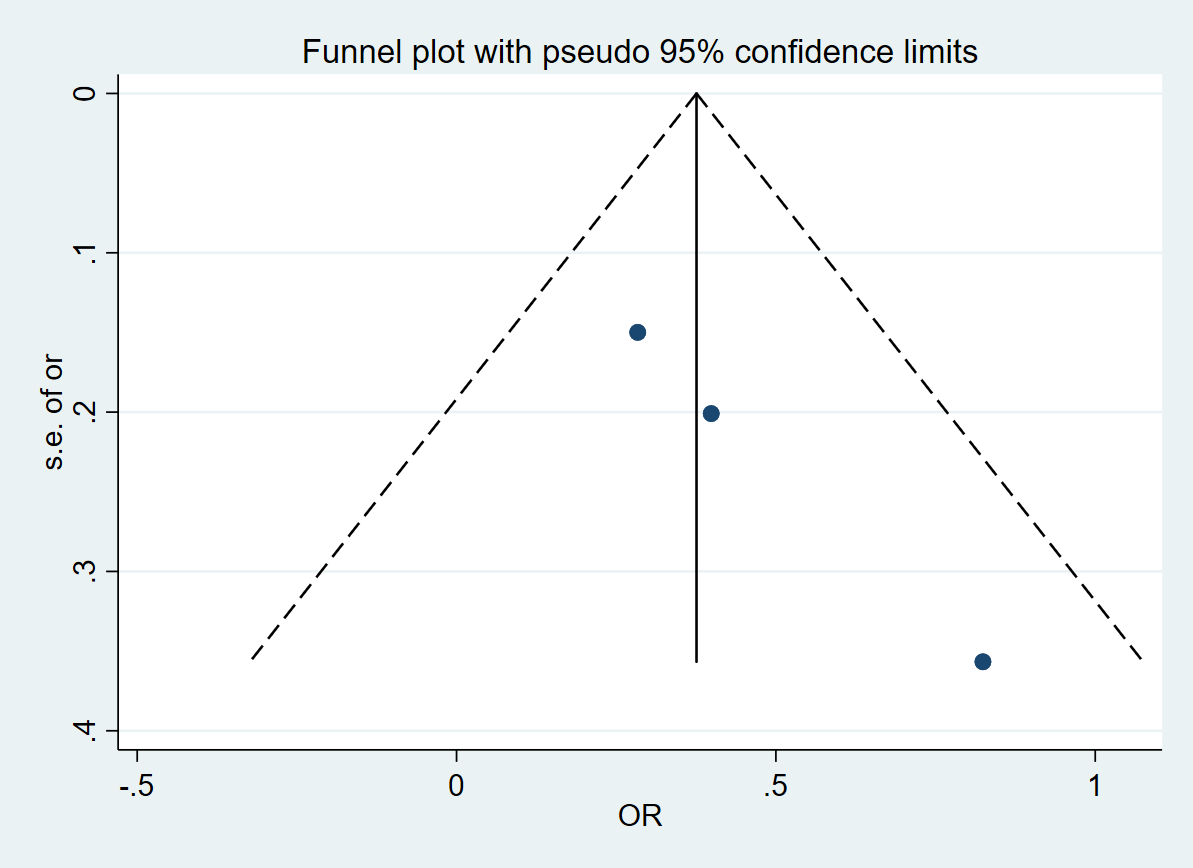


Figure S36 Coronary artery disease Multivariable Funnel Plotegger；Egger's Test P=0.033


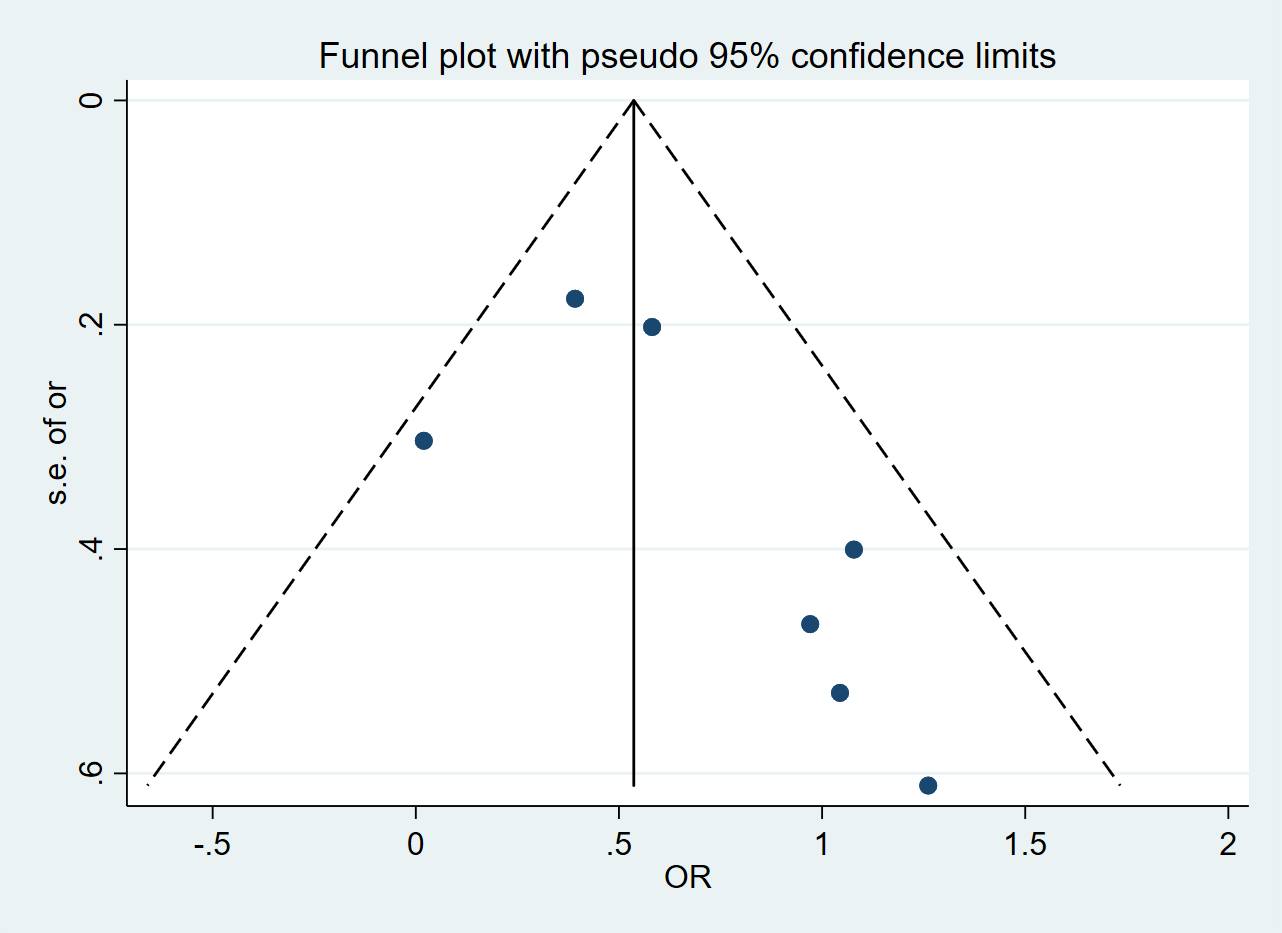


Figure S37 Peripheral Vascular Disease Multivariable Funnel Plotegger；Egger's Test P=0.111


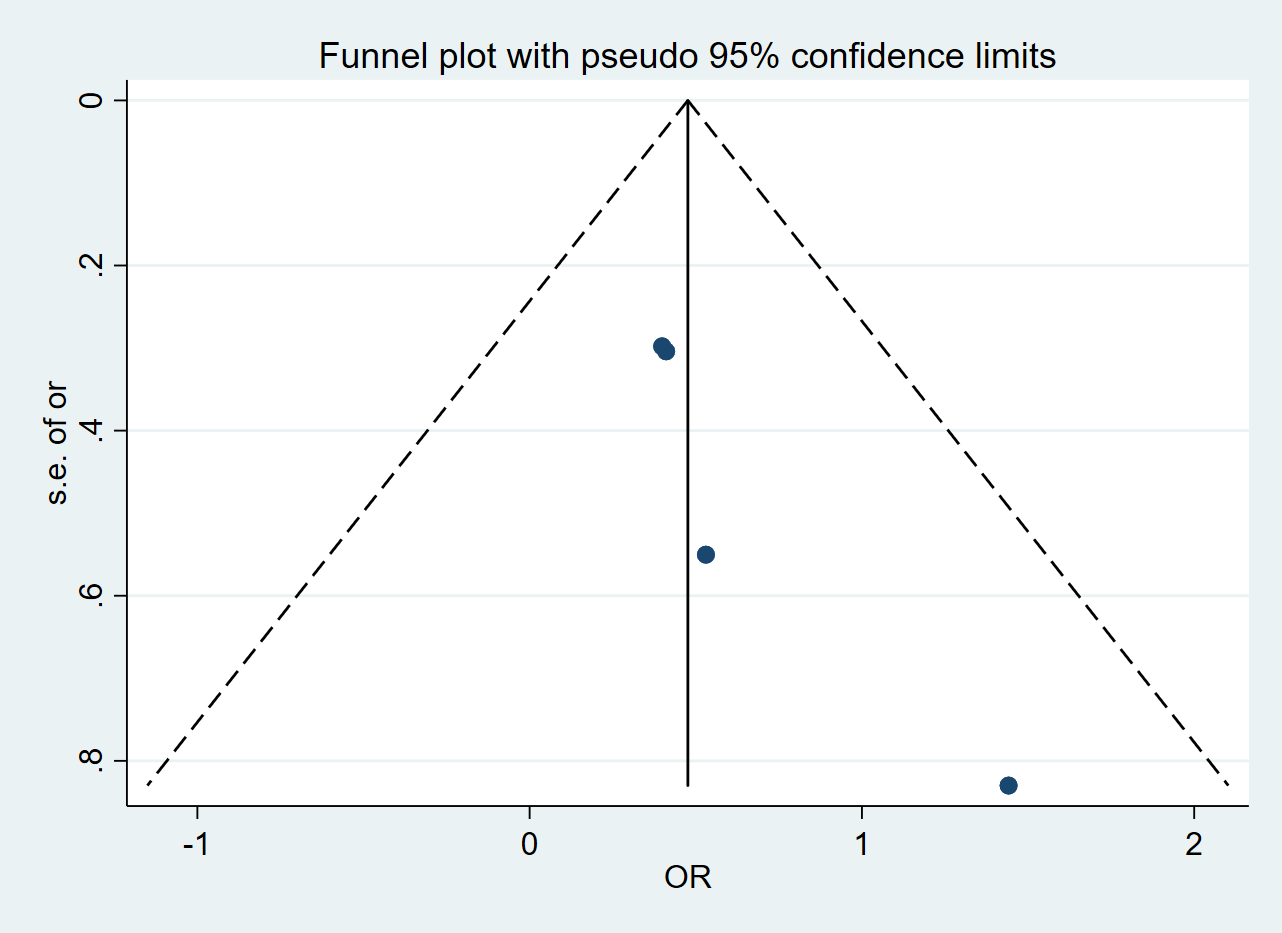


Figure S38 History of stroke Multivariable Funnel Plotegger；Egger's Test P=0.1


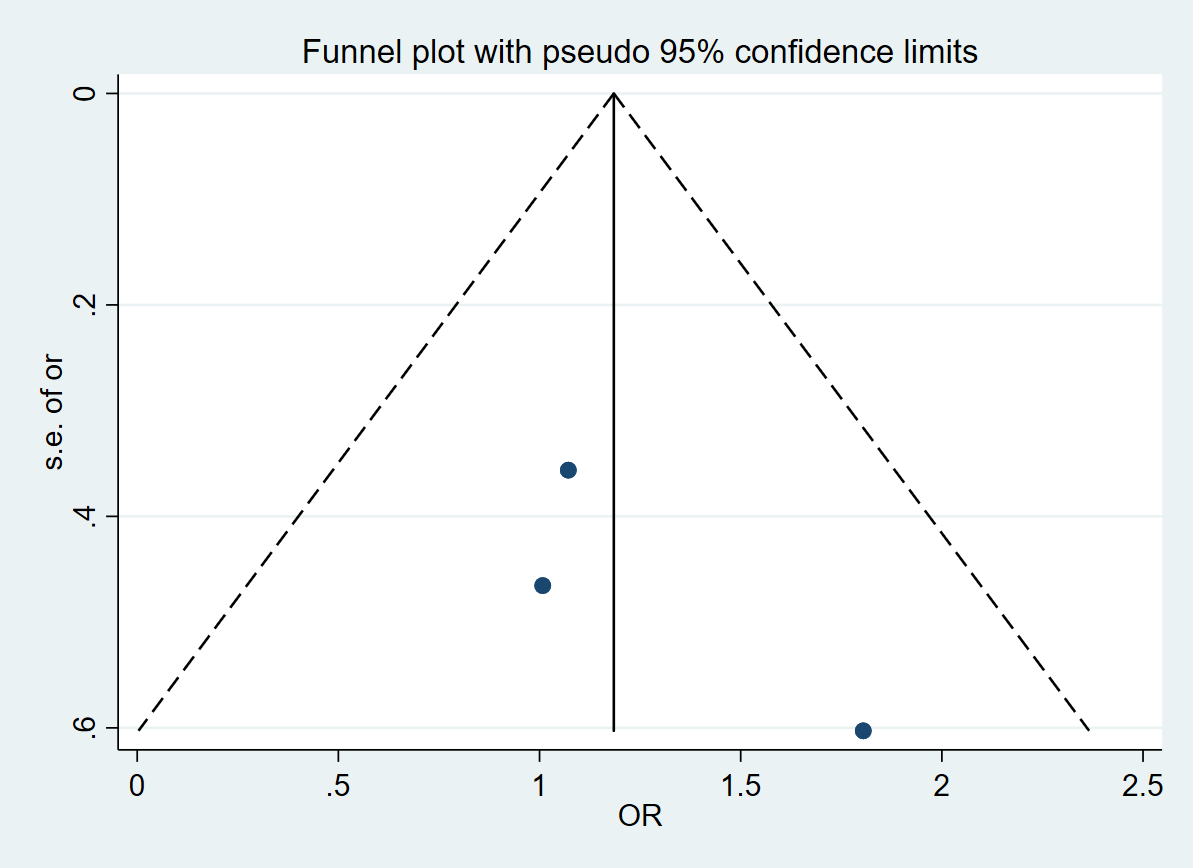


Figure S39 CKD Multivariable Funnel Plotegger；Egger's Test P=0.405


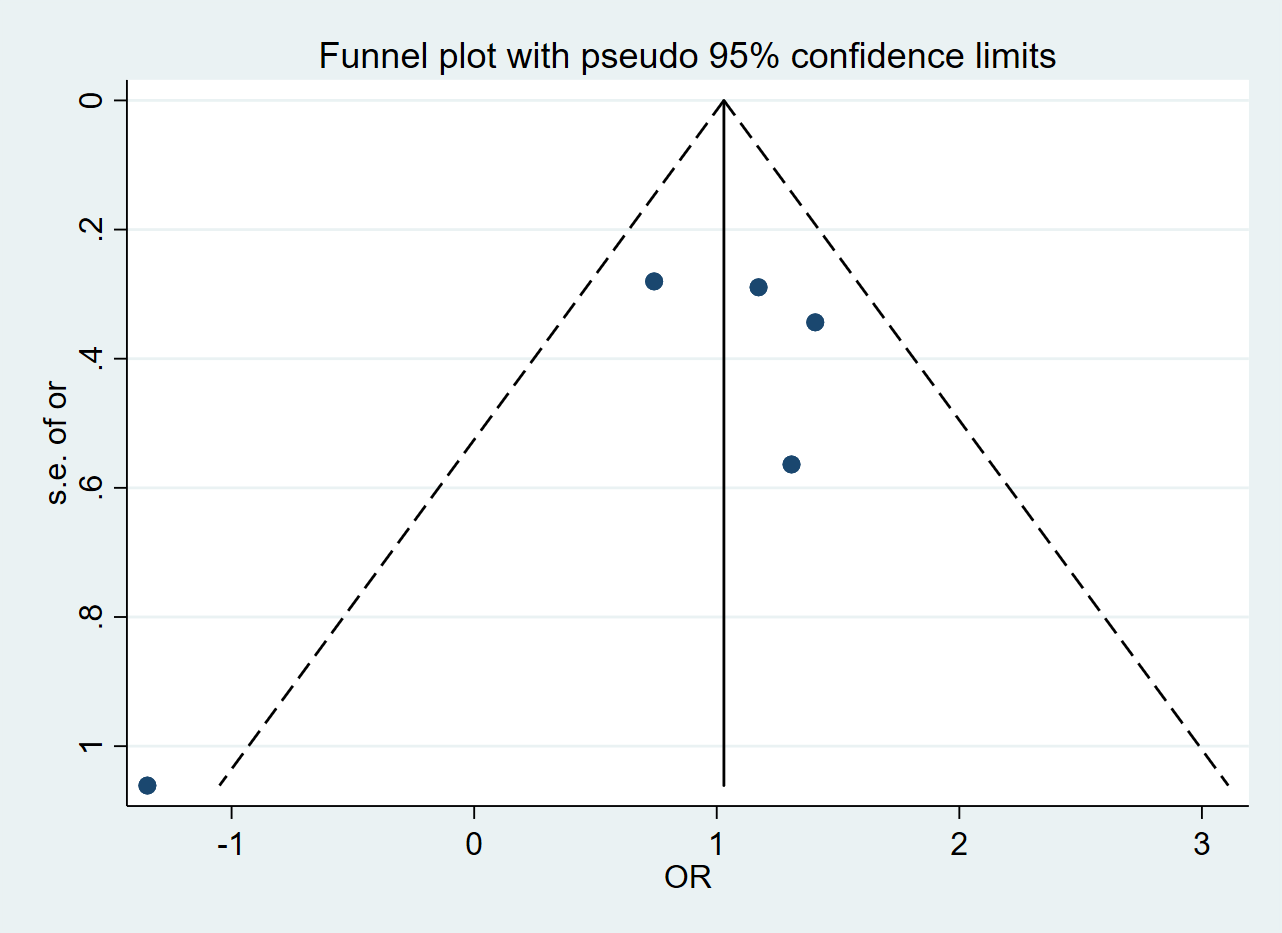


Figure S40 Serum creatinine level Multivariable Funnel Plotegger；Egger's Test P=0.381


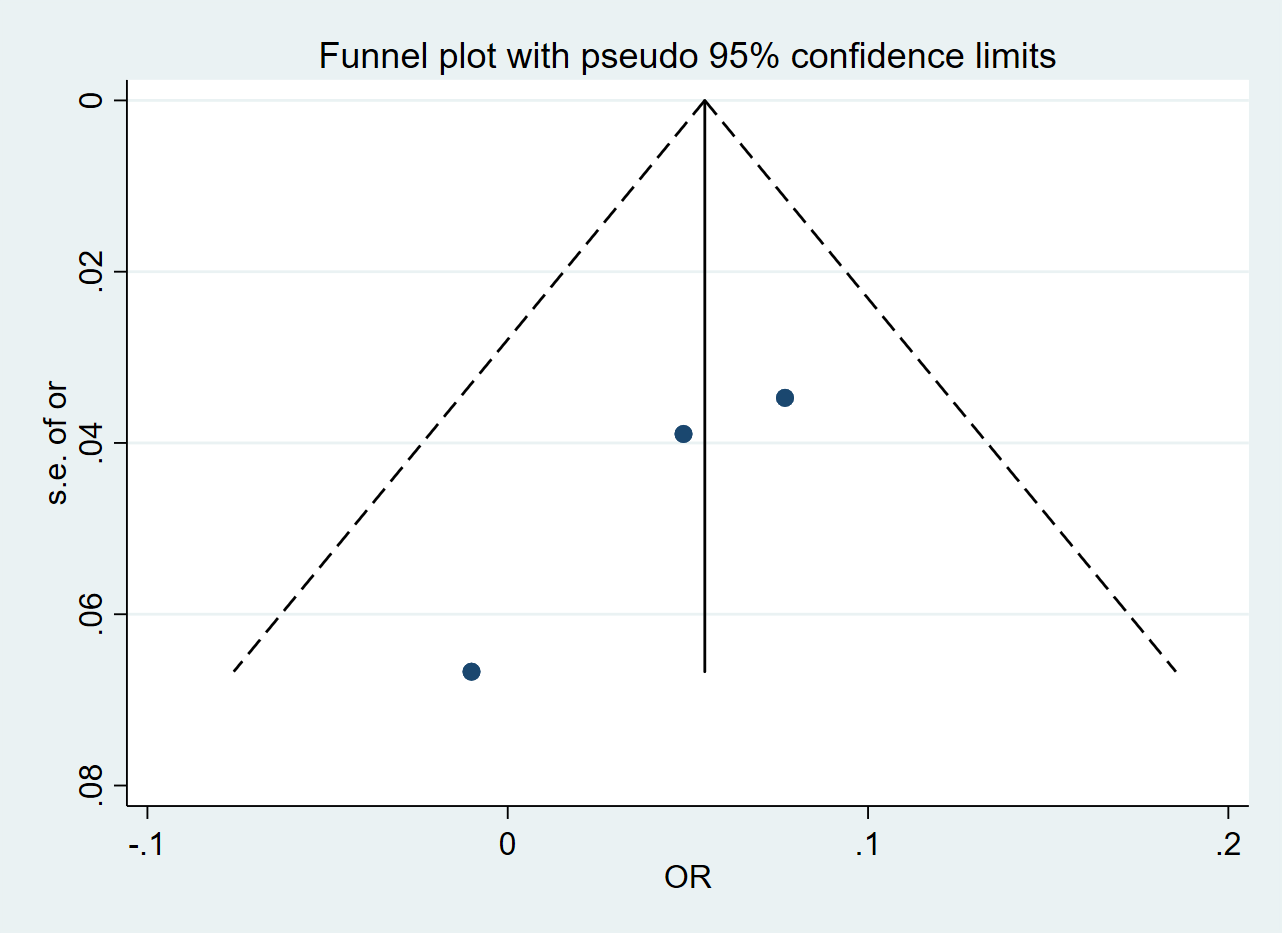


Figure S41 STS score Multivariable Funnel Plotegger；Egger's Test P=0.184


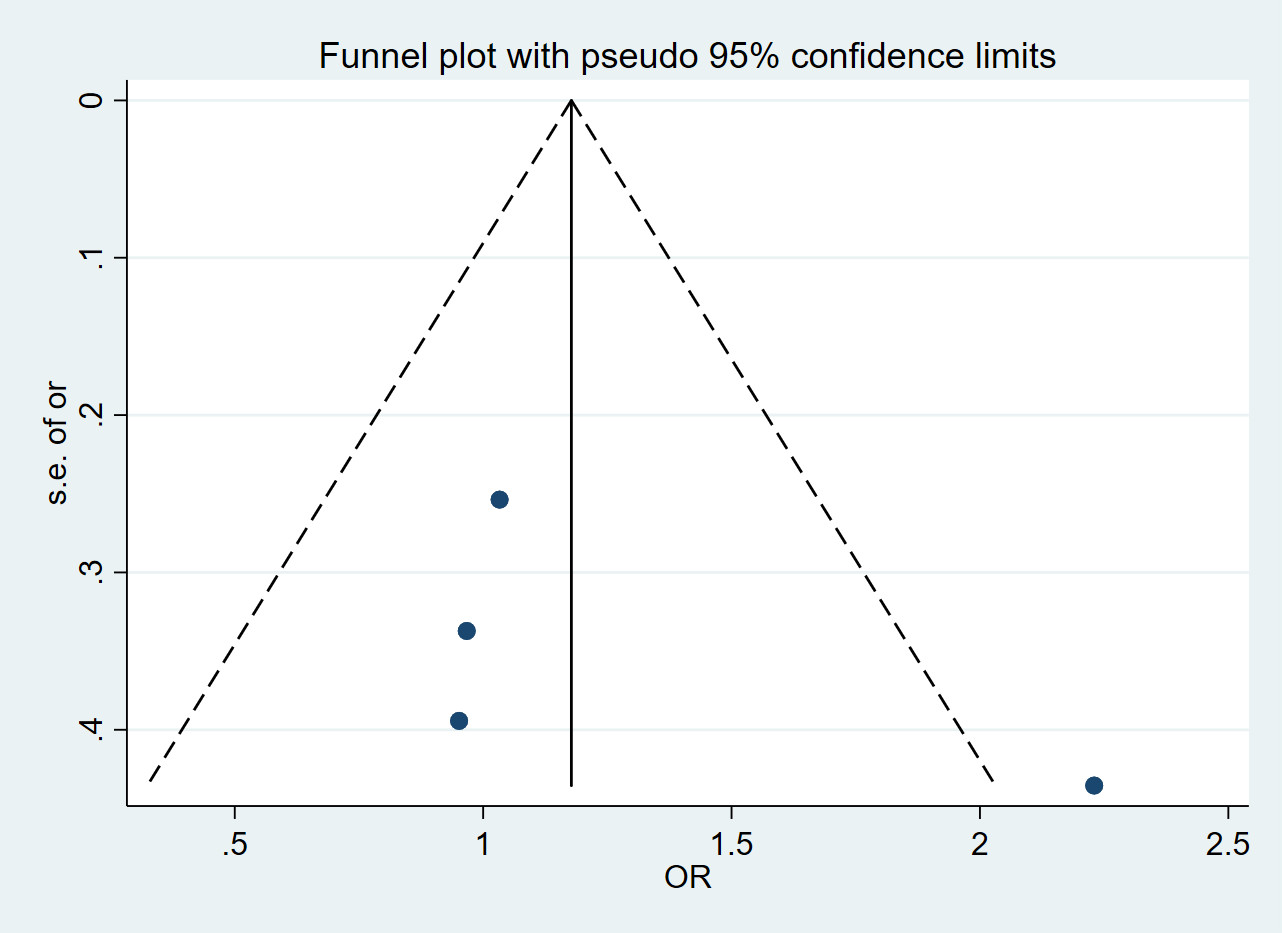


Figure S42 Transapical Access Multivariable Funnel Plotegger；Egger's Test P=0.409


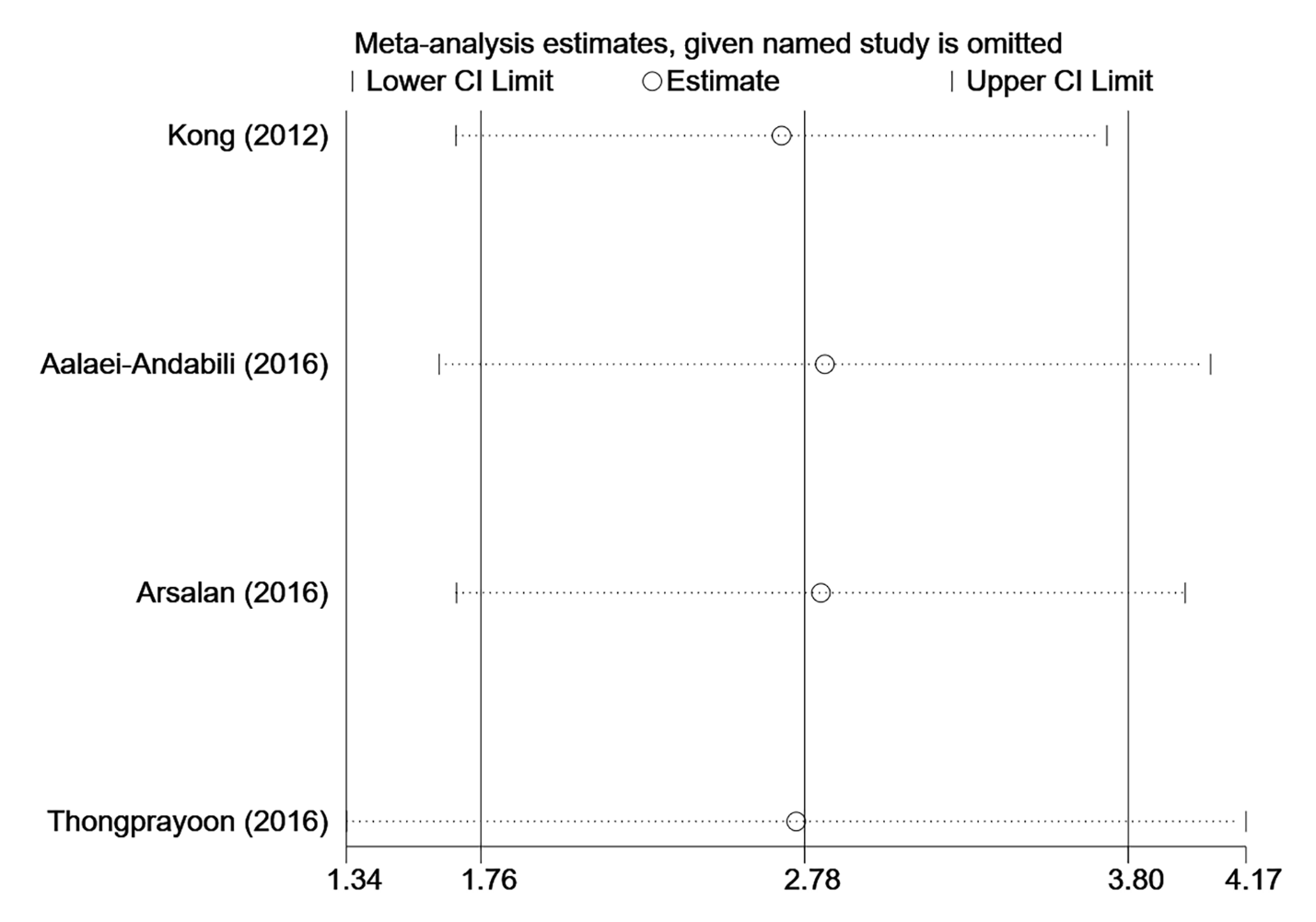


Figure S43 Transapical Access Multivariable Sensitivity Analysis


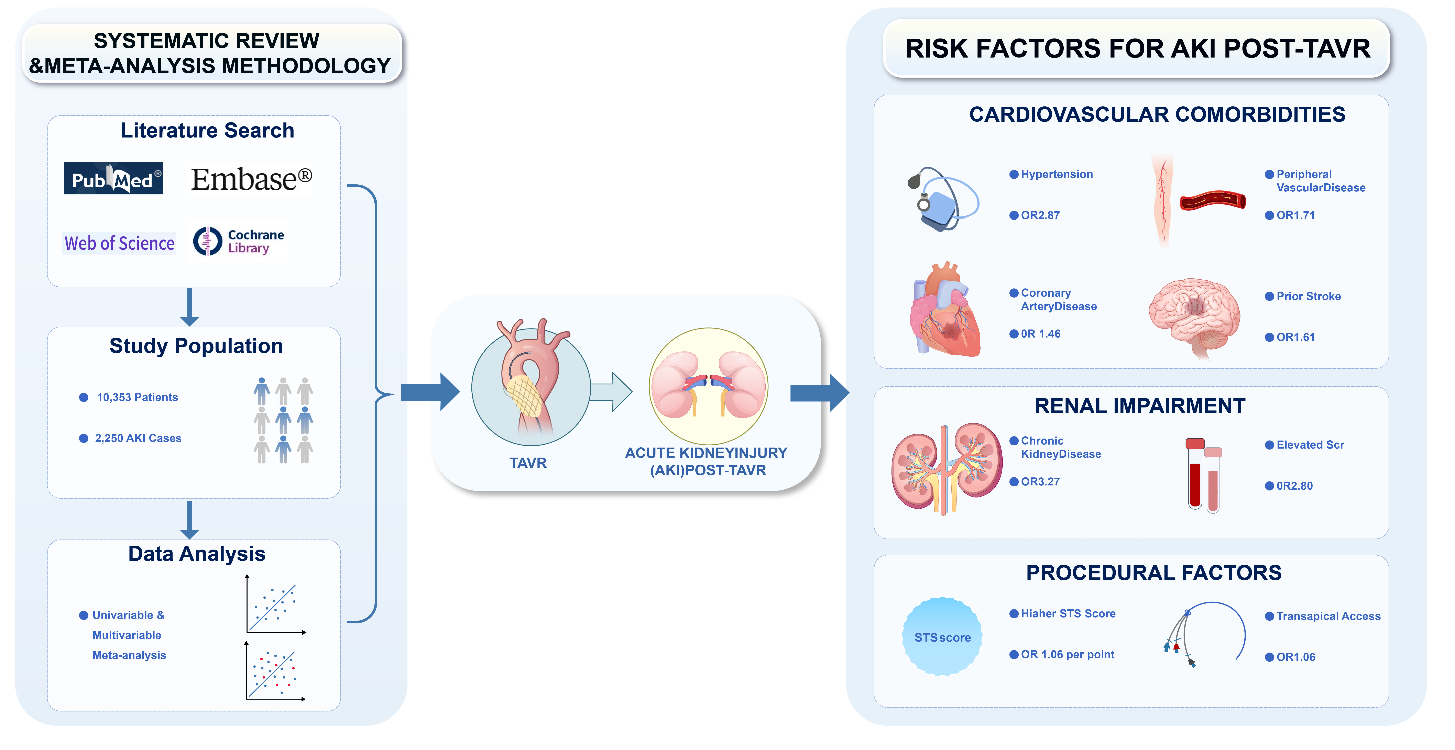


Figure S44 central illustration
